# Genus-Wide Characterization of Bumblebee Genomes Provides Insights into Their Evolution and Variation in Ecological and Behavioral Traits

**DOI:** 10.1093/molbev/msaa240

**Published:** 2020-09-18

**Authors:** Cheng Sun, Jiaxing Huang, Yun Wang, Xiaomeng Zhao, Long Su, Gregg W C Thomas, Mengya Zhao, Xingtan Zhang, Irwin Jungreis, Manolis Kellis, Saverio Vicario, Igor V Sharakhov, Semen M Bondarenko, Martin Hasselmann, Chang N Kim, Benedict Paten, Luca Penso-Dolfin, Li Wang, Yuxiao Chang, Qiang Gao, Ling Ma, Lina Ma, Zhang Zhang, Hongbo Zhang, Huahao Zhang, Livio Ruzzante, Hugh M Robertson, Yihui Zhu, Yanjie Liu, Huipeng Yang, Lele Ding, Quangui Wang, Dongna Ma, Weilin Xu, Cheng Liang, Michael W Itgen, Lauren Mee, Gang Cao, Ze Zhang, Ben M Sadd, Matthew W Hahn, Sarah Schaack, Seth M Barribeau, Paul H Williams, Robert M Waterhouse, Rachel Lockridge Mueller

**Affiliations:** 1 Institute of Apicultural Research, Chinese Academy of Agricultural Sciences, Beijing, China; 2 School of Life Sciences, Chongqing University, Chongqing, China; 3 Division of Biological Sciences, University of Montana, Missoula, MT; 4 State Key Laboratory of Agricultural Microbiology, Huazhong Agricultural University, Wuhan, China; 5 Fujian Provincial Key Laboratory of Haixia Applied Plant Systems Biology, Fujian Agriculture and Forestry University, Fuzhou, China; 6 MIT Computer Science and Artificial Intelligence Laboratory, Cambridge, MA; 7 Broad Institute of MIT and Harvard, Cambridge, MA; 8 Institute of Atmospheric Pollution Research-Italian National Research Council C/O Department of Physics, University of Bari, Bari, Italy; 9 Department of Entomology, Virginia Polytechnic and State University, Blacksburg, VA; 10 Department of Cytology and Genetics, Tomsk State University, Tomsk, Russian Federation; 11 Department of Livestock Population Genomics, Institute of Animal Science, University of Hohenheim, Stuttgart, Germany; 12 UC Santa Cruz Genomics Institute, University of California Santa Cruz, Santa Cruz, CA; 13 Deutsches Krebsforschungszentrum, Heidelberg, Germany; 14 Shenzhen Branch, Guangdong Laboratory for Lingnan Modern Agriculture, Genome Analysis Laboratory of the Ministry of Agriculture, Agricultural Genomics Institute at Shenzhen, Chinese Academy of Agricultural Sciences, Shenzhen, China; 15 BGI Genomics, BGI-Shenzhen, Shenzhen, China; 16 China National Center for Bioinformation & Beijing Institute of Genomics, Chinese Academy of Sciences, Beijing, China; 17 College of Pharmacy and Life Science, Jiujiang University, Jiujiang, China; 18 Department of Ecology and Evolution, University of Lausanne, and Swiss Institute of Bioinformatics, Lausanne, Switzerland; 19 Department of Entomology, University of Illinois at Urbana-Champaign, Champaign, IL; 20 Department of Medical Microbiology and Immunology, Genome Center, and MIND Institute, University of California Davis, Davis, CA; 21 Institute of Sericultural and Apiculture, Yunnan Academy of Agricultural Sciences, Mengzi, China; 22 Department of Biology, Colorado State University, Fort Collins, CO; 23 Department of Ecology, Evolution and Behaviour, Institute of Integrative Biology, University of Liverpool, Liverpool, United Kingdom; 24 School of Biological Sciences, Illinois State University, Normal, IL; 25 Department of Biology, Indiana University, Bloomington, IN; 26 Department of Computer Science, Indiana University, Bloomington, IN; 27 Department of Biology, Reed College, Portland, OR; 28 Department of Life Sciences, Natural History Museum, London, United Kingdom

**Keywords:** *Bombus*, insect diversity, genome assembly, genome evolution, gene family evolution

## Abstract

Bumblebees are a diverse group of globally important pollinators in natural ecosystems and for agricultural food production. With both eusocial and solitary life-cycle phases, and some social parasite species, they are especially interesting models to understand social evolution, behavior, and ecology. Reports of many species in decline point to pathogen transmission, habitat loss, pesticide usage, and global climate change, as interconnected causes. These threats to bumblebee diversity make our reliance on a handful of well-studied species for agricultural pollination particularly precarious. To broadly sample bumblebee genomic and phenotypic diversity, we de novo sequenced and assembled the genomes of 17 species, representing all 15 subgenera, producing the first genus-wide quantification of genetic and genomic variation potentially underlying key ecological and behavioral traits. The species phylogeny resolves subgenera relationships, whereas incomplete lineage sorting likely drives high levels of gene tree discordance. Five chromosome-level assemblies show a stable 18-chromosome karyotype, with major rearrangements creating 25 chromosomes in social parasites. Differential transposable element activity drives changes in genome sizes, with putative domestications of repetitive sequences influencing gene coding and regulatory potential. Dynamically evolving gene families and signatures of positive selection point to genus-wide variation in processes linked to foraging, diet and metabolism, immunity and detoxification, as well as adaptations for life at high altitudes. Our study reveals how bumblebee genes and genomes have evolved across the *Bombus* phylogeny and identifies variations potentially linked to key ecological and behavioral traits of these important pollinators.

## Introduction

Bumblebees (Hymenoptera: Apidae) are a group of pollinating insects comprising the genus *Bombus*, which are economically important for crop pollination (Velthuis and Van Doorn 2006; Garibaldi et al. 2013; Martin et al. 2019). Bumblebees are also ecologically important pollinators, serving as the sole or predominant pollinators of many wild plants (Fontaine et al. 2005; Goulson et al. 2008). They are particularly charismatic social insects that exhibit complex behaviors such as learning through observation (Alem et al. 2016) and damaging leaves to stimulate earlier flowering (Pashalidou et al. 2020). Global and local environmental changes have resulted in some species declining in range and abundance and others remaining stable or even increasing (Cameron et al. 2011; Bartomeus et al. 2013; Koch et al. 2015; Cameron and Sadd 2020). Decline in bumblebee abundance and distribution resulting from habitat loss, pathogen transmission, climate change, and agrochemical exposure is threatening pollination services to both wild plants and crops, raising concerns for bumblebees, the plant species they service, food security, and ecosystem stability (Grixti et al. 2009; Williams and Osborne 2009; Potts et al. 2010; Goulson et al. 2015; Cameron and Sadd 2020; Soroye et al. 2020).

Bumblebees comprise ∼250 extant species classified into 15 subgenera (Williams 1998; Cameron et al. 2007; Williams et al. 2018). The initial diversification of *Bombus* lineages occurred ∼25–40 Ma, near the Eocene–Oligocene boundary ∼34 Ma (Williams 1998; Hines 2008). Bumblebees display considerable interspecific diversity in morphology, color patterning, food preference, and pathogen incidence and exhibit diverse life histories and ecologies (Williams 1994; Sikora and Kelm 2012; Persson et al. 2015; Arbetman et al. 2017; Cameron and Sadd 2020). Members of the subgenus *Mendacibombus*, the sister group to all other extant bumblebees, are high-elevation specialists with distributions centered on the Qinghai–Tibetan plateau (Williams et al. 2018). Species in the subgenus *Psithyrus* exhibit social parasitism; they do not have a worker caste, and they feed on food collected by workers of their host species (Lhomme and Hines 2019). Bumblebees are distributed across the globe, from Greenland to the Amazon Basin and from sea level to altitudes of 5,640 m in the Himalayas, where they occupy diverse habitats, from alpine meadows to lowland tropical forest (Williams 1985; Williams et al. 2018). Much remains to be learned about bumblebees. For example, little is known about the underlying genetic and genomic variation that gives rise to these diverse phenotypes, including their differential responses to changing environments.

About half of the ∼250 extant species, representing 14 of the 15 *Bombus* subgenera, are found in China, making it a hotspot of bumblebee species richness (Williams 1998; Williams et al. 2017). To broadly sample the genomic and phenotypic diversity of bumblebees, we selected representative species from China for whole-genome sequencing based on their phylogeny, ecology, behavior, geography, and specimen availability. To complete subgenus sampling, we additionally selected *Bombus polaris* from the subgenus *Alpinobombus*, which is endemic to Arctic/subarctic regions. In total, we performed de novo sequencing and assembly of the genomes of 17 bumblebee species, representing all 15 subgenera within the genus *Bombus*. Integrating these data sets with two previously published bumblebee genomes, we performed comparative analyses of genome structures, genome contents, and gene evolutionary dynamics across the phylogeny. Our results characterize patterns of molecular and genomic evolution across the *Bombus* phylogeny and provide the first genus-wide quantification of genetic and genomic variation potentially underlying key ecoethological traits.

## Results

### High-Quality Genomic Resources for All 15 *Bombus* Subgenera

Sequencing and assembly strategies resulted in high-quality genomic resources with 12 scaffold-level and five chromosome-level genome assemblies (table 1). Criteria including phylogenetic position, species traits, and geographic distribution were applied to select species for whole-genome sequencing from across the genus. For the five species for which sufficient samples could be collected, high-throughput chromatin conformation capture (Hi-C) (Belton et al. 2012) was used to produce chromosome-level genome assemblies (table 1). A total of 17 species were selected (supplementary table S1 and fig. S1, Supplementary Material online), which span all 15 subgenera of the genus *Bombus* (Williams et al. 2008). Among these, two species (*Bombus superbus* and *B. waltoni*) are from *Mendacibombus*, the earliest split in the *Bombus* phylogeny; four species (*B. superbus*, *B. waltoni*, *B. skorikovi*, and *B. difficillimus*) inhabit high elevations (>4,000 m above sea level); two species (*B. turneri* and *B. skorikovi*) exhibit social parasitism; three species (*B. pyrosoma*, *B. picipes*, and *B. superbus*) are endemic to China; and one species (*B. polaris*) is endemic to Arctic/subarctic regions (Williams et al. 2019). In addition, species traits including range size, tongue length, parasite incidence, and decline status vary across the selected species (Williams 1994; Arbetman et al. 2017; Cameron and Sadd 2020).

**Table 1.** Genome Assembly Results of the 17 Newly Sequenced Bumblebees.

	Contig Size (Mb)	Contig N50 (kb)	Scaffold Size (Mb)	Scaffold N50 (Mb)	Chromosome Size (Mb)	Chromosome N50 (Mb)
*Bombus superbus*	229.84	441.61	230.16	6.90	NA	NA
*Bombus waltoni*	230.89	430.54	231.17	4.66	NA	NA
*Bombus confusus*	238.52	227.26	239.12	3.26	NA	NA
*Bombus haemorrhoidalis*	239.34	572.47	239.59	4.74	240.54	15.09
*Bombus ignitus*	240.60	374.12	241.36	3.02	242.57	15.19
*Bombus skorikovi*	241.25	225.53	242.05	4.34	NA	NA
*Bombus opulentus*	241.99	267.78	242.38	2.42	NA	NA
*Bombus turneri*	242.39	212.53	243.01	4.34	243.11	9.70
*Bombus soroeensis*	243.19	244.99	243.68	2.12	NA	NA
*Bombus polaris*	245.17	152.35	245.82	2.25	NA	NA
*Bombus breviceps*	246.03	578.55	246.41	4.04	248.12	14.71
*Bombus cullumanus*	246.56	422.80	247.01	4.58	NA	NA
*Bombus difficillimus*	247.45	177.31	248.33	2.07	NA	NA
*Bombus consobrinus*	248.56	284.90	249.09	4.77	NA	NA
*Bombus pyrosoma*	251.86	472.32	252.70	6.07	254.80	15.22
*Bombus picipes*	253.31	185.91	254.01	5.88	NA	NA
*Bombus sibiricus*	261.72	253.94	262.49	3.14	NA	NA

Note.—kb, kilobase; Mb, megabase; NA, not applicable.

Sequencing and assembly strategies included generating two Illumina sequencing data sets for each species: 1) overlapping paired-end reads (2× 250 bp) from one small-insert fragment library using one single haploid drone per species (insert size: 400 or 450 bp) and 2) paired-end reads (2× 150 bp) from four large-insert jump libraries using 3–5 individuals per species (insert sizes: 4, 6, 8, and 10 kb, respectively; supplementary table S2, Supplementary Material online). Whole-genome overlapping paired-end reads from fragment libraries were assembled into continuous sequences (contigs) using the software DISCOVAR de novo (Love et al. 2016), then scaffolded with reads from jump libraries using the software BESST (Sahlin et al. 2014). The resulting assemblies have a mean contig N50 of 325 kb, ranging up to 579 kb for *B. breviceps*; the mean scaffold N50 is 4.0 Mb, ranging up to 6.9 Mb for *B. superbus* (table 1). Genome assembly quality in terms of expected gene content was evaluated by Benchmarking Universal Single-Copy Ortholog (BUSCO) analysis (Waterhouse et al. 2018), which showed high BUSCO completeness scores (average 99.0%, from 97.5% to 99.6%; supplementary fig. S2, Supplementary Material online) for all genomes.

Genome annotation resulted in total protein-coding gene predictions per species ranging from 14,027 to 16,970 (mean = 15,838, standard deviation = 908; supplementary table S3, Supplementary Material online). These were annotated using the MAKER pipeline (Cantarel et al. 2008), based on ab initio gene predictions, transcript evidence, and homologous protein evidence. Gene counts are similar to those of 12 drosophilid species (mean = 15,361, SD = 852; Clark et al. 2007) but are higher than those of 19 anophelines (mean = 13,110, Sd = 1,397) (Neafsey et al. 2015), and they do not correlate significantly with assembly contiguity (*P* = 0.1757; supplementary fig. S3, Supplementary Material online). Between 7,299 and 8,135 genes were assigned at least one Gene Ontology (GO) term and 9,431–10,578 genes were annotated with at least one protein domain (supplementary table S3, Supplementary Material online). BUSCO analysis of the annotated genes also showed high completeness scores for all species (supplementary fig. S4, Supplementary Material online). Furthermore, comprehensive miRNA, tRNA, and lncRNA gene prediction revealed an average of 93, 306, and 3,353 genes, respectively (supplementary table S3, Supplementary Material online). Finally, transposable element (TE) annotation showed that the total TE content ranged from 9.66% (22.2 Mb) in *B. superbus* to 17.88% (46.9 Mb) in *B. sibiricus* (supplementary table S4, Supplementary Material online).

### Genome-Scale Phylogeny of Bumblebees

**Fig. 1.**
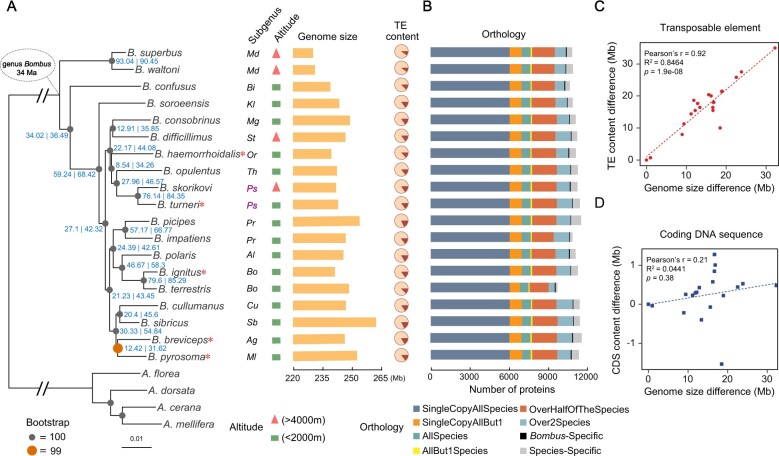
Phylogenetic, genomic, and proteomic comparisons of 19 bumblebee species representing all 15 *Bombus* subgenera. (*A*) From left to right: the maximum-likelihood molecular species phylogeny built from 2,918 concatenated single-copy orthologous groups from all sequenced bumblebees and honeybee outgroups using IQ-TREE. Node labels in blue are of the following format: gCFs | sCFs. Branches scaled by relative number of substitutions; red asterisks after species names indicate the five species with chromosomal-level assemblies; the subgenus that each bumblebee species belongs to (Md, *Mendacibombus*; Bi, *Bombias*; Kl, *Kallobombus*; Mg, *Megabombus*; St, *Subterraneobombus*; Or, *Orientalibombus*; Th, *Thoracobombus*; Ps, *Psithyrus*; Cu, *Cullumanobombus*; Sb, *Sibiricobombus*; Ag, *Alpigenobombus*; Ml, *Melanobombus*; Pr, *Pyrobombus*; Al, *Alpinobombus*; Bo, *Bombus*); altitude of species collection site (red triangle: extreme high altitude; green rectangle: low-altitude); and genome assembly size of each sequenced species; fraction of TEs (brown) in each genome. (*B*) Bar plots show total gene counts for each bumblebee partitioned according to their orthology profiles, from ancient genes found across bumblebees to lineage-restricted and species-specific genes. (*C*, *D*) The contribution of TE and CDS to genome size variation across bumblebees, respectively. Differences in the total content of TEs (*C*) and CDS (*D*) of the 19 genomes relative to that of *Bombus superbus* (which has the smallest genome assembly size) are plotted against their genome size differences (relative to that of *B. superbus*).

The species-level molecular phylogeny (fig. 1*A*) estimated from maximum-likelihood analysis with IQ-TREE (Minh, Schmidt, et al. 2020) is largely consistent with previously inferred phylogenetic relationships of the 15 subgenera based on five genes (Cameron et al. 2007; Williams et al. 2008), showing only two topological differences. The results support previous conclusions that 1) subgenus *Mendacibobus* (labeled *Md* in fig. 1*A*) is the sister group to all the other subgenera and 2) species of *Psithyrus* (labeled *Ps* in purple in fig. 1*A*) are within the genus *Bombus*, arguing *Psithyrus* should not be named as an independent genus. The species phylogeny was built from the concatenated aligned protein sequences of 2,918 universal single-copy orthologs from 19 bumblebee species (17 from the current study, two published previously: *Bombus terrestris* and *B. impatiens* [Sadd et al. 2015]) and four honeybee species (*Apis florea*, *A. dorsata* [Oppenheim et al. 2020], *A. cerana* [Park et al. 2015], and *A. mellifera* [Weinstock et al. 2006]), with orthologous groups delineated using the OrthoDB software (Kriventseva et al. 2015). Complementary analysis with ASTRAL based on maximum-likelihood gene trees (Zhang et al. 2018) resulted in an identical species tree with the exception of the placement of *B. pyrosoma*, which no longer forms a monophyletic pairing with *B. breviceps*, but rather forms an asymmetrical four-taxa clade with *B. breviceps*, *B. sibricus*, and *B. cullumanus* (supplementary fig. S5, Supplementary Material online). The tendency for maximum-likelihood concatenation to return a symmetrical four-taxa topology while ASTRAL returns an asymmetrical topology (as observed here) is a known shortcoming of maximum-likelihood concatenation in the presence of incomplete lineage sorting (ILS) (Kubatko and Degnan 2007; Mendes and Hahn 2018), implying that the ASTRAL topology is likely the correct topology.

However, inspecting the gene trees reveals extreme levels of discordance: none of their topologies matches the topology of the tree inferred from concatenation (supplementary tables S5 and S6, Supplementary Material online), and nearly every gene tree has a unique topology (supplementary table S7, Supplementary Material online). Such extreme levels of discordance have been observed previously in birds (Jarvis et al. 2014) and tomatoes (Pease et al. 2016) and have been attributed to a variety of sources, such as ILS and introgression (Maddison 1997). A lack of informative sites, only 24%, compared with 47% in a similar data set of 25 drosophilids (Da Lage et al. 2019), possibly due to the relatively recent diversification of bumblebees (Hines 2008), may also cause discordance. Gene and site concordance factor (sCF) analysis (Minh, Hahn, et al. 2020) was employed to quantify the amount of discordance between gene trees and the IQ-TREE species tree (node labels in fig. 1*A*). For each node in the IQ-TREE species tree, gene concordance factors (gCFs) reflect the percentage of gene trees that contain that node as defined by its descendant taxa, and sCFs reflect the percentage of informative sites that support that node via parsimony. On average across the *Bombus* phylogeny, nodes in the IQ-TREE species tree show a gCF of 38.4%, indicating that on average a node is present in only two-fifths of gene trees. More stringent filtering to use gene trees with the highest bootstrap support results in higher gCF values for all nodes (supplementary fig. S6, Supplementary Material online). Average sCF across *Bombus* nodes is 53.6%, meaning a little over half of informative sites in the gene alignments support the nodes of the IQ-TREE species tree (node labels in fig. 1*A*). These sCFs, the short internal branches of the species tree, and the strong correlation between them (supplementary fig. S7, Supplementary Material online) are consistent with ILS driving the observed gene tree discordance. The possible contribution of introgression to the observed discordance among gene trees was examined using tree topologies to calculate Δ as described in Huson et al. (2005) and Vanderpool et al. (2020) for each branch in the IQ-TREE and the ASTRAL species tree that showed a gCF of <95%. Using bootstrap sampling of gene trees to provide a null distribution (supplementary fig. S8, Supplementary Material online), no lineages in either species tree showed significantly high values of Δ, ruling out introgression as a source of discordance (supplementary fig. S9, Supplementary Material online). Because of the high levels of discordance, gene-level phylogenies are therefore used in all subsequent gene-based molecular evolution analyses because such discordance can bias inferences of substitutions when mapped onto a species tree (Mendes and Hahn 2016).

### Major Genomic Rearrangements in Social Parasites

**Fig. 2.**
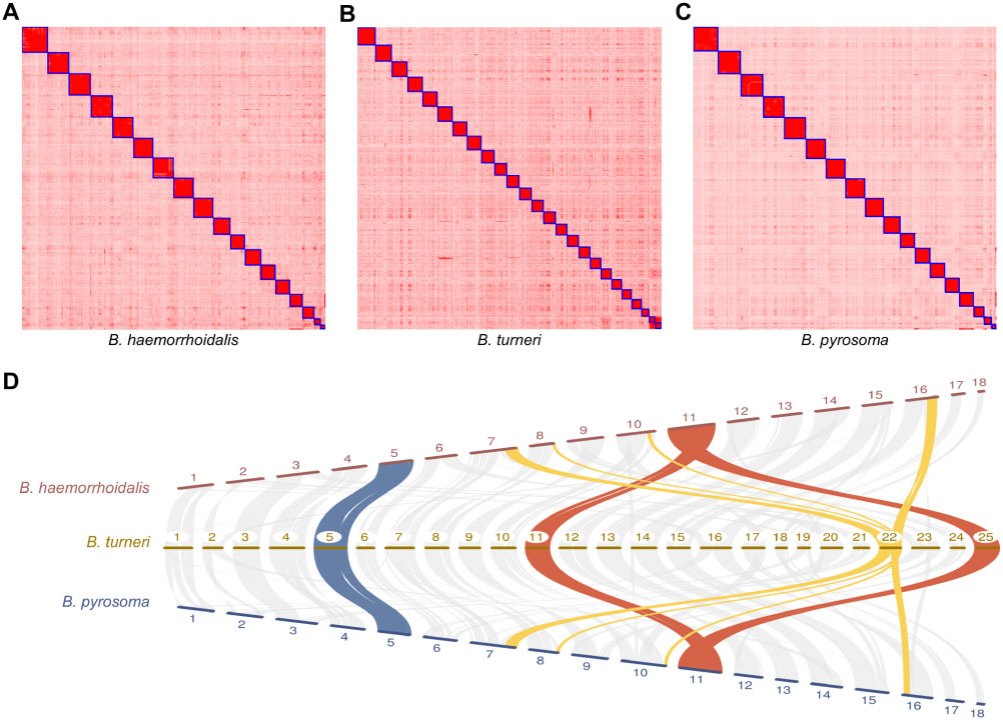
Chromosome number evolution in representative bumblebee species from three different subgenera. Hi-C contact heatmaps for *Bombus haemorrhoidalis* (*A*), *B. turneri* (*B*), and *B. pyrosoma* (*C*) show that the three species have 18, 25, and 18 chromosomes, respectively. The 18-chromosome karyotype is the inferred ancestral genome structure, with 25 chromosomes found in social parasite bumblebees of the subgenus *Psithyrus*. (*D*) Macrosynteny comparisons across *B. haemorrhoidalis*, *B. turneri*, and *B. pyrosoma* show how the 25 *B. turneri* chromosomes result from a combination of fission (red), fusion (yellow), and retention (blue) of ancestral chromosomes.

The five Hi-C genome assemblies indicate that four of the five subgenera have 18 chromosomes (fig. 2*A* and *C*; supplementary fig. S10*A* and *B*, Supplementary Material online), consistent with previous karyotypic analysis that inferred the ancestral chromosome number is 18 (Owen et al. 1995). However, the social parasite bumblebee, *B. turneri*, subgenus *Psithyrus*, has 25 chromosomes (fig. 2*B*), consistent with previous cytological work (Owen 1983). Despite the higher chromosome number, its genome size is within the range of other bumblebees (fig. 1*A* and table 1). Investigating macrosynteny relationships between *B. turneri* and the other species with chromosomal-level assemblies revealed three major processes that explain how a 25-chromosome karyotype was derived from the ancestral karyotype of 18 chromosomes. First, some chromosomes descended, structurally unchanged, from ancestral chromosomes (e.g., chromosome 5; fig. 2*D* in blue). Second, some originated by fission of an ancestral chromosome (e.g., 11 and 25 of *B. turneri* originated by the fission of ancestral chromosome 11; fig. 2*D* in red). Lastly, some are derived from fusions of two or more ancestral chromosome segments (e.g., *B. turneri* chromosome 22 was derived from the fusion of segments of ancestral chromosomes 7, 8, 10, and 16 [fig. 2*D* in gold]). Pairwise comparisons between *Psithyrus* and members of other subgenera reveal similar results and support the inference that the 25 chromosomes of the social parasite bumblebee result from a combination of fission, fusion, and retention of ancestral chromosomes (supplementary fig. S10, Supplementary Material online).

Rates of chromosome evolution, in terms of rearrangements relative to *B. terrestris*, were investigated for each of the five species with chromosome-level assemblies. Rearrangement rates in bumblebees range from 0.0016 to 0.0075 inversions/Mb/My (supplementary table S8, Supplementary Material online), which are much lower than those of drosophilids (0.013–0.159 inversions/Mb/My) and anophelines (0.052–0.068 inversions/Mb/My) (von Grotthuss et al. 2010; Neafsey et al. 2015). Thus, although bumblebee genomes have a high recombination rate (Wilfert et al. 2007), their rates of chromosome evolution are relatively slow, which is further supported by the observed high synteny contiguity across species (average 88%, from 80% to 95%; supplementary table S9, Supplementary Material online).

### TEs Drive Genome Size Variation

Genome assembly sizes (haploid) range from 230 Mb in *B. superbus* to 262 Mb in *B. sibiricus* (fig. 1*A*). Ancestral genome size inference of bumblebees produced an estimate of 230–231 Mb, similar to that of members of the subgenus *Mendacibombus*, but smaller than the genomes of all other extant bumblebees surveyed (supplementary fig. S11, Supplementary Material online). Comparing genome size differences with relative content of TEs, simple sequence repeats, and coding DNA sequences (CDS) shows that TE content explains a majority of the differences across bumblebees (Pearson correlation *R* = 0.92, *P *=* *1.9e-08, *R*^2^ = 0.85; fig. 1*C* and *D*; supplementary fig. S12, Supplementary Material online). *Mendacibombus* species have a smaller genome size than other species (fig. 1*A*), and TEs that transposed in non-*Mendacibombus* species after divergence from *Mendacibombus* show copy numbers ranging from 1,992 to 4,755 (supplementary fig. S13, Supplementary Material online), supporting the contribution of TEs to genome size evolution. Furthermore, TE proliferation history analysis indicated that all non-*Mendacibombus* species have more recent TE amplification peaks (supplementary fig. S14, Supplementary Material online), consistent with increased TE activity driving genome size increases.

The genomic distributions of TEs include 1,074–1,786 TE loci that overlap with the coding regions of protein-coding genes (supplementary table S10, Supplementary Material online). In total, 352 of these genes are universal single-copy orthologs across the 19 bumblebees whose overall d*N*/d*S* values are all <1 (supplementary table S11, Supplementary Material online), suggesting long-term functional constraints. One case of a putative ancient and maintained chimeric TE-gene fusion involves a gene with single-copy orthologs across the 19 bumblebees where the C-terminus of the proteins match the sequence of a reverse transcriptase of an R1 retrotransposon (supplementary fig. S15, Supplementary Material online). Aligned reads from RNA-sequencing data continue with similar coverage levels into the putatively TE-derived region at the 3′-end of the gene, supporting the prediction and the expression of the full chimera. TE activity has therefore contributed to the evolution of the bumblebee protein-coding gene repertoire. In addition, there are thousands of TEs located within 1 kb of a gene in each species (supplementary table S10, Supplementary Material online), and, in *B. terrestris*, 278 such TEs colocate with open chromatin regions detected by ATAC-seq (supplementary table S12, Supplementary Material online), suggesting those TEs may have become incorporated into regulatory sequences.

### Gene Content Evolution Reflects Foraging and Diet Diversity

Orthology delineation results indicate that a majority of genes are found in one or more copies in nearly all species across bumblebees (fig. 1*B*). These include 53 orthologous groups specific to the *Bombus* genus, which are present in all 19 bumblebees but absent in all four honeybees (fig. 1*B*; supplementary table S13, Supplementary Material online), and may play roles in lineage-specific traits. Functional annotation suggests that five of these *Bombus*-specific genes are associated with protein metabolism and transport (supplementary table S13, Supplementary Material online), potentially linked to the higher protein content of pollen collected by bumblebees than honeybees (Leonhardt and Blüthgen 2012) or the importance of proteins for insect diapause, which is a critical step in the bumblebee life cycle (Denlinger 2002; Colgan et al. 2011). Orthologous groups with the broadest species representation are functionally enriched for core biological processes such as protein transport, signal transduction (e.g., Wnt pathway), (de)ubiquitination, and cytoskeleton organization (supplementary table S14, Supplementary Material online). In contrast, those with sparse or lineage-restricted species representation are enriched for processes including olfactory and gustatory perception, amino acid biosynthesis, and oxidation–reduction (supplementary table S14, Supplementary Material online). On average, 465 species-specific genes (those without an ortholog in any other species) were identified in each bumblebee species (range 137–767) (supplementary table S15, Supplementary Material online), which may contribute to species-specific traits but whose functional roles remain to be explored.

Turnover analysis (gains and losses) of gene repertoires across the *Bombus* phylogeny (15 species, one per subgenus) using CAFE v3.0 (Han et al. 2013) identified expansions and contractions among 13,828 gene families and quantified variations in gene turnover rates across species (supplementary fig. S16, Supplementary Material online). After error correction, the overall rate of gene turnover in *Bombus* genomes is 0.0036/gene/My, similar to an analysis of 18 anopheline species and 25 drosophilids (supplementary table S16, Supplementary Material online) (Neafsey et al. 2015; Da Lage et al. 2019). However, these genus-specific gene turnover rates are 2–3 times higher than order-wide rates, which average 0.0011 (supplementary table S16, Supplementary Material online) (Thomas et al. 2020), possibly due to the denser sampling in genus-level studies that allow more events to be captured. Gene gain and loss events, along with the number of rapidly evolving gene families, are summarized for each species (supplementary table S17, Supplementary Material online), with a total of 3,797 rapidly changing gene families. The most dynamic gene families are enriched for processes including smell and taste perception, chitin metabolism, microtubule-based movement, and methylation (supplementary table S18, Supplementary Material online). Complementary analysis using three measures of gene copy number variation also identifies these processes as enriched among the most variable gene families, in contrast to the most stable that are involved in processes related to translation, adhesion, and transport (supplementary table S19, Supplementary Material online). In terms of protein domain copy number evolution, the most highly variable genes are those with protein–protein interaction mediating F-box domains, putatively DNA-binding SAP motifs, and phosphate-transferring guanylate kinases (supplementary table S20, Supplementary Material online).

### Stable Intron–Exon Structures with Abundant Stop-Codon Readthrough

Protein-coding potential analysis using *B. terrestris* as the reference species identified 851 candidate readthrough stop codons (supplementary fig. S17 and table S21, Supplementary Material online), that is, where translation likely continues through stop codons to produce extended protein isoforms. Coding potential was assessed using PhyloCSF (Lin et al. 2011) on whole-genome alignments of all 19 bumblebees and four honeybees. The false discovery rate was estimated using enrichment for the TGA-C stop-codon context, which is favored in readthrough genes, to infer that no more than 30% of the 200 highest-scoring candidates are false positives, and that at least 306 of our 851 candidates undergo functional readthrough. Although readthrough is rare beyond Pancrustacea, hundreds of *Drosophila* and *Anopheles* genes undergo readthrough (Jungreis et al. 2011; Dunn et al. 2013; Jungreis et al. 2016; Rajput et al. 2019) and our whole-genome-alignment-based results support the prediction (Jungreis et al. 2011) that insect species have abundant stop-codon readthrough. In contrast, intron–exon boundaries within bumblebee genes are relatively stable. Examining evolutionary histories of intron gains and losses revealed few changes, representing only 3–4% of ancestral intron sites, with more gains than losses (supplementary fig. S18 and table S22, Supplementary Material online), unlike drosophilids and anophelines where losses dominate (Neafsey et al. 2015), suggesting that bumblebee gene structure has remained relatively stable over the 34 My since their last common ancestor.

### Divergence and Selective Constraints of Protein-Coding Genes

**Fig. 3.**
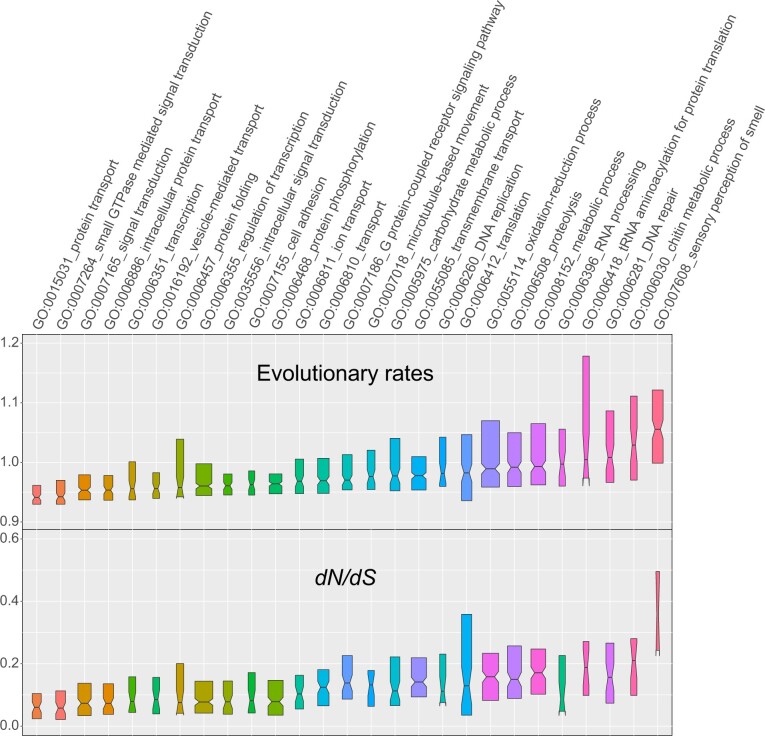
Molecular evolution of protein-coding genes in terms of evolutionary rate and d*N*/d*S* ratio. GO Biological Process terms are sorted by evolutionary rate from the most conservative (left) to the most dynamic (right) and colored from the highest values (red) to the median value (blue) to the lowest values (orange). Evolutionary rate refers to amino acid sequence divergence measured as the mean of normalized interspecies ortholog protein sequence identities. d*N*/d*S* refers to the ratio of the number of nonsynonymous substitutions per nonsynonymous site to the number of synonymous substitutions per synonymous site. Notched boxes show medians of orthologous group values with the limits of the upper and lower quartiles, and box widths are proportional to the number of orthologous groups in each category.

Bumblebee genes with elevated sequence divergence and/or relaxed constraints include processes related to smell perception, chitin metabolism, RNA processing, DNA repair, and oxidation–reduction (fig. 3). Measures of evolutionary rate (amino acid sequence divergence measured as the mean of normalized interspecies ortholog protein sequence identities) and selective constraint (d*N*/d*S*) showed similar trends among different functional categories of genes. Most genes are strongly constrained, with median estimates of d*N*/d*S* much lower than one. Assignment of GO terms and InterPro domains is usually biased toward slower-evolving, well-conserved genes (supplementary fig. S19, Supplementary Material online). Nevertheless, functional categories with the fastest-evolving genes are further supported and complemented by examining molecular function GO terms (supplementary fig. S20*A*, Supplementary Material online) and InterPro domains (supplementary fig. S20*B*, Supplementary Material online), which show elevated rates for odorant binding, olfactory receptor activity, chitin binding, oxidoreductase activity, serine-type endopeptidase activity, and olfactory receptor domains. GO term enrichment analysis of the slowest and fastest-evolving subsets of genes, bottom and top 20% respectively (supplementary fig. S21, Supplementary Material online), showed genes with the slowest evolutionary rates and the lowest d*N*/d*S* ratios were enriched for essential house-keeping biological processes and molecular functions (supplementary tables S23 and S24, Supplementary Material online). In contrast, genes with the fastest evolutionary rates were enriched for processes linked to polysaccharide biosynthesis, tRNA aminoacylation, drug binding, and RNA methyltransferase activity (supplementary table S23, Supplementary Material online). Genes with the highest d*N*/d*S* ratios were enriched for processes and functions including proteolysis, translation, ncRNA processing, and chitin metabolism (supplementary table S24, Supplementary Material online).

### Codon Usage Bias Driven by at Content

Analysis of codon usage bias showed no evidence for selection on optimal codons, in contrast to drosophilids but similar to anophelines (Vicario et al. 2007; Neafsey et al. 2015). Instead, codon usage bias in bumblebees seems to be driven mainly by AT content, consistent with previous reports in Hymenoptera (Behura and Severson 2012). Optimal codons were estimated in each species and correlation coefficients were computed between relative synonymous codon usage and effective number of codons per gene. All species have a similar preference and intensity of preference; for each amino acid, there was a consistently highly preferred codon and often a secondarily preferred one, all ending in A/T (supplementary fig. S22, Supplementary Material online). To test if codon usage could largely be explained by mutation bias, a linear model was used to predict Fop (frequency of optimal codon) from overall gene AT content and amino acid use. The model explained 99.2% of the Fop variation without the need to include the species origin of each gene. The AT content alone explained 81% of the variation (supplementary fig. S23, Supplementary Material online). Moreover, a strong correlation was observed between codon AT content and the correlation between relative synonymous codon usage and effective number of codons across all species (supplementary fig. S24, Supplementary Material online).

### Evolution of Genes Associated with Bumblebee Ecoethology

Many ecological and environmental factors—for example, shortage of food, pathogen emergence, pesticide exposure, and climate change—are contributing to the overall decline of bumblebees worldwide (Williams et al. 2009; Goulson et al. 2015; Cameron and Sadd 2020). To begin to explore the complement of genes likely to be involved in bumblebee interactions with their environment, we examined the evolution of gene families associated with their ecology and life histories. Sampling across the *Bombus* genus enabled the first survey of natural gene repertoire diversity of such families that are likely to be important for bumblebee adaptability and success.

#### Chemosensory Receptor Diversity

**Fig. 4.**
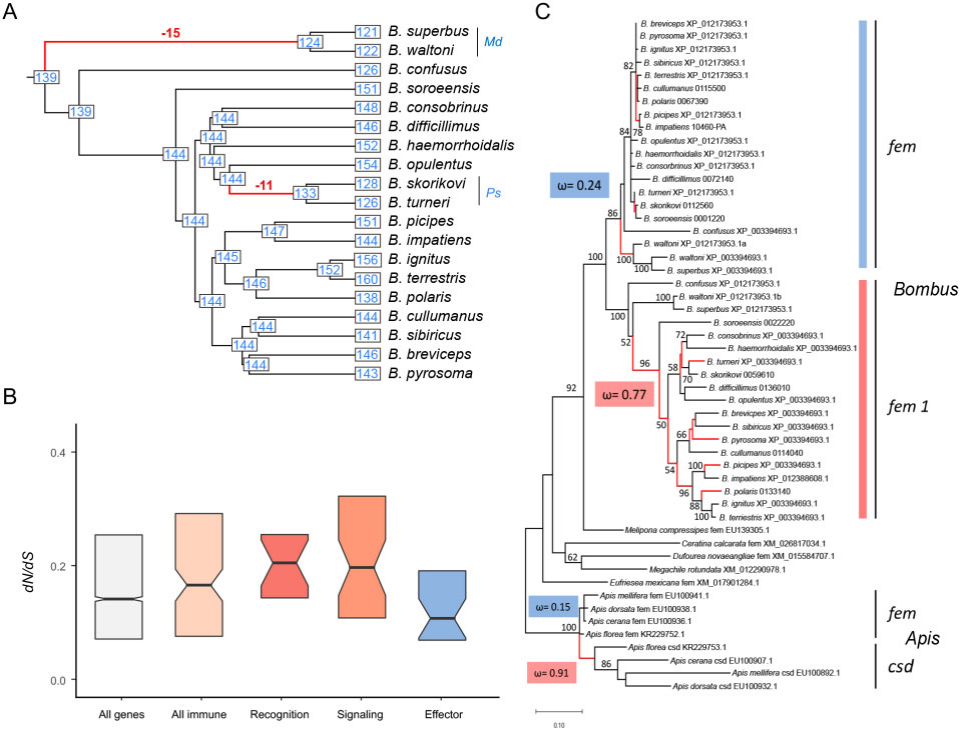
Evolution of genes associated with ecology and reproduction. (*A*). Observed gene counts and inferred ancestral gene counts of bumblebee ORs on an ultrametric phylogeny, highlighting two major gene loss events (the complete result is available in supplementary fig. S25, Supplementary Material online). (*B*). Boxplots showing d*N*/d*S* ratios for different categories of immune genes and all single-copy genes in bumblebee (All genes). Elevated d*N*/d*S* ratios among immune-related genes are driven by higher ratios for genes involved in recognition and signaling processes. Notched boxes show medians of orthologous group values with the limits of the upper and lower quartiles. (*C*). The evolutionary history of *fem* genes of bees including their paralogs *fem1* in bumblebees (*Bombus*) and *csd* in honeybees (*Apis*). Global nonsynonymous to synonymous rate ratio (*ω*) was calculated for *fem*_Bombus_ (reference, blue) and *fem1*_Bombus_ (test, red), including a branch-site testing framework with model fitting and Likelihood Ratio Tests, showing evidence for relaxation of selection in *fem1*_Bombus_ (*P* < 0.001, LR = 36.34). Spurious actions of diversifying selection on branches predominantly found in *fem1*_Bombus_ are marked in red. For comparison, *ω* for *fem* and *csd* in *Apis* is given, known as striking example of neofunctionalization.

Chemosensation plays a critical role in locating food and nests, communicating with nestmates, and identifying other environmental cues (Ayasse and Jarau 2014). A search of the three major chemosensory receptor gene families—odorant receptors (ORs), gustatory receptors (GRs), and ionotropic receptors (IRs)—in the sequenced bumblebee genomes identified 3,228 genes, with mean (minimum–maximum) counts of 150 (133–165) ORs, 18 (13–22) GRs, and 22 (21–22) IRs (supplementary table S25, Supplementary Material online). Only complete genes were used for gene gain and loss analysis. Despite the similarities in total OR gene counts, examples of gene gain/loss were observed in specific lineages. There was a net loss of 15 ORs in the common ancestor of the subgenus *Mendacibombus* (*Md*) (fig. 4*A*; supplementary fig. S25, Supplementary Material online). Species in *Mendacibombus* mainly inhabit high mountains including the Qinghai–Tibetan plateau, with relatively low floral diversity (Williams et al. 2018), which may be linked to OR loss in this subgenus. A net loss of 11 ORs was observed in the common ancestor of subgenus *Psithyrus* (*Ps*) (fig. 4*A*; supplementary fig. S25, Supplementary Material online). For ORs shared across bumblebees, seven showed evidence of positive selection in a subset of species, including putative pheromone receptors (supplementary table S26, Supplementary Material online). Compared with ORs, GRs and IRs have much lower and more stable gene counts (supplementary fig. S25, Supplementary Material online). However, despite overall conservation of gene number and widespread evidence for purifying selection, there is evidence that some GR and IR genes experienced positive selection in a subset of species, including receptors putatively involved in sensing fructose and temperature (supplementary table S26, Supplementary Material online).

#### Detoxification Capacity

Detoxification genes are used to neutralize xenobiotics, such as toxic plant secondary metabolites and pesticides. Repertoires of carboxyl/cholinesterases, cytochrome P450 monooxygenases, and glutathione S-transferases in the 17 genomes are much smaller than in drosophilids and anophelines (supplementary table S27, Supplementary Material online), indicating a genus-wide deficit of this gene category, previously observed in two bumblebees (Sadd et al. 2015). There are 88 detoxification genes on average in bumblebees, with little variation across species (supplementary table S27, Supplementary Material online). Despite overall conservation of gene number and widespread evidence for purifying selection (mean d*N*/d*S* is 0.26), a total of 19 detoxification genes, including carboxyl/cholinesterases, cytochrome P450 monooxygenases, and glutathione S-transferases, showed evidence of positive diversifying selection in a subset of species (supplementary table S28, Supplementary Material online).

#### Immune Defense

Immune genes are involved in recognition of and defense against pathogens. Similar to detoxification genes, counts in the 17 sequenced genomes are much lower than in drosophilids and anophelines (supplementary table S29, Supplementary Material online), showing that the previously noted paucity in two bumblebees (Barribeau et al. 2015; Sadd et al. 2015) extends to the whole genus. Bumblebee genomes contain components of all major immune pathways described in insects, and gene counts are fairly conserved across species (supplementary table S29, Supplementary Material online). For example, all species have two genes encoding Gram-negative bacteria-binding proteins, whereas peptidoglycan-recognition proteins are more variable with between four and six gene copies. Comparing d*N*/d*S* ratios between immune genes and all single-copy orthologous genes in bumblebees showed that immune genes exhibit slightly higher d*N*/d*S* ratios (*P* = 0.04, Wilcoxon rank sum test), and among immune genes, recognition and signaling genes have higher d*N*/d*S* ratios than effector genes (fig. 4*B*). In addition, despite widespread evidence for purifying selection, a total of 52 immune genes showed evidence of positive selection in a subset of bumblebee species (supplementary table S30, Supplementary Material online).

#### Genes Involved in High-Elevation Adaptation

*Bombus superbus*, *B. waltoni*, *B. difficillimus*, and *B. skorikovi* are four species collected at elevations >4,000 m that represent three subgenera (fig. 1). No genes show signatures of positive selection in all high-elevation species but none of the low-elevation species. However, six genes show evidence of positive selection in species representing two of the three high-elevation subgenera, but none of the low-elevation species (supplementary table S31, Supplementary Material online). One encodes CPAMD8, which is involved in eye development (Cheong et al. 2016). As bumblebees detect flowers visually (Meyer-Rochow 2019), signatures of selection might be related to fine tuning eye development for optimal foraging in high-altitude light conditions. Three genes encode a histone deacetylase, synaptotagmin-12, and a heterogeneous nuclear ribonucleoprotein, which are involved in maintaining muscle integrity and keeping “flight state,” which is critical for undertaking long-distance food searching (Liu et al. 2001; Manjila et al. 2019; Pigna et al. 2019). Two genes encode a sodium-coupled monocarboxylate transporter and a glycosyltransferase family protein, which are believed to be involved in metabolic adaptation to hypoxia (Véga et al. 2006; Shirato et al. 2010) (supplementary table S31, Supplementary Material online).

#### Sex Determination

Evolutionary analysis of sex-determination genes in bumblebees and related species indicated that all bumblebee genomes share a duplicated copy of *feminizer* (*fem*), named *fem 1* (fig. 4*C*). Compared with *fem*, *fem 1* shows a higher level of divergence among bumblebees (*fem_Bombus_* d*N*/d*S* = 0.24; *fem 1_Bombus_* d*N*/d*S* = 0.77; fig. 4*C*). These ratios are close to the range observed for *Apis*, in which *fem* has evolved under purifying selection and the paralogous gene *complementary sex determiner* (*csd*) has evolved by neofunctionalization (fig. 4*C*) (Hasselmann et al. 2008). A hypothesis branch-site testing framework (RELAX; Wertheim et al. 2015) identifies evidence for relaxation of selection in *fem 1_Bombus_* compared with *fem_Bombus_* (*P* < 0.001, LR = 36.34). Moreover, the spurious action of diversifying selection on branches was predominantly found in *fem 1_Bombus_* (fig. 4*C*). A mixed effect model of evolution (Murrell et al. 2012) was applied to identify individual sites that were subject to episodic diversifying selection, and at least 15 sites (*P* < 0.05) were found to be under positive selection, with some being located in known motifs (supplementary fig. S26, Supplementary Material online). The results of these selection analyses suggest that both *fem* and *fem 1* contribute to the bumblebee sex-determination pathway. For the *transformer 2* (*tra-2*) gene, consistent amino acid changes between *Bombus* and *Apis* were found within the RNA recognition domain (supplementary fig. S27, Supplementary Material online), supporting a previous hypothesis of a regulatory modification between honeybees and bumblebees (Biewer et al. 2015).

## Discussion

Comparative analysis of multiple genomes in a phylogenetic framework substantially improves the precision and sensitivity of evolutionary inference and provides robust results identifying stable and dynamic features. In this study, we performed comparative analyses of genome structures and contents, as well as global and family-targeted gene evolutionary dynamics across the phylogeny of the genus *Bombus*, using 17 annotated de novo assemblies and two previously published genomes.

Many attributes of bumblebee genomes are highly conserved across species. For example, overall genome size and genome structure, the number of protein-coding genes and noncoding RNAs, gene intron–exon structures, and the pattern of codon usage are all very similar across these 19 genomes. However, other aspects of genome biology are dynamically evolving. TEs are a major contributor to genome size variation (fig. 1) as well as a potential source of coding and regulatory sequences (supplementary tables S10–S12, Supplementary Material online). Differential gene gain and loss also contribute to gene content variation across bumblebees and lead to lineage-specific gene repertoires (fig. 4*A*; supplementary fig. S25 and table S17, Supplementary Material online). Finally, for genes shared by all species, the action of positive selection is different across species (supplementary tables S26, S28, S30, and S31, Supplementary Material online), which can lead to gene functional divergence possibly reflecting key ecoethological differences.

An exception to the otherwise overall conserved genome structure is the set of species in the subgenus *Psithyrus*. These bumblebees exhibit social parasitism; they do not have a worker caste, and it is not necessary for them to forage for nectar and pollen to provision developing larvae (Lhomme and Hines 2019). Originally, this subgenus was argued to be a separate genus due to distinct behavior and higher chromosome number; however, subsequent phylogenetic analysis placed *Psithyrus* within the genus *Bombus* (Cameron et al. 2007; Williams et al. 2008). Here, based on a much larger genomic data set, we confirm that species in the subgenus *Psithyrus* (labeled *Ps* in purple in fig. 1*A*) form a monophyletic group within the genus *Bombus*. In addition, we show that, although *Psithyrus* species have an increased chromosome number, their genome sizes are within the range of those of the other bumblebees (fig. 1*A*), and their 25 chromosomes reflect a mix of fission, fusion, and retention of the 18 ancestral bumblebee chromosomes (fig. 2; supplementary fig. S10, Supplementary Material online). Chromosome rearrangements (e.g., fissions, fusions, and inversions) have been posited to play roles in speciation (Ayala and Coluzzi 2005) and thus may explain the diversification and social parasitic behavior of *Psithyrus*. In addition to genome structure variation, we identified a net loss of 11 OR genes in the common ancestor of *Psithyrus* species (fig. 4), which could be a cause or consequence of their socially parasitic behavior.

Bumblebee species exhibit different food preferences (Goulson and Darvill 2004; Sikora and Kelm 2012; Somme et al. 2015), but the genetic basis underlying such variation is unknown. Like in other insects, smell and taste are used to distinguish different food sources (Kunze and Gumbert 2001; Ruedenauer et al. 2015). In this study, we found out that genes involved in olfactory and gustatory perception are among the fastest-evolving gene categories, both in copy number variation and in sequence divergence (fig. 3; supplementary fig. S20 and tables S18 and S19, Supplementary Material online). Therefore, the dynamic evolution of genes involved in olfactory and gustatory perception likely relates to different food preferences, improved understanding of which could inform the use of new species in agricultural settings. The importance of chemical perception for social Hymenoptera in mating (Ayasse et al. 2001), determination of reproductive status (Monnin 2006), and recognition of kin (Page and Breed 1987) could also contribute to rapid evolution of genes in this category.

Bumblebees exhibit rich morphology differences across species including mandible, labrum, tibia, and basitarsus structures, as well as patterns of keels, ridges, and grooves formed by the cuticle (Williams 1994), and they show species-specific responses to insecticides (Baron et al. 2017). Chitin is a major component of the insect cuticle and peritrophic matrix, and chitin metabolic processes are related to morphogenesis, resistance to insecticides, and the tolerance of toxins in food (Barbehenn 2001; Merzendorfer and Zimoch 2003; Zhu et al. 2016; Erlandson et al. 2019). Genes related to chitin metabolism are also among the fastest-evolving functional categories in bumblebees, both in copy number variation and in sequence divergence (fig. 3; supplementary fig. S20 and tables S18 and S19, Supplementary Material online). These variable patterns of chitin-related gene evolution potentially underlie observed differences in exoskeleton morphological characters and insecticide resistance, but not color pattern variation, which is determined primarily by hair pigmentation (Tian et al. 2019). Across bumblebee genomes some of the fastest-evolving genes are also related to processes including protein glycosylation, methylation, proteolysis, and tRNA aminoacylation for protein translation (fig. 3; supplementary fig. S20 and tables S18 and S19, Supplementary Material online). Protein glycosylation is involved in multiple physiological processes including growth, development, circadian rhythms, immunity, and fertility (Walski et al. 2017). tRNA aminoacylation for protein translation process is involved in response to the changing environment (Pan 2013).

Some genes that are not among the fastest-evolving categories—for example, immune and detoxification genes, which are involved in the interaction of bumblebees with external environments—show differential patterns of positive selection in subsets of species, which can lead to gene functional divergence. The positive diversifying selection found in some detoxification genes of a subset of species could also contribute to species differences in response to insecticide exposure (Baron et al. 2017). Likewise, evidence of positive selection among immune genes in some species but not others (supplementary table S30, Supplementary Material online) suggests that interactions with pathogens and parasites have been important during the evolutionary history of these species, where selective pressures encountered in different ecological niches may vary among species. These differences could affect susceptibility to emerging and re-emerging infectious diseases and explain observed species-specific differences in contemporary pathogen prevalence (Cameron et al. 2016; Cameron and Sadd 2020). Taken together, identification of the fastest-evolving genes and those showing patterns of differential positive selection reveals substantial genetic variation across bumblebee genera. Future experimental investigations will be required to determine how the identified genetic variation is linked to specific differences in traits such as food preference, morphogenesis, insecticide and pathogen resistance, and the response to changing environments.

In addition to our discoveries regarding protein-coding genes, we found that TE-related sequences likely contribute to the variation of coding and regulatory repertoires (fig. 1; supplementary tables S10–S12, Supplementary Material online). Compared with non-*Mendacibombus* bumblebees, *Mendacibombus* species have smaller genomes (fig. 1) and relatively narrow geographical distributions (Williams et al. 2016). Considering TEs are the major determinant of genome size difference, with evidence that they were potentially domesticated in bumblebee genomes (supplementary fig. S15 and tables S10–S12, Supplementary Material online), TEs may be implicated in the dispersal of non-*Mendacibombus* species across the globe, as they have been in other taxa (Casacuberta and González 2013; Baduel et al. 2019; Schrader and Schmitz 2019).

More recent range expansions or contractions are driven, at least in part, by global climate change. In response to a warming climate, there is evidence that the ranges of some bumblebees are being pushed northward or to higher elevations (Ploquin et al. 2013; Kerr et al. 2015; Biella et al. 2017; Soroye et al. 2020). The sequenced genomes of species collected at high-elevation sites (>4,000 m) and others collected at low elevations (<2,000 m) (fig. 1) represent high-quality genomic resources for investigating genes involved in high-elevation adaptation. For example, population-genomics studies facilitated by the *A. cerana* reference assembly identified candidate high-altitude adaptation loci in that species (Montero-Mendieta et al. 2019) and the *B. impatiens* genome was used to identify climate adaptation across latitude and altitude in two montane North American bumblebee species (Jackson et al. 2020). We identified genes showing signs of positive selection in at least two subgenera of high-elevation species but not in any of the low-elevation species (supplementary table S31, Supplementary Material online). These include genes putatively involved in eye development, flight muscle integrity, and metabolism, highlighting the importance of successful food searching in high-elevation habitats where food is scarce. Signatures of positive selection in neuro-muscular genes mirror findings from the population genomic study on the two North American montane species (Jackson et al. 2020). Beyond specific genes, comparing high- and low-elevation species showed a consistent pattern of faster genome-wide evolutionary rates in those occupying lower elevations (Lin et al. 2019). Exploring these trends and genes further and identifying additional genomic features linked to life at high altitudes will help to understand differential successes of bumblebee species in a changing world.

## Conclusions

We have produced highly complete and accurate genome assemblies of 17 bumblebee species, including representatives from all 15 *Bombus* subgenera. Our genus-wide comparative analysis of bumblebee genomes revealed how genome structures, genome contents, and gene evolutionary dynamics vary across bumblebees and identified genetic variations that may underlie species trait differences in foraging, diet and metabolism, morphology and insecticide resistance, immunity and detoxification, as well as adaptations for life at high altitudes. Our work provides genomic resources that capture genetic and phenotypic variation, which should advance our understanding of bumblebee success and help identify potential threats. These resources form a foundation for future research, including resequencing and population-genomics studies for functional gene positioning and cloning, which will inform the use of bumblebees in agriculture, as well as the design of strategies to prevent the decline of these important pollinators.

## Materials and Methods

For detailed methods, please see supplementary Materials and Methods, Supplementary Material online. Genomic DNA purified from one single haploid drone of each species was used to generate one “fragment” library to produce overlapping paired-end shotgun reads (2× 250 bp). Genomic DNA purified from three to five individuals of each species was used to generate four “jumping” libraries (insert sizes: 4, 6, 8, and 10 kb, respectively). For each species, the 250-bp overlapping paired-end shotgun reads from the fragment library were assembled using the software DISCOVAR de novo (Love et al. 2016) to produce contiguous sequences (contigs). Shotgun reads from jumping libraries were used to scaffold the contigs using the software BESST (Sahlin et al. 2014). Hi-C sequencing libraries were generated as described previously (Belton et al. 2012; Lin et al. 2018), and the 3D-DNA pipeline (Dudchenko et al. 2017) was applied to assemble the scaffold sequences to chromosome level. The contiguity of the genome assemblies was compared with other genomic features (supplementary fig. S28, Supplementary Material online). The completeness of the genome assemblies was evaluated using BUSCO v3 (Waterhouse et al. 2018). TEs were de novo identified by LTRharvest (Ellinghaus et al. 2008), MGEScan-non-LTR (Rho and Tang 2009), and RepeatScout (Price et al. 2005). Protein-coding genes were annotated with the MAKER pipeline (Cantarel et al. 2008), based on ab initio gene predictions, transcript evidence (supplementary table S33, Supplementary Material online), and homologous protein evidence, and assessed with BUSCO v3 (Waterhouse et al. 2018). Orthologous groups were delineated by using the OrthoDB software (Kriventseva et al. 2015), which was also used to compute evolutionary rates. IQ-TREE (Minh, Schmidt, et al. 2020) and ASTRAL (Zhang et al. 2018) were employed for phylogeny analysis. Testing for introgression employed the Δ statistic (Huson et al. 2005), which follows the same logic as the ABBA-BABA site patterns used to calculate *D*-statistics, but uses tree topologies instead of alignment sites. MCScanX (Wang et al. 2012) was used to perform gene synteny analysis. Global gene family evolution was estimated with CAFE v3.0 (Han et al. 2013). PAML 4 (Yang 2007) was used to detect positive selection and calculate d*N*/d*S* ratios across alignments of single-copy orthologs. This was followed by aBSREL (Smith et al. 2015) analysis for gene families of special interest (i.e., chemosensory genes, detoxification genes, immune genes, sex-determination genes, and piRNA pathway genes) and for genes identified in the initial PAML scans as potentially showing signatures of adaptation to high elevation. Sex-determination genes were additionally analyzed using RELAX (Wertheim et al. 2015) and mixed effect model of evolution (Murrell et al. 2012). Intron history analyses were performed using Malin (Csűros 2008). Whole-genome alignments were produced using Cactus (Paten et al. 2011). PhyloCSF (Lin et al. 2011) was used to study stop-codon readthrough. Codon usage bias was analyzed as described previously (Vicario et al. 2007).

## Supplementary Material

Supplementary data are available at *Molecular Biology and Evolution* online.

## Supplementary Material

msaa240_Supplementary_Data
