# Erratum to: Genus-wide characterization of bumblebee genomes provides insights into their evolution and variation in ecological and behavioral traits

**DOI:** 10.1093/molbev/msab100

**Published:** 2021-05-20

**Authors:** Cheng Sun, Jiaxing Huang, Yun Wang, Xiaomeng Zhao, Long Su, Gregg W C Thomas, Mengya Zhao, Xingtan Zhang, Irwin Jungreis, Manolis Kellis, Saverio Vicario, Igor V Sharakhov, Semen M Bondarenko, Martin Hasselmann, Chang N Kim, Benedict Paten, Luca Penso-Dolfin, Li Wang, Yuxiao Chang, Qiang Gao, Ling Ma, Lina Ma, Zhang Zhang, Hongbo Zhang, Huahao Zhang, Livio Ruzzante, Hugh M Robertson, Yihui Zhu, Yanjie Liu, Huipeng Yang, Lele Ding, Quangui Wang, Dongna Ma, Weilin Xu, Cheng Liang, Michael W Itgen, Lauren Mee, Gang Cao, Ze Zhang, Ben M Sadd, Matthew W Hahn, Sarah Schaack, Seth M Barribeau, Paul H Williams, Robert M Waterhouse, Rachel Lockridge Mueller

*Molecular Biology and Evolution*, Volume 38, Issue 2, February 2021, Pages 486–501, https://doi.org/10.1093/molbev/msaa240

In the originally published version of this manuscript, there were two errors.

The incorrect Associate Editor was listed. The correct Associate Editor is Fuwen Wei. This error has been corrected online.

The Supplementary Materials section included two incorrect files, which have now been removed. The correct Supplementary Tables were originally missing and have now been uploaded.

