# Supplementary material for: Genus-Wide Characterization of Bumblebee Genomes Provides Insights into Their Evolution and Variation in Ecological and Behavioral Traits
Source: Mol Biol Evol. 2020 Sep 18;38(2):486–501. doi: 10.1093/molbev/msaa240 (PMC7826183; doi:10.1093/molbev/msaa240)

Genus-wide characterization of bumblebee genomes provides insights into their evolution and variation in ecological and behavioral traits

Supplementary figures

Figure S1. Collection sites of the 17 bumblebee species for genome sequencing....................2
Figure S2. BUSCO assessment of genome assembly completeness..2
Figure S3. Correlation between gene count and genome assembly contiguity. 3
Figure S4. BUSCO assessment of genome annotation quality...3
Figure S5. Comparison of maximum likelihood concatenated and quartet-based (ASTRAL) species topologies. 4
Figure S6. Gene tree support for *Bombus* nodes on the IQ-TREE species tree..........................5
Figure S7. Branch length and concordance factor are highly correlated in both the concatenated (A) and ASTRAL (B) trees. 6
Figure S8. Delta (Δ) distributions..6
Figure S9. The delta (Δ) values, z-scores, and p-values for each lineage in the concatenated and ASTRAL trees. 7
Figure S10. Hi-C assisted genome assembly and the origin of *B. turneri* chromosomes. 8
Figure S11. Ancestral genome size of bumblebees inferred by Mesquite 3.51. 9
Figure S12. Simple sequence repeat content versus genome size differences..........................9
Figure S13. Transposable element counts. 9
Figure S14. TE proliferation history in *Mendacibombus* species (*B. superbus* and *B. waltoni*) and in two representative non-*Mendacibombus* species (*B. terrestris* and *B. turneri*)...........10
Figure S15. Example of a TE contributing to the coding sequences of a bumblebee gene...11
Figure S16. Gene turnover rates across the *Bombus* phylogeny. 12
Figure S17. Example of stop codon readthrough in *B. terrestris*. 13
Figure S18. Tree with nodes labeled for Malin analysis. 14
Figure S19. Functional annotation bias towards conserved genes. 14
Figure S20. Molecular evolution of protein-coding genes. 15
Figure S21. Evolutionary rate and *dN/dS* ratio distributions. 16
Figure S22. Correlation coefficient between RSCU and ENC. 16
Figure S23. Correlation between gene AT content and the frequency of optimal codons.....17
Figure S24: Relationship between codon AT content and correlation shown in Figure S22. .17
Figure S25. Evolutionary histories of chemosensory genes in bumblebees. 18
Figure S26. Sex-determination genes fem and fem1. 19
Figure S27. Sex-determination gene tra2..19
Figure S28. Comparisons of assembly contiguity with other genomic features. 20

Figure S1. Collection sites of the 17 bumblebee species for genome sequencing.
Colored dots on the map show the sampling locations of all collected species.

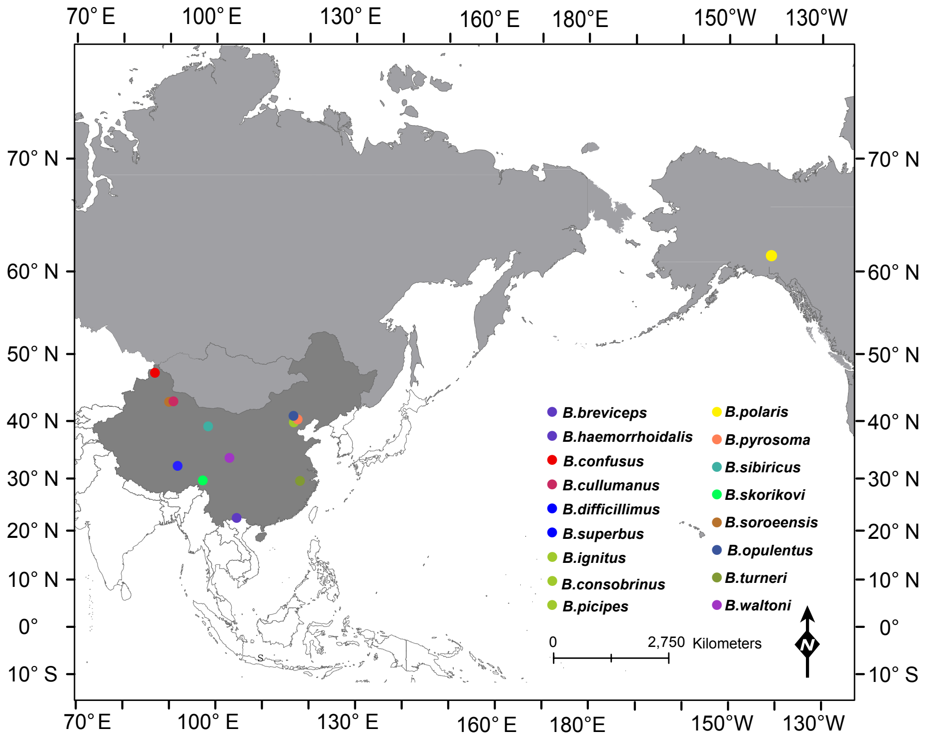

Figure S2. BUSCO assessment of genome assembly completeness. A set of 4,415 universal single-copy orthologs (lineage dataset: hymenoptera_odb9) was used to check for presence and completeness in each of the 17 genome assemblies.

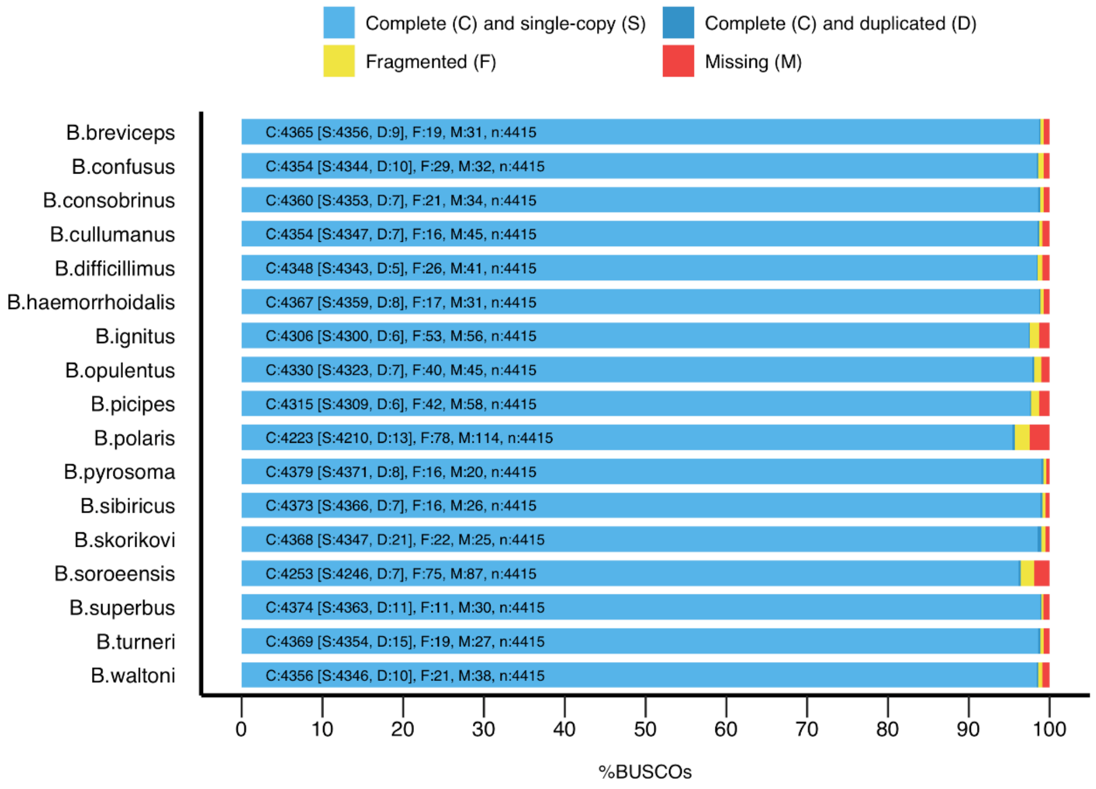

Figure S3. Correlation between gene count and genome assembly contiguity.
Pearson correlation analysis between gene count and genome assembly contiguity (scaffold N50) of the 17 newly produced bumblebee genome assemblies.

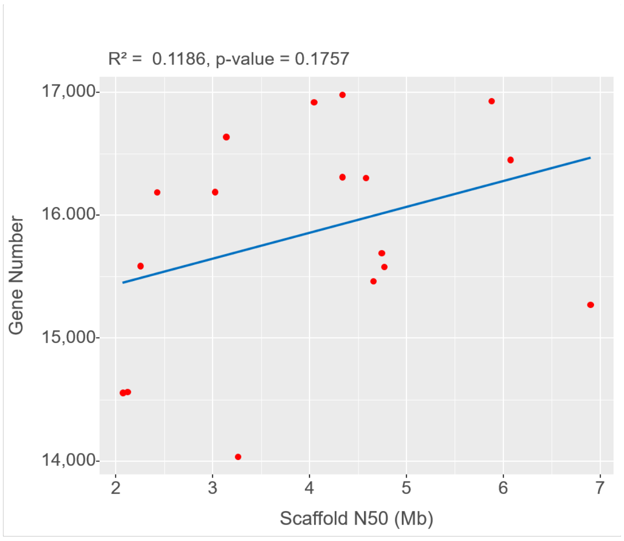

Figure S4. BUSCO assessment of genome annotation quality.
A set of 4,415 universal single-copy orthologs (lineage dataset: hymenoptera_odb9) was used to check for presence and completeness in each of the 17 predicted proteomes.

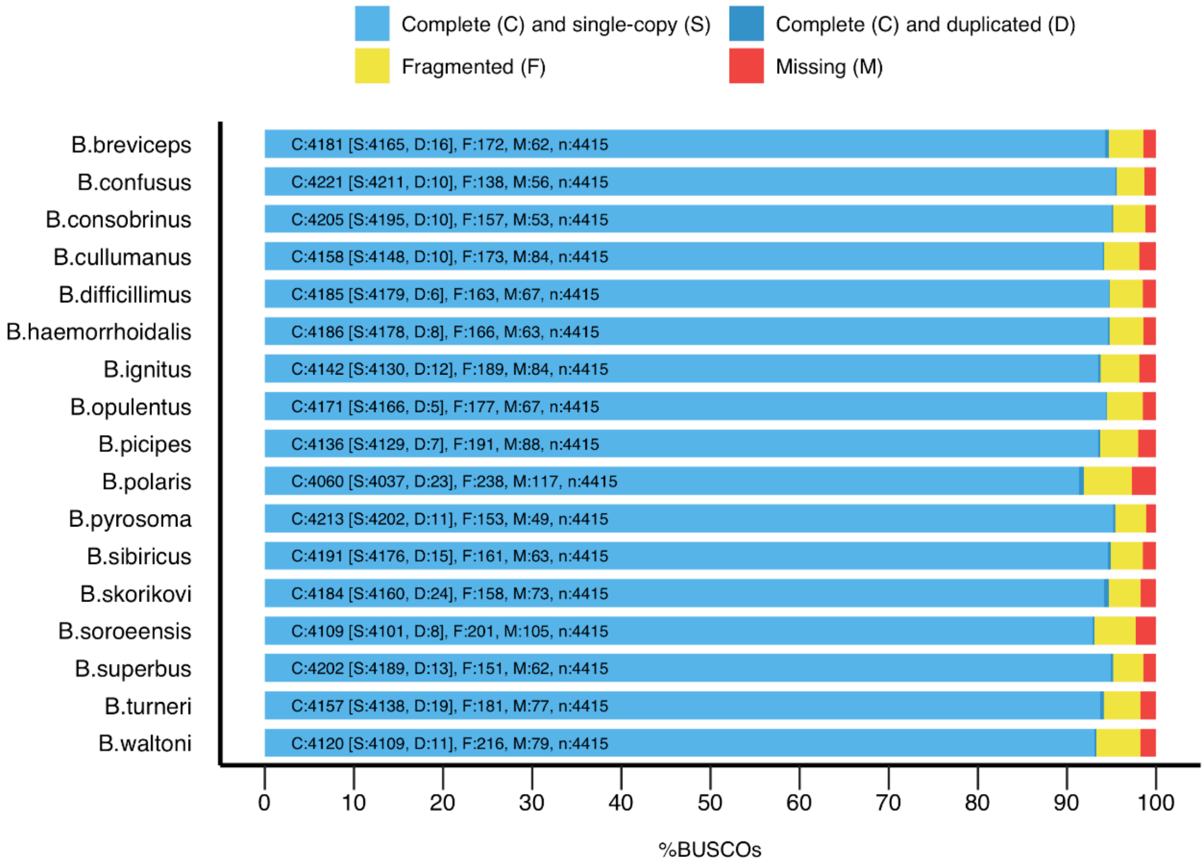

Figure S5. Comparison of maximum likelihood concatenated and quartet-based (ASTRAL) species topologies.

Branch lengths are unscaled. Numbers beside each node in the concatenated tree represent bootstrap values, while numbers beside each node in the ASTRAL tree indicate posterior probability. The only topological difference between the two trees is highlighted in the teal rectangle.

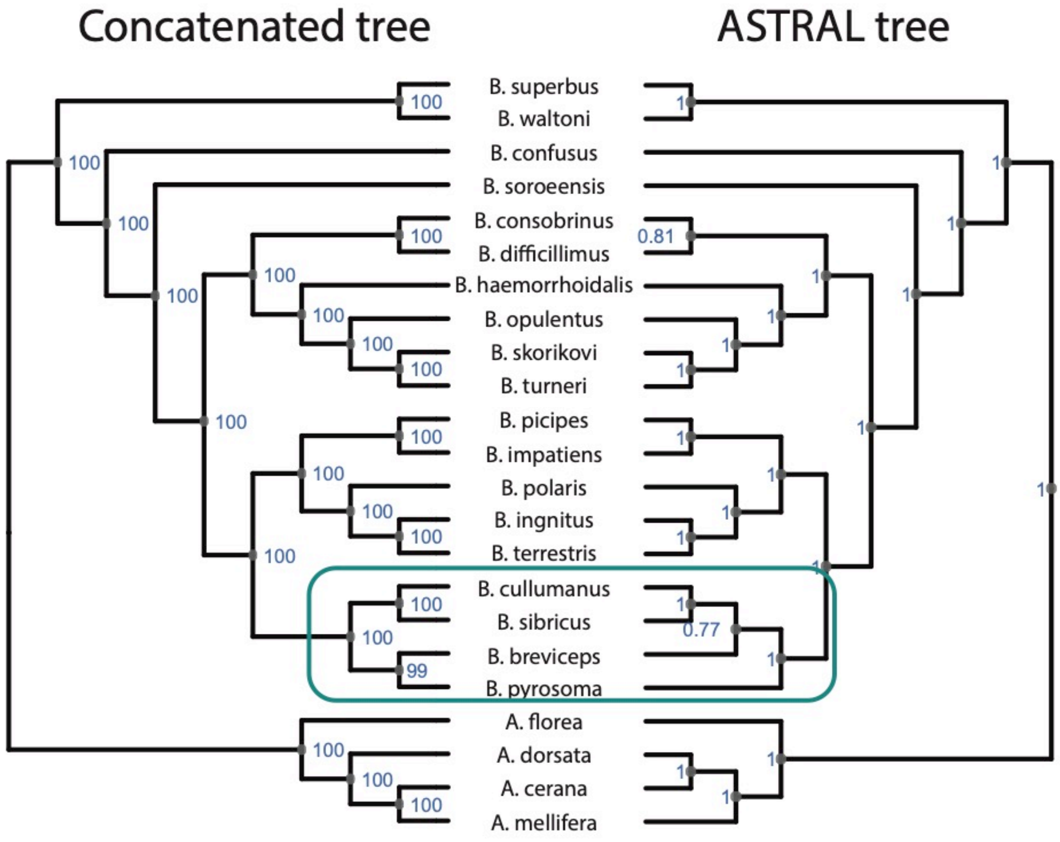

Figure S6. Gene tree support for *Bombus* nodes on the IQ-TREE species tree.
For the phylogeny shown in Figure 1A (here topology only): percentages of the numbers of gene trees out of a total of 2,657 trees (**A**) that support each node (blue/purple, low discordance; red/orange, high discordance). Percentages correspond to gene concordance factors (gCF) shown as labels on the phylogeny in Figure 1A, which reflect the percentage of gene trees that contain that node as defined by its descendant taxa. Support percentages on the species tree for the subsets of trees with (**B**) more than 60% of nodes with more than 60% bootstrap support and (**C**) more than 70% of nodes with more than 70% bootstrap support. (**D**) Counts of trees with more than X% of nodes with more than X% bootstrap support for cut offs from 10% to 90%.

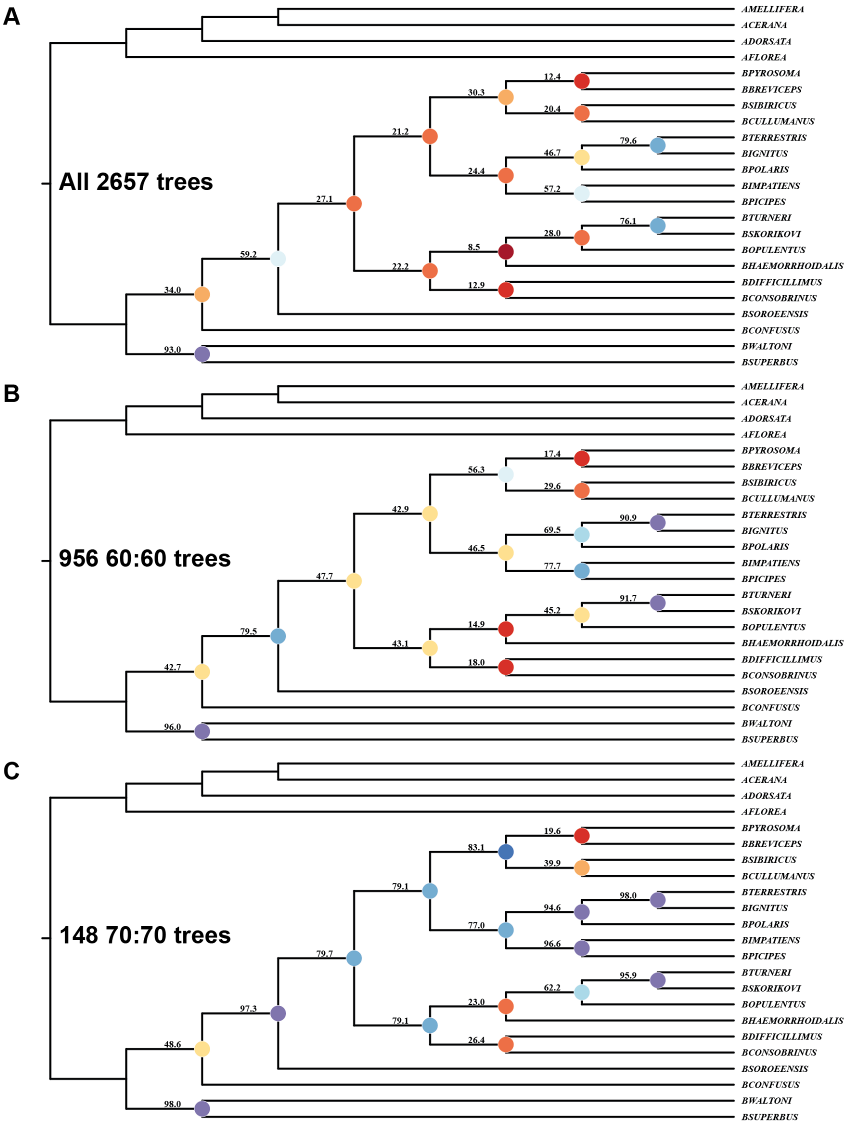

D

Proportion of nodes >: (columns); Bootstrap >: (rows)

Bootstrap >:	10	20	30	40	50	60	70	80	90
10	2657	2657	2657	2657	2657	2655	2611	2424	1890
20	2657	2657	2657	2657	2656	2616	2421	1927	1122
30	2657	2657	2657	2657	2639	2479	2038	1357	619
40	2657	2657	2656	2640	2532	2155	1508	855	300
50	2657	2657	2645	2541	2195	1588	950	413	93
60	2657	2650	2556	2211	1599	956	437	140	13
70	2655	2589	2241	1602	922	416	148	33	1
80	2634	2269	1562	856	377	109	29	2	0
90	2340	1356	628	221	69	18	3	0	0

Figure S7. Branch length and concordance factor are highly correlated in both the concatenated (A) and ASTRAL (B) trees.

The points each represent one internal node and the dashed line is the best-fit line of a linear regression. Branch lengths in the maximum likelihood concatenated tree represent relative numbers of substitutions while branch lengths in the ASTRAL tree represent coalescent units.

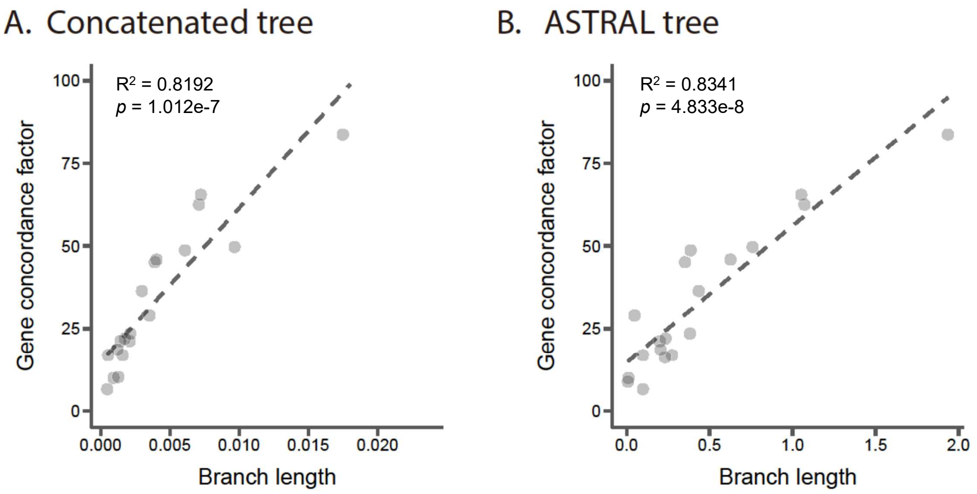

Figure S8. Delta (Δ) distributions.

(**A**). Distribution of Δ calculated from the concatenated maximum likelihood phylogeny based on 1,000 bootstrap replicates of gene tree sampling. (**B**). Distribution of Δ calculated from the ASTRAL phylogeny based on 1,000 bootstrap replicates of gene tree sampling. (**C**). Observed values of Δ for lineages with gCF < 95% when compared to the concatenated maximum likelihood phylogeny. (**D**). Observed values of Δ for lineages with gCF < 95% when compared to the ASTRAL phylogeny.

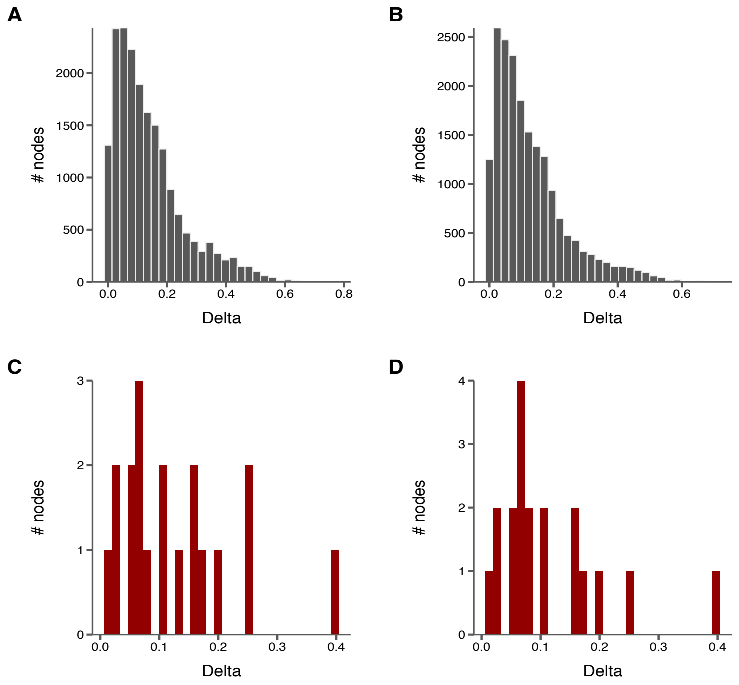

Figure S9. The delta (Δ) values, z-scores, and p-values for each lineage in the concatenated and ASTRAL trees.

Node labels in blue are of the following format: delta values | z-scores | p-values. No lineages in either species tree have significantly high values of Δ.

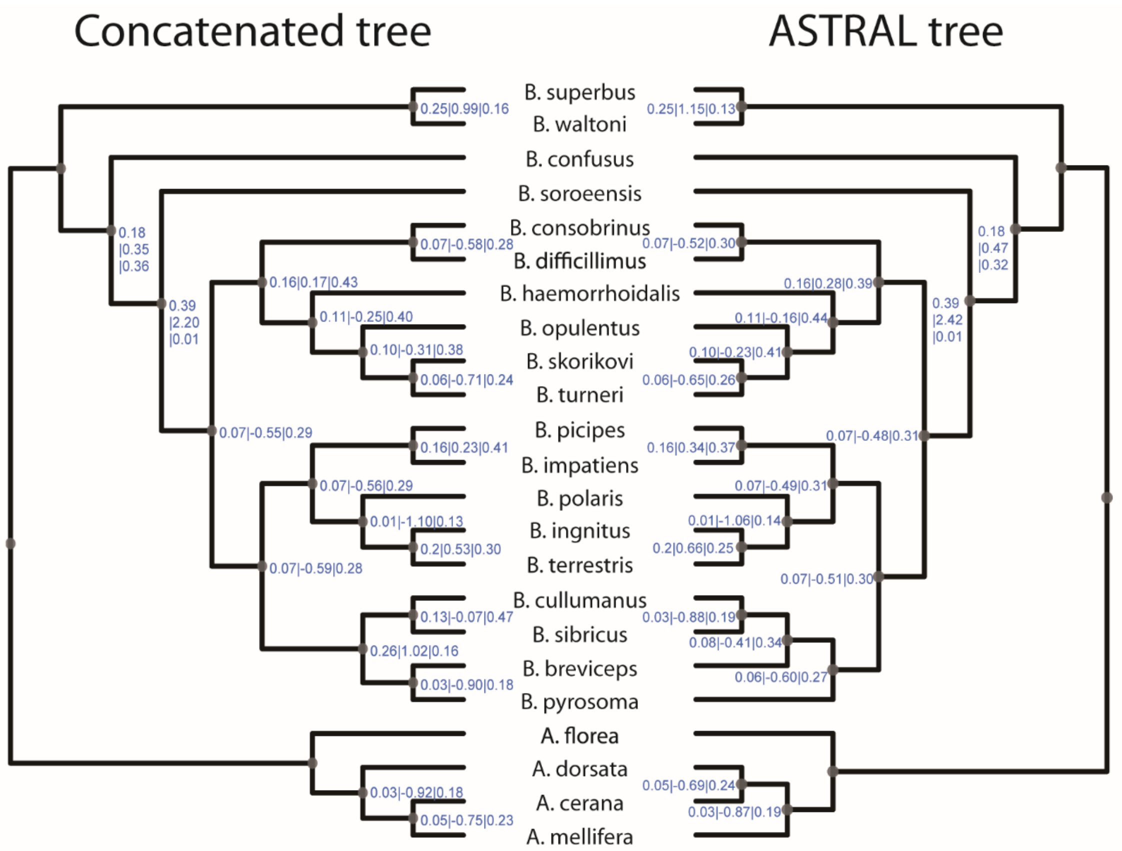

Figure S10. Hi-C assisted genome assembly and the origin of *B. turneri* chromosomes. Hi-C contact heatmaps for *B. breviceps* (**A**) and *B. ignitus* (**B**). (**C**) Macrosynteny across *B. breviceps*, *B. turneri* and *B. ignitus* to show the origin of *B. turneri* chromosomes. (**D**). Macrosynteny between *B. turneri* and subgenus *Bombus* species (*B. ignitus* and *B. terrestris*) to show the origin of *B. turneri* chromosomes.

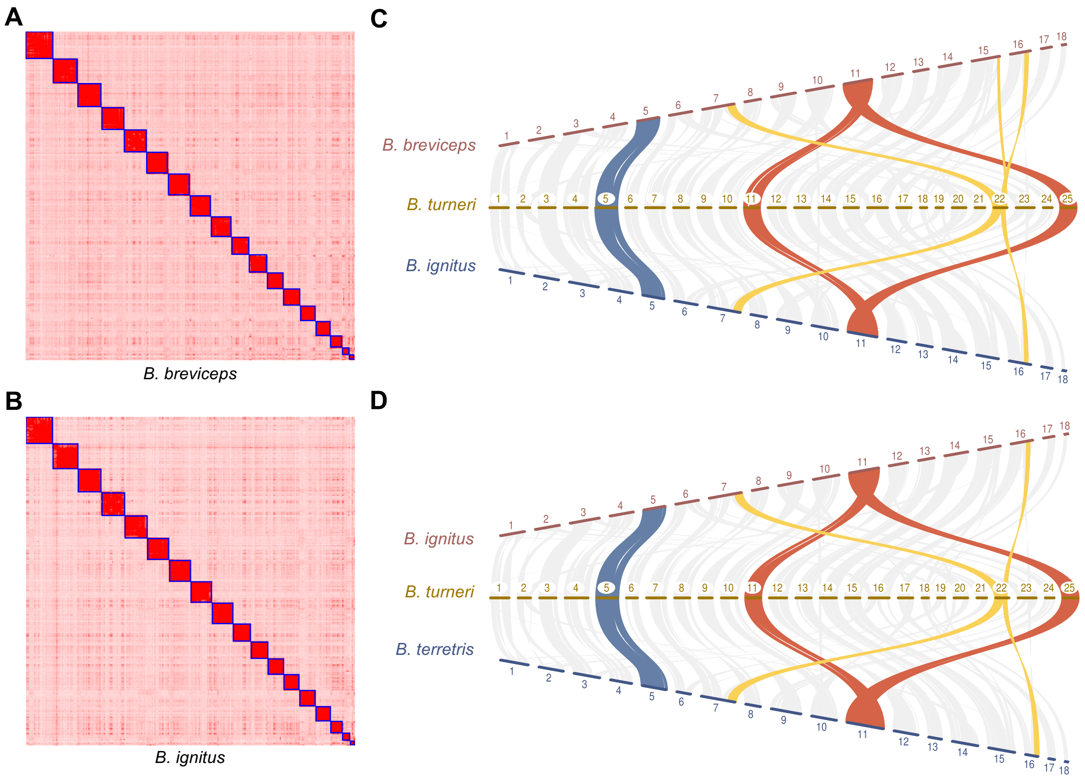

Figure S11. Ancestral genome size of bumblebees inferred by Mesquite 3.51.

Numbers on nodes indicate the inferred ancestral genome sizes (in Mb). The genome assembly sizes of each species were shown in brackets following their species names (in Mb).

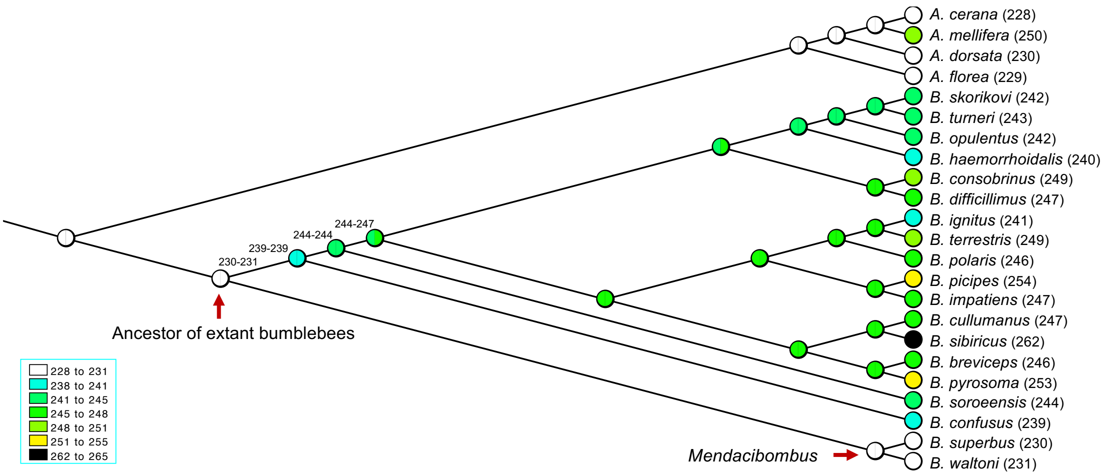

Figure S12. Simple sequence repeat content versus genome size differences.

Pearson correlation analysis between differences in simple sequence repeat content relative to that of *B. superbus* and differences in genome size (relative to that of *B. superbus*).

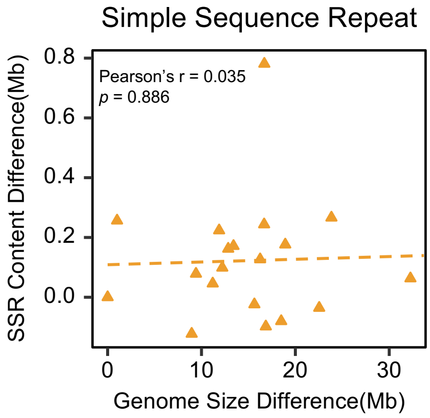

Figure S13. Transposable element counts.

The number of TEs in each non-*Mendacibombus* species that proliferated after the divergence of their host species from *Mendacibombus* species.

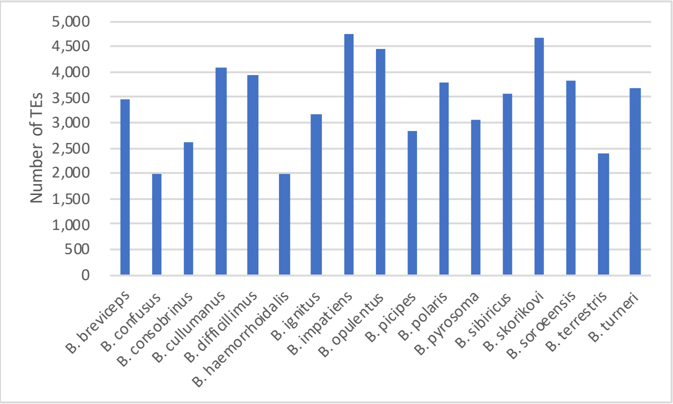

Figure S14. TE proliferation history in *Mendacibombus* species (*B. superbus* and *B. waltoni*) and in two representative non-*Mendacibombus* species (*B. terrestris* and *B. turneri*).

Red arrows indicate the amplification peak of *Mendacibombus* species in sequence divergence from ancestral sequences (at the top of the figure) and in Million years ago (Mya) (at the bottom), respectively. Here we focused on when the peaks of TE amplification occurred in those species and compared the peaks of *Mendacibombus* species with that of non-*Mendacibombus* species. Note that y-axis maxima differ. From the figure we could see that non-*Mendacibombus* species (*B. terrestris* and *B. turneri*) have a more recent amplification peak than that of *Mendacibombus* species (*B. superbus* and *B. waltoni*), indicating their more recent TE amplification activity.

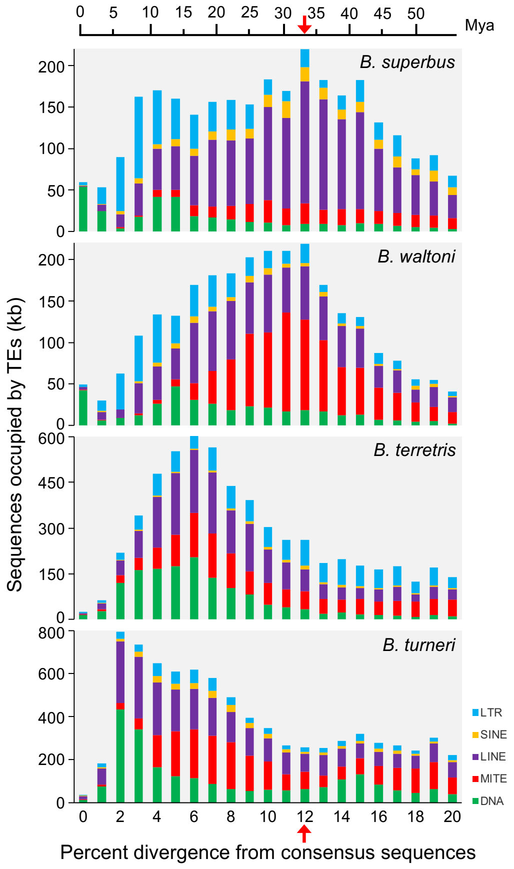

Figure S15. Example of a TE contributing to the coding sequences of a bumblebee gene. The locus, which is universal and single-copy in the 19 species, encodes a PFB0145c-like protein, and has an average length of 1,340 amino acids. This figure shows the multiple alignment of the 3' end of protein sequences (using MAFFT; full sequences are in Supplementary tables Table S32). Red lines above the alignment indicate amino acid sequences derived from the reverse transcriptase of an R1 retrotransposon. Below the alignment, log2 transformed depth of coverage (y axis) for RNA-seq reads mapped to the target region are shown in blue, supporting the expression of this chimeric TE-gene fusion. Only the first of portion (the first ~60 AA) of the TE-derived sequence is highly conserved across all species, whereas the rest of the sequence (for which there is also transcriptional support) could be functional, but clearly is not highly conserved across all the species.

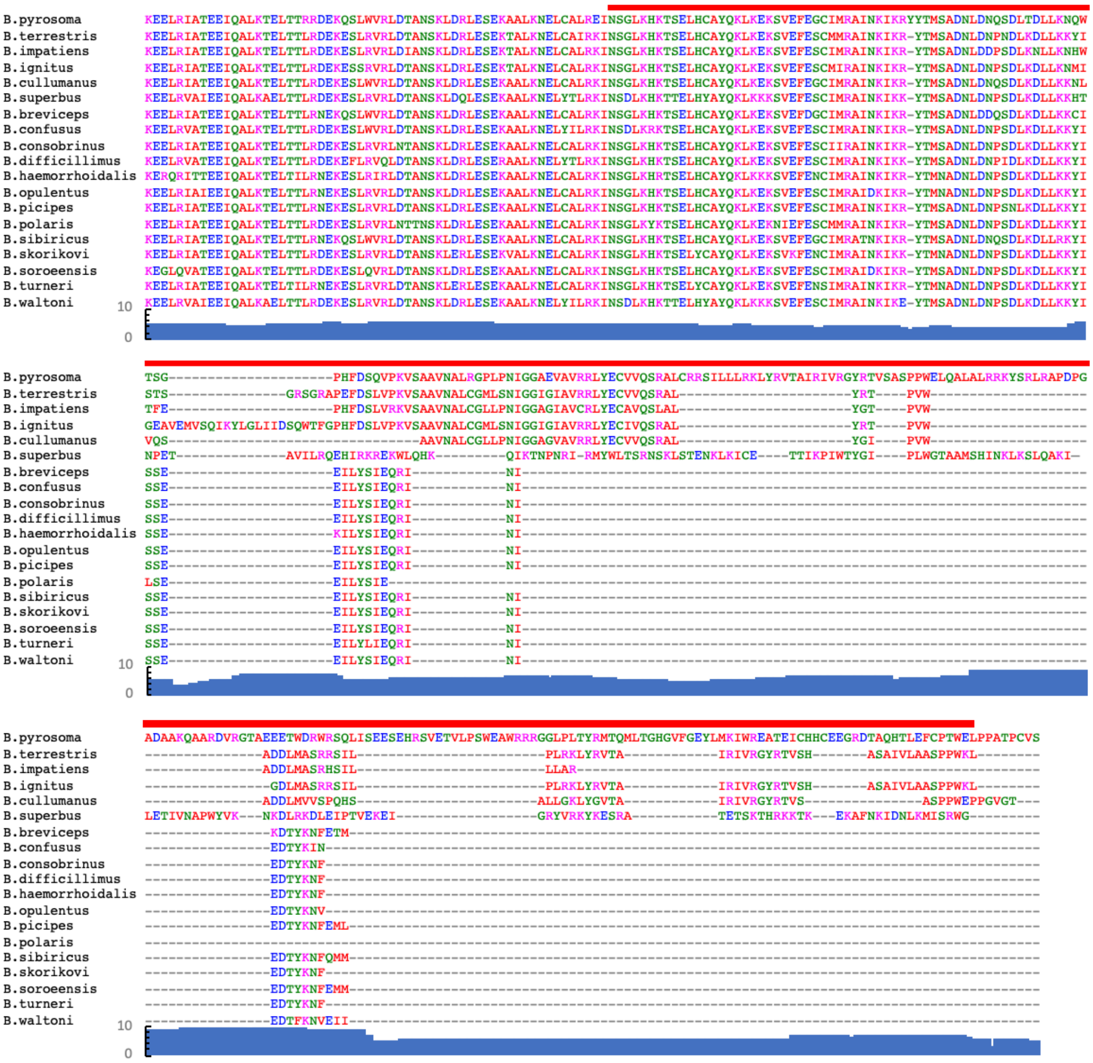

Figure S16. Gene turnover rates across the *Bombus* phylogeny.
Turnover rates reflect both gains and losses over time and were calculated as ((genes gained + genes lost) / time tree branch length / total genes).

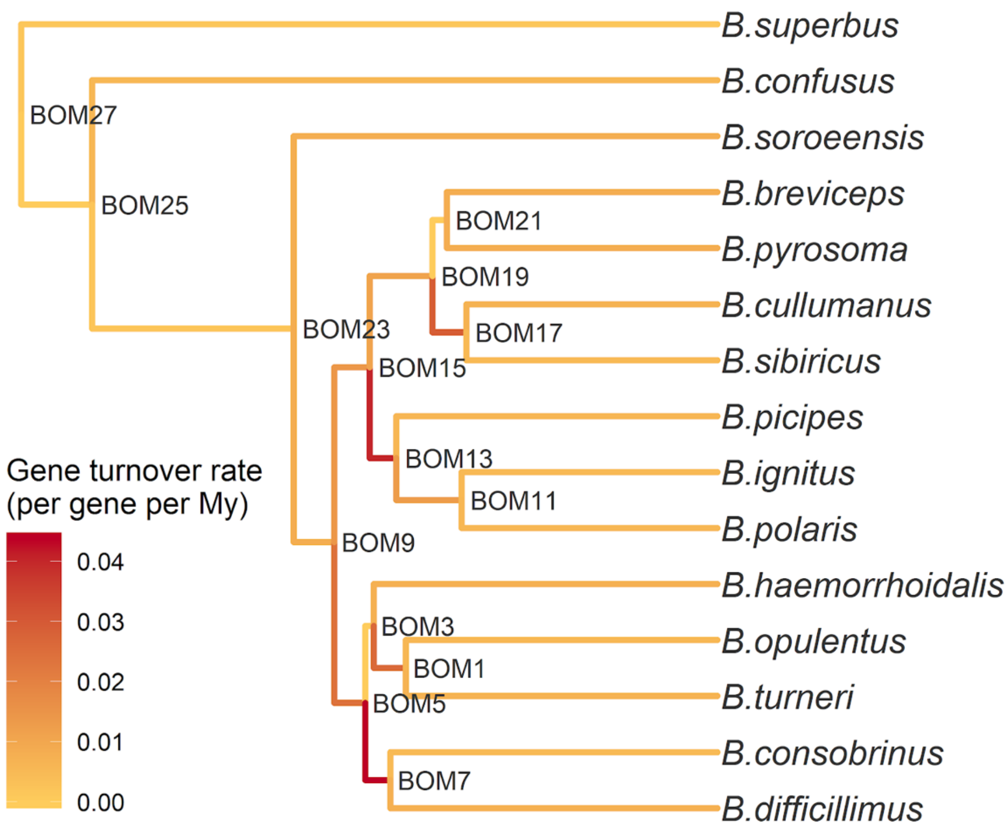

Figure S17. Example of stop codon readthrough in *B. terrestris*.

Alignment of the readthrough region of transcript rna11916 in gene XM_012313001.2, color coded by CodAlignView (https://data.broadinstitute.org/compbio1/cav.php). Also shown are the third ORF, and 10 codons on each end. All substitutions in both the second and third ORFs are synonymous (light green), a strong indication that these regions are protein-coding, which would indicate that both the annotated TGA stop codon and the subsequence TAG stop codon are read through, making this a double-readthrough gene. After the TGA stop codon that ends the third ORF, there are many non-synonymous substitutions (red and dark green), frame shifting indels (grey and orange), and stop codons, typical of non-coding regions. Most protein-coding regions have some non-synonymous substitutions, so the readthrough extension of the rna11916 protein is unusually well conserved. The evolutionary coding potential as measured by PhyloCSF of the 38-codon second ORF (180.4) and the 57-codon third ORF (295.5) are more than 2000 times as likely to occur in coding regions than non-coding regions, implying that it these extensions have been functional at the amino acid level in much of the bee tree. The perfectly conserved TGA-C stop codon context is known to promote inefficient termination.

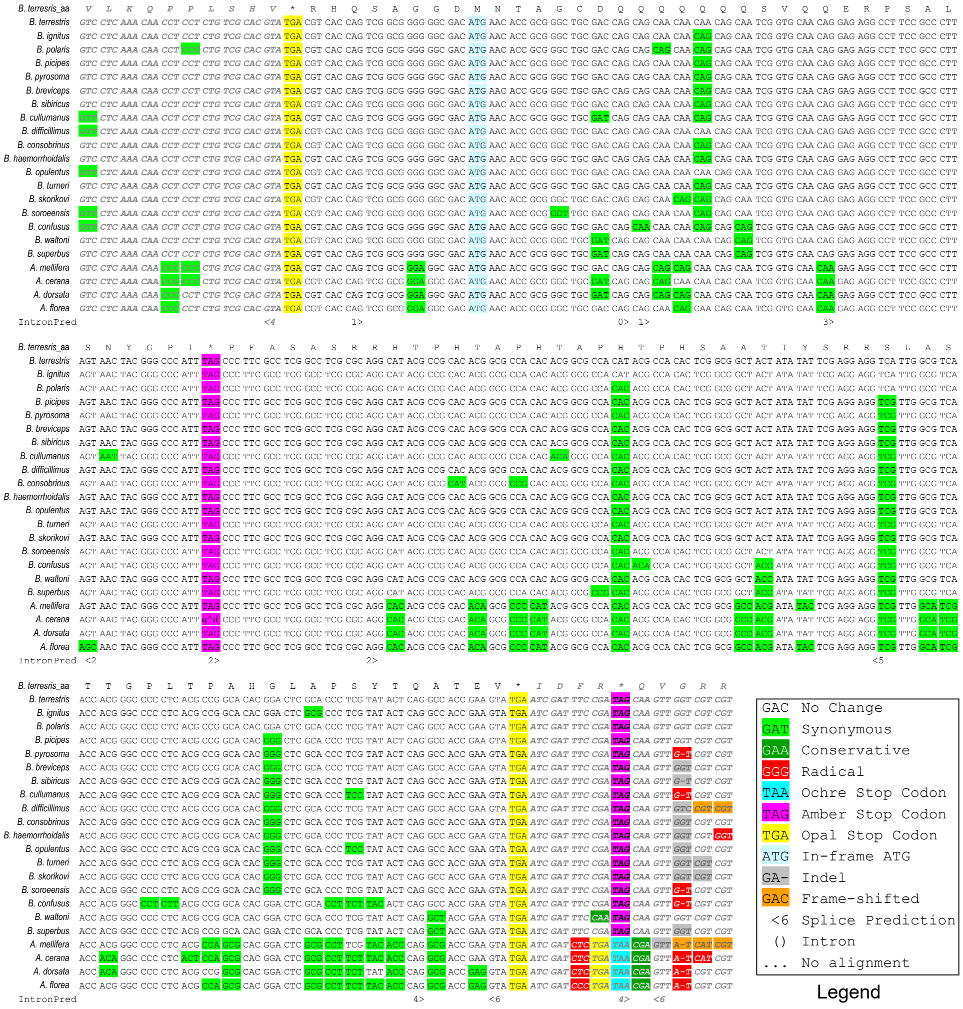

Figure S18. Tree with nodes labeled for Malin analysis.

Species names in the tree are in short and their corresponding full names are as follow: Bpici (*B. picipes*), Bigni (*B. ignitus*), Bpyro (*B. pyrosoma*), Bturn (*B. turneri*), Bsupe (*B. superbus*), Bsoro (*B. soroeensis*), Bcull (*B. cullumanus*), Bpola (*B. polaris*), Bhaem (*B. haemorrhoidalis*), Bconf (*B. confusus*), Bsibi (*B. sibiricus*), Bcons (*B. consobrinus*), Bopul (*B. opulentus*), Bskor (*B. skorikovi*), Bbrev (*B. breviceps*), Bdiff (*B. difficillimus*), Bwalt (*B. waltoni*), Bterr (*B. terrestris*), Bimpa (*B. impatiens*) , Amell (*Apis mellifera*).

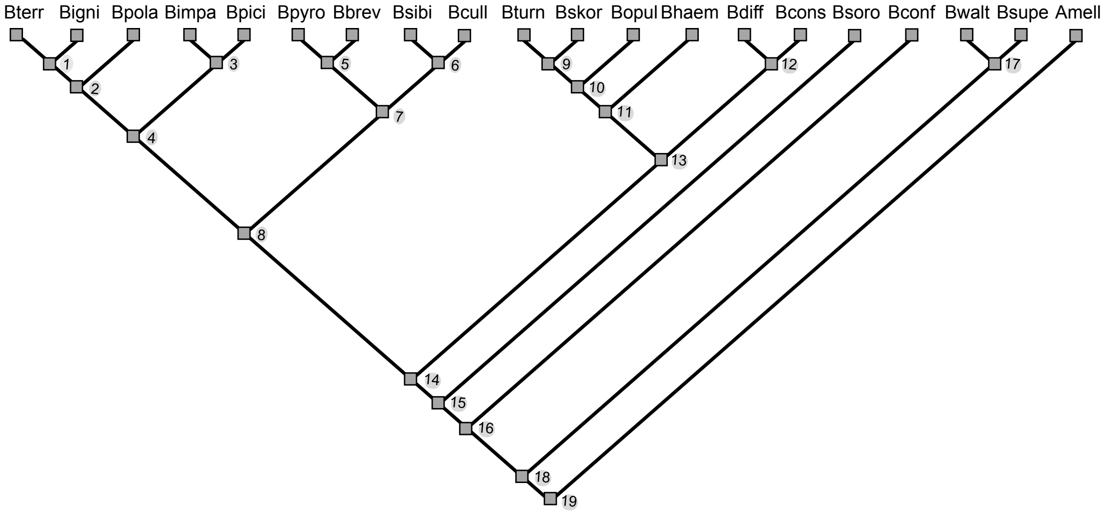

Figure S19. Functional annotation bias towards conserved genes.

Histograms show value distributions for all orthologous groups and for orthologous groups with genes that could be assigned by (**A**). Biological process GO terms (in green), (**B**). Molecular function GO terms (in green), and (**C**). InterPro domains (in green).

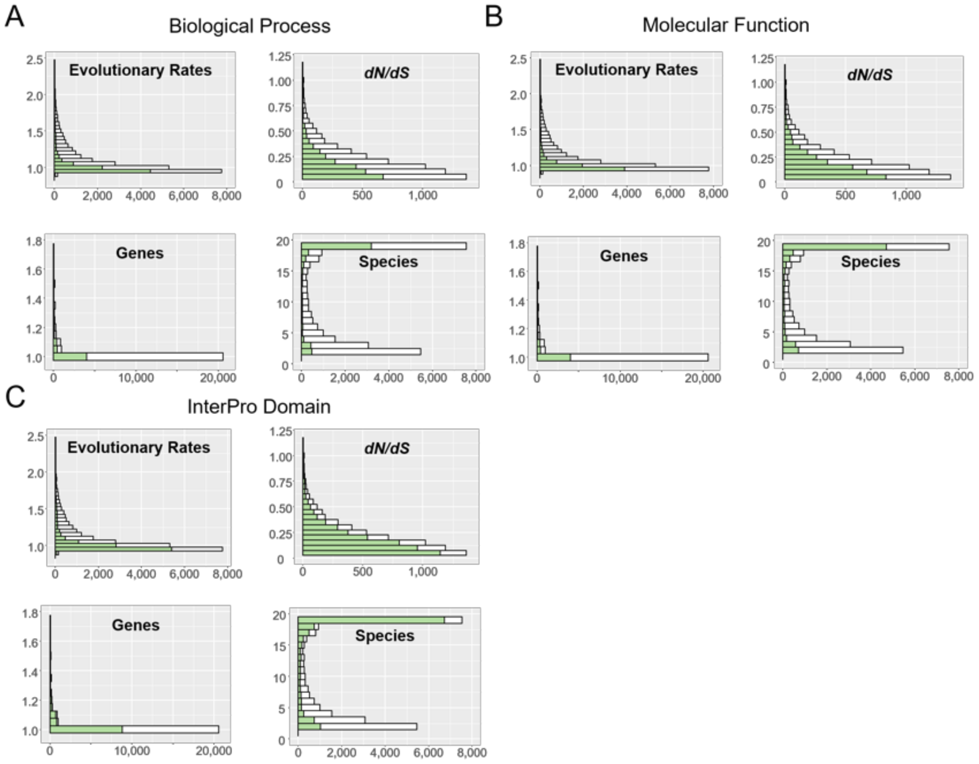

Figure S20. Molecular evolution of protein-coding genes.

Evolutionary rate (amino acid sequence divergence measured as the mean of normalized inter-species ortholog protein sequence identities, computed as part of the OrthoDB orthology delineation procedure) and *dN/dS* ratio among gene functional classes categories by (**A**) Molecular function Gene Ontology terms and (**B**) InterPro domains. Categories are sorted by evolutionary rate from the most conservative (left) to the most dynamic (right) and colored from the highest values (red) to the median value (blue) to the lowest values (orange). Notched boxes show medians of orthologous group values with the limits of the upper and lower quartiles, and box widths are proportional to the number of orthologous groups in each category.

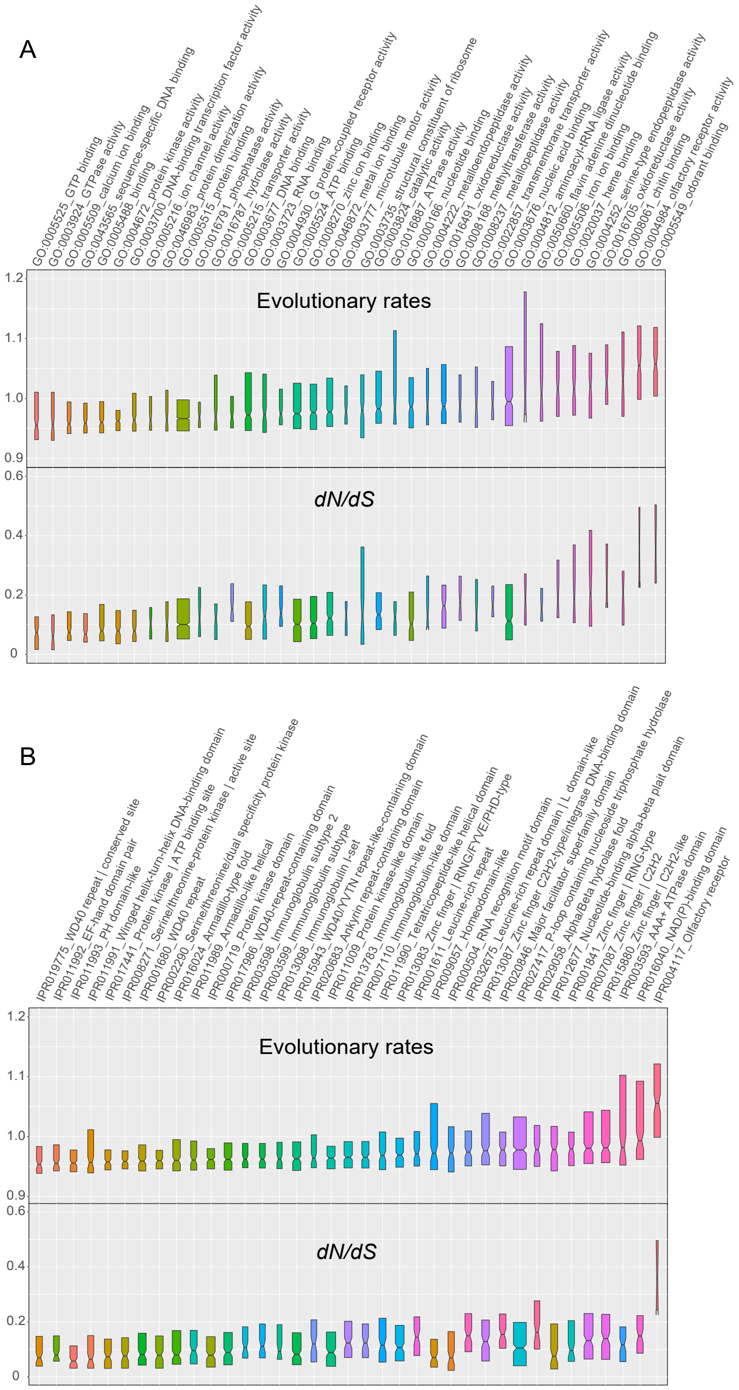

Figure S21. Evolutionary rate and *dN/dS* ratio distributions.

(**A**). Distribution of orthologous group evolutionary rates highlighting those less than the 20th percentile or greater than the 80th percentile. (**B**). Distribution of orthologous group *dN/dS* ratios highlighting those less than the 20th percentile or greater than the 80th percentile.

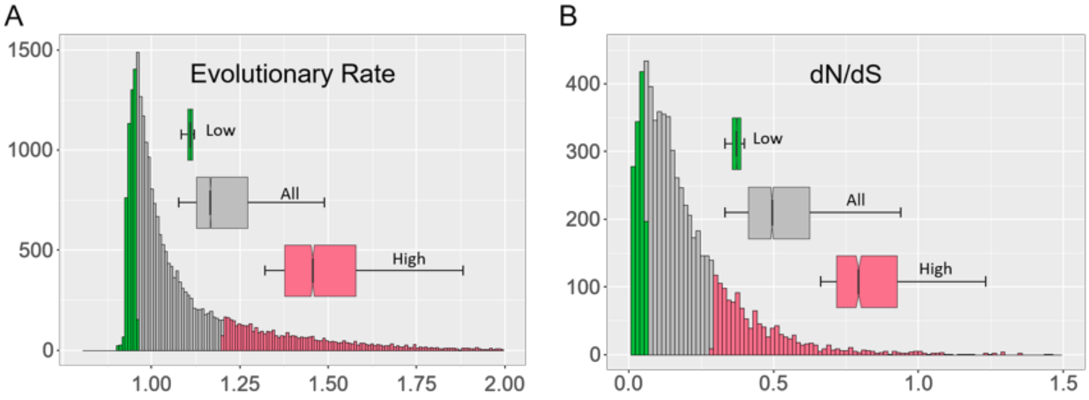

Figure S22. Correlation coefficient between RSCU and ENC.

RSCU and ENC estimated as the exponential of the sum of Shannon entropy of codon usage within each codon family across the 19 species in rows and the 64 codons in columns. Black lines separate codon family. Blue values indicate negative correlation, meaning preferred codon, given that high frequency of a codon correlates with decrease of entropy in codon usage across the genes and codon family.

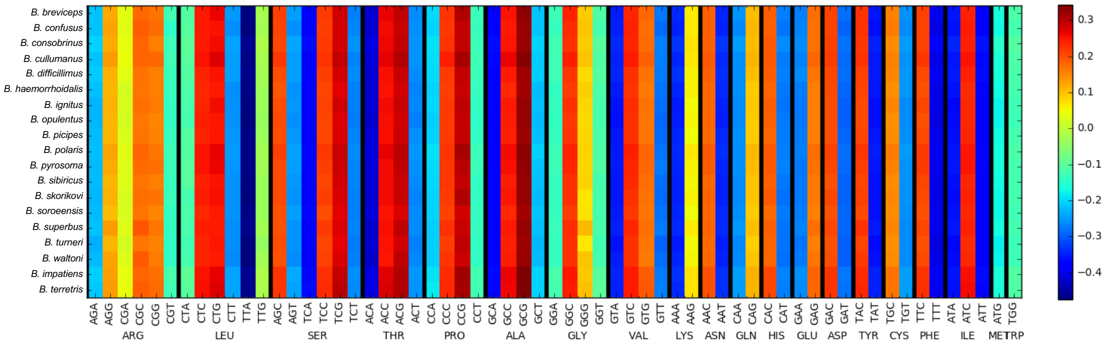

Figure S23. Correlation between gene AT content and the frequency of optimal codons. Optimal codons are defined as the most negatively correlated codon within each family (Data from Figure S22). The scale bar on the side indicates number of points in each hexagonal bin.

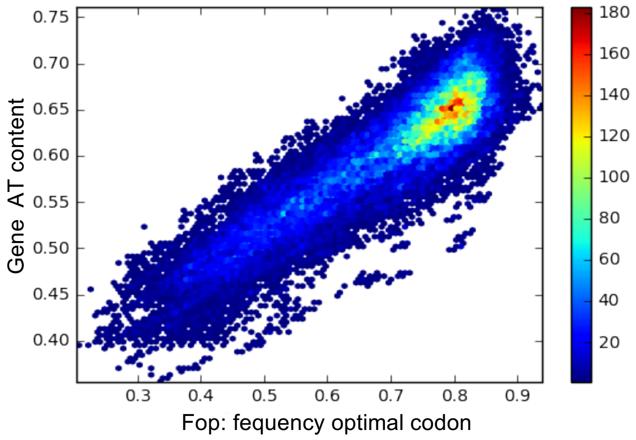

Figure S24: Relationship between codon AT content and correlation shown in Figure S22. Numbers of overlapping points within each hexagonal bin are defined in the color bar.

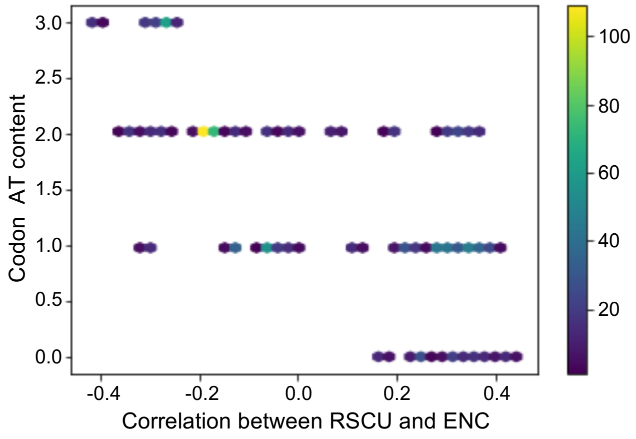

Figure S25. Evolutionary histories of chemosensory genes in bumblebees.

OR represents odorant receptor; GR represents gustatory receptors; IR represents ionotropic receptors. The results of CAFE and Notung are highlighted in blue and red, respectively. Number of intact chemosensory genes are shown beside species name. Numbers on each node are the estimated ancestral gene numbers. Numbers on branches indicate gene gain and loss events estimated by Notung.

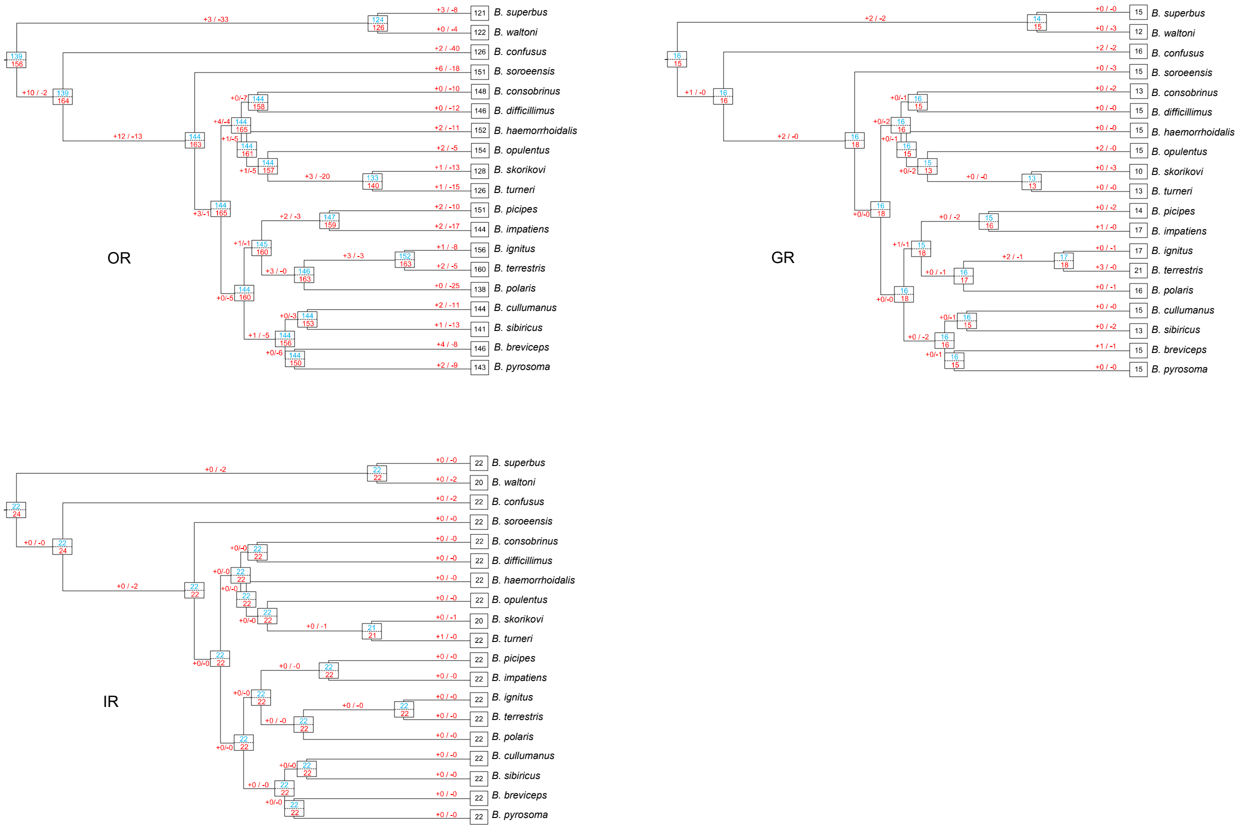

Figure S26. Sex-determination genes fem and fem1.

Example of amino acid motif distribution for *fem*, *fem1* (and *csd*) among *Bombus* and *Apis*, with highlighting of lineage specific (*fem1*) motifs and taking structural variation into account.

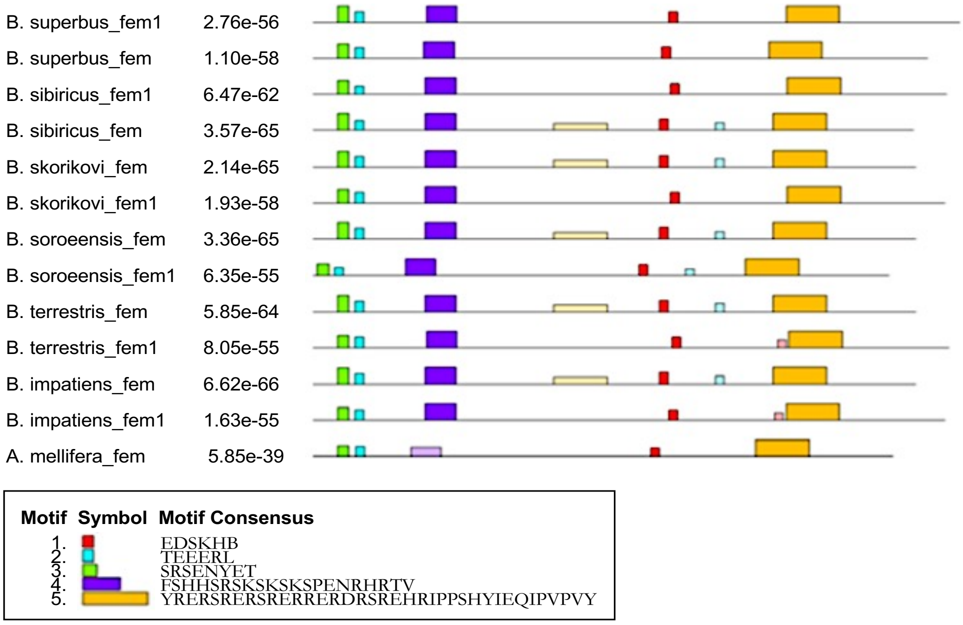

Figure S27. Sex-determination gene tra2.

Tra2 RNA recognition domain with RNA binding sites marked (in grey conserved; in red changed in *Apis* compared to *Bombus*).

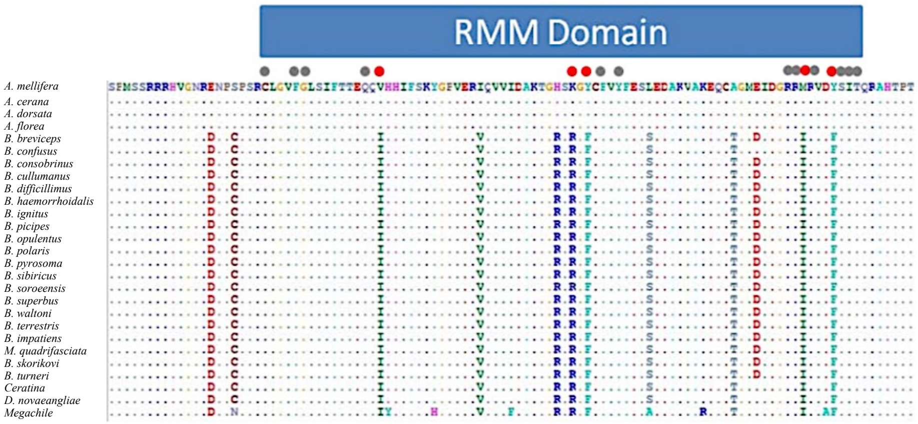

Figure S28. Comparisons of assembly contiguity with other genomic features.
Pearson correlation analysis between genome assembly contiguity (scaffold N50/Contig N50) and different genomic features that may influence assembly quality. The x-axis shows scaffold N50 values in all panels except panel I. Young TEs represent TE-derived sequences with <=2% divergence from their ancestral sequences. The trends show that the presence of more young TEs could be at least partially responsible for reduced contiguity by hindering the assembly process at the contig level (panel I).

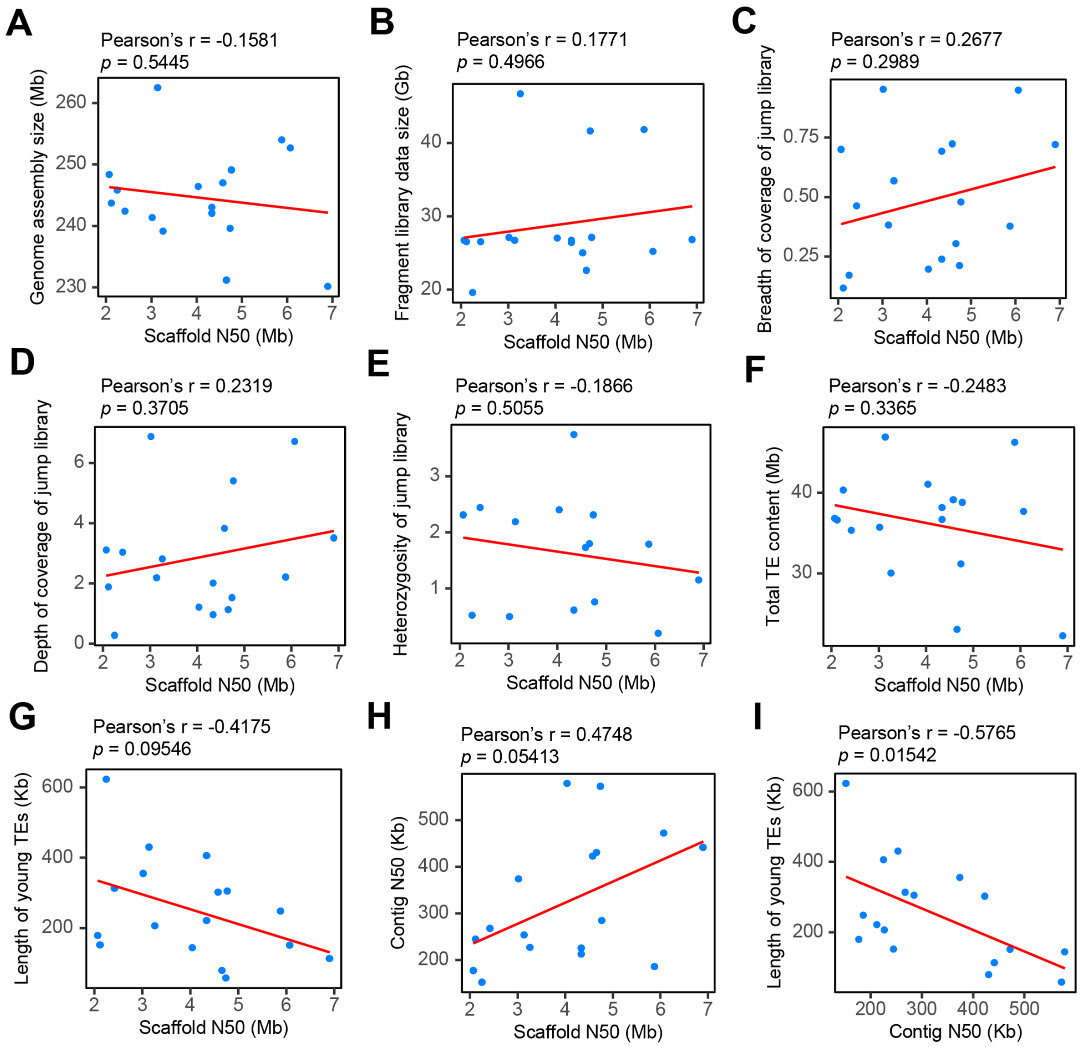

Supplement: msaa240_Supplementary_Data [file msaa240_supplementary_data.zip › msaa240-suppl_data/Supplementary figures.pdf]
