# Supplementary material for: Genus-Wide Characterization of Bumblebee Genomes Provides Insights into Their Evolution and Variation in Ecological and Behavioral Traits
Source: Mol Biol Evol. 2020 Sep 18;38(2):486–501. doi: 10.1093/molbev/msaa240 (PMC7826183; doi:10.1093/molbev/msaa240)
Supplement: msaa240_Supplementary_Data [file msaa240_supplementary_data.zip › msaa240-suppl_data/Supplementary Materials and Methods.docx]

***Genus-wide characterization of bumblebee genomes provides insights into their evolution and variation in ecological and behavioral traits***

**Supplementary Materials and Methods**

**Table of contents**

[Sample collection and DNA extraction 3](#_Toc49522969)

[Genome sequencing and assembly 4](#_Toc49522970)

[Genome annotation 5](#_Toc49522971)

[RNA extraction and sequencing 5](#_Toc49522972)

[Protein-coding gene annotation 6](#_Toc49522973)

[Functional annotation of the obtained gene models 7](#_Toc49522974)

[miRNA annotation 8](#_Toc49522975)

[tRNA annotation 9](#_Toc49522976)

[The prediction of LncRNAs 9](#_Toc49522977)

[Gene synteny analysis 9](#_Toc49522978)

[*De novo* identification and annotation of transposable elements (TEs) 10](#_Toc49522979)

[Methods based on TE structure 10](#_Toc49522980)

[Methods based on the repetitive nature of TEs 11](#_Toc49522981)

[TE landscapes in the bumblebee genomes 11](#_Toc49522982)

[TEs proliferated after the divergence of Mendacibombus from the other subgenera 12](#_Toc49522983)

[The age distribution of TE families in bumblebees 13](#_Toc49522984)

[The potential domestication of TEs in bumblebees 14](#_Toc49522985)

[Orthology delineation across *Apis* and *Bombus* 15](#_Toc49522986)

[Phylogeny construction 15](#_Toc49522987)

[Testing for introgression 17](#_Toc49522988)

[Estimate of ancestral genome sizes 18](#_Toc49522989)

[Hi-C library construction, sequencing, and assembly 19](#_Toc49522990)

[Macrosynteny search and visualization 22](#_Toc49522991)

[Evaluation of chromosomal evolution rates 23](#_Toc49522992)

[Global gene family evolution analysis 23](#_Toc49522993)

[Protein domain variation across bumblebees 25](#_Toc49522994)

[Molecular evolution analysis on gene functional categories 25](#_Toc49522995)

[Orthology delineation across bumblebees 25](#_Toc49522996)

[Assignment of functional categories to each orthologous group 26](#_Toc49522997)

[Evolutionary rate estimation for each orthologous group 26](#_Toc49522998)

[dN/dS ratio estimation for each orthologous group 26](#_Toc49522999)

[Enrichment analysis of the slowest and fastest evolving genes 27](#_Toc49523000)

[Intron evolution 29](#_Toc49523001)

[Stop codon readthrough analysis 30](#_Toc49523002)

[Whole genome alignments 30](#_Toc49523003)

[Stop codon readthrough analysis 30](#_Toc49523004)

[Codon usage bias analysis 33](#_Toc49523005)

[Gene family evolution analysis of chemosensory genes 34](#_Toc49523006)

[Identification of genes involved in detoxification 36](#_Toc49523007)

[Identification and characterization of immune genes 36](#_Toc49523008)

[Detection of positive selection signatures using aBSREL 37](#_Toc49523009)

[Evolutionary analysis of sex-determination genes 38](#_Toc49523010)

[Identification of genes involved in the adaptation of bumblebees to high elevation 40](#_Toc49523011)

[Evolution of piRNA genes 41](#_Toc49523012)

[List of supplementary tables S1-S33 42](#_Toc49523013)

[References 45](#_Toc49523014)

# Sample collection and DNA extraction

Criteria including phylogenetic position, biological trait, geographic distribution, and specimen availability were applied to select species for whole genome sequencing. A total of 17 bumblebee species were selected (Supplementary Tables Table S1), which span all of the 15 subgenera in the simplified classification system for the genus *Bombus* ([Williams et al. 2008](#_ENREF_93)). Among these, two species (*B. superbus* and *B. waltoni*) are from the subgenus *Mendacibombus*, which is sister to all other *Bombus* lineages; four species (*B. superbus*, *B. waltoni*, *B. skorikovi* and *B. difficillimus*) were collected at extremely high elevations (> 4000 m above sea level); two species (*B. turneri* and *B. skorikovi*) exhibit social parasitism; and one species (*B. polaris*) is endemic to the Arctic. In addition, species traits (i.e. range size, tongue length, parasite incidence, decline status, and life histories) vary across the selected bumblebees ([Williams 1994](#_ENREF_92); [Sikora and Kelm 2012](#_ENREF_73); [Persson et al. 2015](#_ENREF_62); [Arbetman et al. 2017](#_ENREF_3); [Cameron and Sadd 2020](#_ENREF_10)).

Samples were collected in the summer of 2016, with location and elevation information summarized in Supplementary Tables Table S1. Their identities were confirmed by DNA barcoding as described ([Hebert et al. 2004](#_ENREF_30)). Genomic DNA was extracted from each specimen using the Gentra Puregene Tissue Kit (Qiagen). The abdomens of each sample were removed before DNA extraction to avoid microbial contamination.

# Genome sequencing and assembly

Genomic DNA purified from one single haploid drone of each species was used to generate one “fragment” library with an insert size of 400 or 450 bp using the NEBNext^®^ Ultra^™^ DNA Library Prep Kit for Illumina^®^ (NEB, USA). The prepared fragment libraries were sequenced on an Illumina HiSeq 2500 platform with a read length of 250 bp to produce overlapping paired-end shotgun reads (2 × 250 bp), and the target sequencing coverage was 100-fold or more for each species. Genomic DNA purified from multiple specimens (from 3 to 5 individuals as detailed in Supplementary Tables Table S2) of each species was used to generate four “jump” libraries (insert sizes: 4 kb, 6 kb, 8 kb, and 10 kb) according to reported methods ([Heavens et al. 2015](#_ENREF_29)). The prepared jump libraries were sequenced on an Illumina HiSeq X Ten platform, and paired-end reads (2 × 150 bp) were generated, with a sequencing depth of at least 40-fold coverage for each jump library. The sequencing results of “fragment” and “jump” libraries are summarized in Supplementary Tables Table S2.

For each species, the 250 bp overlapping paired-end shotgun reads from the fragment library were processed using the software Seqtk (<https://github.com/lh3/seqtk>) to randomly subsample read pairs to achieve the total sequence length equivalent to ~60-fold sequencing coverage, a coverage recommended by the assembler we used (https://software.broadinstitute.org/software/discovar/blog/). Then, the subsampled shotgun reads were assembled using the software DISCOVAR *de novo* (version 52488), which performs well at assembling insect genomes ([Love et al. 2016](#_ENREF_54)), to produce contiguous sequences (contigs) for each species. DISCOVAR *de novo* is expressly designed to make optimal use of 2x250 bp reads coming from libraries with small insert sizes, i.e. exactly the properties of our fragment libraries, which result in consistent read overlap to stitch reads together. We therefore selected DISCOVAR *de novo* because it is tailored for use with the data we generated. Finally, shotgun reads from jump libraries were used to scaffold the contigs using the software BESST (Version 2.2.6) ([Sahlin et al. 2014](#_ENREF_71)). The obtained genome assemblies were checked for DNA contamination by searching against the NCBI non-redundant nucleotide database (Nt) using BLASTN ([Camacho et al. 2009](#_ENREF_8)), with an E-value cutoff of 1e-5.

To evaluate the quality and completeness of the genome assemblies, we compared genes present in the assemblies to a set of 4,415 universal single-copy orthologs (lineage dataset: hymenoptera_odb9) using the software BUSCO v3 ([Waterhouse et al. 2018](#_ENREF_89)).

# Genome annotation

## RNA extraction and sequencing

For each species (*B. superbus, B. waltoni, B. confusus, B. soroeensis, B. consobrinus, B. difficillimus, B. haemorrhoidalis, B. turneri, B. opulentus, B. picipes, B. ignitus, B. sibiricus, B. breviceps,* and *B. pyrosoma*), total RNA was isolated from the whole body (with abdomen being removed) of a single adult worker (for the social parasite species *B. turneri*, one adult male was used) by using the TRIzol reagent (Invitrogen, CA, USA) following the manufacturer’s instructions. RNA integrity was evaluated on a 1.0 % agarose gel stained with ethidium bromide. After quantifying the concentration of RNA using a Qubit® 2.0 Fluorometer (Life Technologies, CA, USA), 3 μg of RNA from each species was used to prepare sequencing libraries using the NEBNext® UltraTM RNA Library Prep Kit for Illumina® (NEB, USA) following manufacturer’s instructions. Library quality was assessed on the Agilent Bioanalyzer 2100 system. The prepared libraries were sequenced on the Illumina HiSeq X Ten platform, generating paired-end reads with a read length of 150 bp. RNA-seq results are summarized in Supplementary tables Table S33.

## Protein-coding gene annotation

Annotation of protein-coding genes was based on *ab initio* gene predictions, transcript evidence, and homologous protein evidence, all of which were implemented in the MAKER computational pipeline ([Cantarel et al. 2008](#_ENREF_11)). Briefly, RNA-seq samples were assembled using Trinity ([Haas et al. 2013](#_ENREF_26)) with two different strategies using default parameters, *de novo* assembly and genome-guided assembly. Assembled transcripts were inspected by calculation of FPKM (fragments per kilobase of exon per million fragments mapped) expression values and removed if FPKM <1 and iso-percentage <3%. The filtered transcripts were imported into the PASA program ([Haas et al. 2003](#_ENREF_25)) for construction of comprehensive transcripts, as PASA is able to take advantage of the high sensitivity of reference-based assembly while leveraging the ability of *de novo* annotation to detect novel transcripts (transcriptome assembly results are summarized in Supplementary tables Table S33). The nearly “full-length” transcripts selected from PASA-assembled transcripts were imported to data training programs including SNAP ([Korf 2004](#_ENREF_44)), GENEMARK ([Lomsadze et al. 2005](#_ENREF_53)) and AUGUSTUS ([Stanke et al. 2006](#_ENREF_77)). Afterwards, the MAKER pipeline was used to integrate multiple tiers of coding evidence and generate a comprehensive set of protein-coding genes.

The second round of MAKER was run to improve gene annotation. The predicted gene models with AED scores less than 0.2 were extracted for re-training using SNAP, GENEMARK, and AUGUSTUS. In addition, the RNA-seq reads were mapped to genomes using HiSAT2 and re-assembled using StringTie ([Pertea et al. 2016](#_ENREF_63)). The assembled RNA-seq transcripts, along with proteins from bees (superfamily Apoidea) that are available in NCBI GenBank (last accessed on 01/28/2018), were imported into the MAKER pipeline to generate gene models, followed by manual curation of key gene families.

## Functional annotation of the obtained gene models

To obtain functional clues for the predicted gene models, protein sequences encoded by them were searched against the Uniprot-Swiss-Prot protein databases (last accessed on 01/28/2018) using the BLASTp algorithm implemented in BLAST suite v2.28 ([Altschul et al. 1990](#_ENREF_1)). In addition, protein domains and GO terms associated with gene models were identified by InterproScan-5 ([Jones et al. 2014](#_ENREF_36)).

To evaluate the quality and completeness of gene annotation, we compared protein sequences predicted from the genome assemblies to a set of 4,415 universal single-copy orthologs (lineage dataset: hymenoptera_odb9) using the software BUSCO v3 ([Waterhouse et al. 2018](#_ENREF_89)).

## miRNA annotation

Hairpin sequences downloaded from miRBase (http://www.mirbase.org/) were aligned to each reference genome using BLASTN ([Altschul et al. 1990](#_ENREF_1)) with an e-value cut-off of 10-6. Results were further filtered based on alignment length (≥50nt) and sequence similarity (≥80%). Mature sequences from miRBase were then mapped against this set of selected BLASTN hits, using Patman ([Prufer et al. 2008](#_ENREF_65)) with parameters -g 0 -e 1 (no gaps, up to one mismatch). Only genomic hits where at least one mature microRNA could be mapped with these criteria were retained. These were treated as a set of putative homologous microRNA genes.

Small RNA reads of *B. terrestris* were mapped to these predicted homologous loci, with no gaps or mismatches allowed. Genomic loci with at least 10 mapped reads were then selected, showing coverage at both the 5’ and 3’ ends. The final set of high confidence microRNAs was obtained by selecting all loci with the expected hairpin secondary structure, as predicted by RNAfold from the ViennaRNA package ([Hofacker 2009](#_ENREF_34)), as well as strong evidence of Drosha-Dicer processing from the (manually inspected) patterns of small-RNA read alignments.

## tRNA annotation

All of the bumblebee genomes were screened with tRNAScan-SE ([Lowe and Eddy 1997](#_ENREF_55)) to identify tRNA genes, with default parameters.

## The prediction of LncRNAs

Protein-coding potential for RNA transcripts was predicted using two algorithms, LGC version 1.0 ([Wang et al. 2019](#_ENREF_84)) and CPAT version 2.0.0 ([Wang et al. 2013](#_ENREF_85)). LGC could be used in a cross-species manner and the algorithm was applied directly to bumblebees, while CPAT requires high-quality training data to build a species-specific model. Considering bumblebees do not have enough high-quality “coding” and “non-coding” transcripts to build a model, the prebuilt fly model in CPAT was used. All the predictions were performed on a Linux platform. RNA transcripts were deemed to be non-coding if they were consistently predicted to be non-coding by both LGC and CPAT.

# Gene synteny analysis

MCScanX ([Wang et al. 2012](#_ENREF_87)) was used to identify syntenic blocks, defined as regions with more than five collinear genes, between *B. terrestris*, a previously published bumblebee genome ([Sadd et al. 2015](#_ENREF_70)), and each of the newly sequenced bumblebees with default parameters to infer synteny contiguity.

# *De novo* identification and annotation of transposable elements (TEs)

## Methods based on TE structure

LTR retrotransposons of the bumblebee genomes were *de novo* identified and annotated by LTRharvest and LTRdigest ([Ellinghaus et al. 2008](#_ENREF_20); [Steinbiss et al. 2009](#_ENREF_78)). The identified LTR retrotransposons were further classified with the PASTEC module of the REPET package ([Hoede et al. 2014](#_ENREF_33)). When identifying LTR retrotransposons, TSD length was set to 4-6 bp and the minimum similarity of LTRs was set to 85%; the four-nucleotide termini of each LTR retrotransposon was set as TG…CA. LTR length was set to 100-6000 bp. For the post-processing of LTRdigest, pptlength was set to 10-30 bp, pbsoffset to 0-5 bp, and trans to Dm-tRNAs.fa. pHMMs were used to define protein domains taken from the Pfam database.

Non-LTR retrotransposons of the bumblebee genomes were identified and characterized using MGEScan-non-LTR, with default parameters ([Rho and Tang 2009](#_ENREF_66)).

DNA transposons were identified by TBLASTN of known DNA transposase sequences that are available in Repbase (<https://www.girinst.org/repbase/>) against the bumblebee genome sequences. All regions that produced significant hits (E-values <1E-10) were excised with 3 kb of flanking regions. The terminal inverted repeats of a DNA transposon were identified through a self-alignment of the excised sequence using NCBI-BLAST 2.

## Methods based on the repetitive nature of TEs

RepeatScout ([Price et al. 2005](#_ENREF_64)) was used to *de novo* identify repetitive sequences from bumblebee genomes, with default parameters. The obtained consensus sequences were classified by the PASTEC module of the REPET package ([Hoede et al. 2014](#_ENREF_33)). All of the repetitive sequences were classified into Class I (retrotransposons), Class II (DNA transposons), Potential Host Genes, SSR (Simple sequence repeats) and “noCat” (which means no classification was found).

## TE landscapes in the bumblebee genomes

First, CD-HIT-EST (version 4.6.6) ([Li and Godzik 2006](#_ENREF_49)) was used to parse TE sequences that were *de novo* identified based on structure and repetitive nature with a sequence identity threshold of 0.9 (other parameters as default) to reduce TE redundancy for each bumblebee species. Then, the remaining TE sequences from all the bumblebee species were combined to produce a comprehensive TE library. Using this repeat library, each bumblebee genome was analyzed with RepeatMasker (<http://www.repeatmasker.org>) to yield a comprehensive summary of the TE landscape in each species using Cross_Match as the search engine (other parameters as default). The annotation files produced by RepeatMasker were processed by in-house scripts to eliminate redundancy. Refined annotation files were used to determine the TE diversity and abundance within each species. Tandem repeats in each genome were identified by Tandem Repeat Finder ([Benson 1999](#_ENREF_6)), implemented in RepeatMasker.

## TEs proliferated after the divergence of Mendacibombus from the other subgenera

The subgenus *Mendacibombus* forms the sister group to all of the other extant bumblebees, diverging near the Eocene-Oligocene boundary approximately 34 million years ago ([Williams 1985](#_ENREF_91); [Cameron et al. 2007](#_ENREF_9); [Hines 2008](#_ENREF_31)). If a TE is present in one non-*Mendacibombus* species, but is absent at the orthologous positions in both *Mendacibombus* species (*B. superbus* and *B. waltoni*), then the TE is inferred to have transposed sometime after the divergence of the species from *Mendacibombus*. To identify such TEs in each of the non-*Mendacibombus* species, first, pairwise whole-genome alignments between the target species and *B. superbus* were performed using the software LASTZ ([Harris 2007](#_ENREF_28)). Then, based on the whole genome alignment results, TE insertion scanner (https://github.com/Adamtaranto/TE-insertion-scanner) was used to identify “alignment gaps” showing signatures of TE insertions in the genome of the target species, with “--maxInsert 50000 --minIdent 85 --minInsert 80” choices (other parameters set as default). Secondly, 200 bp of sequence flanking the identified TE-like insertion on either side were extracted from the genomic sequences of the target bumblebee species and combined into one sequence of 400 bp. Then, the flanking sequences were used as queries in BLASTn searches against the genomic sequence of *B. waltoni*, with an e-value cutoff of 1e-10. Hits spanning both sides of the TE-like insertion with a minimal length of 350 bp were considered as empty sites in *B. waltoni* genome. Finally, TE-like sequences that have identifiable orthologous empty sites in both of the two *Mendacibombus* species were RepeatMasked by the comprehensive TE library of bumblebees to confirm their TE identity.

## The age distribution of TE families in bumblebees

The consensus sequence of each TE family was constructed using RepeatScout ([Price et al. 2005](#_ENREF_64)) for each of the 19 bumblebee species; this consensus represents the TE family’s master gene (i.e. ancestral sequence). The obtained consensus sequences were used to produce a species-specific TE library. Using these libraries, each genome was masked with RepeatMasker. Percent divergences from consensus sequences reported by RepeatMasker were converted to nucleotide distance measures using the Jukes-Cantor formula to correct for multiple hits. To increase accuracy, analyses were limited to TE elements ≥80% identical to their respective consensus sequences, with a minimum length of 80 bp. Results were pooled into bins of single unit distances and represent summaries of TE class proliferation history. Because TEs evolve neutrally following insertion, the age of individual TEs can be approximated by measuring the sequence divergence from the ancestral consensus sequence and by applying a neutral substitution rate of 3.6 × 10^-9^ for bumblebee ([Liu et al. 2017](#_ENREF_52)).

# The potential domestication of TEs in bumblebees

The genomic coordinates of TEs in each species were compared with the coordinates of protein-coding genes in the same species to identify TEs that resided within or near predicted genes. Only when there were > 50 bp of overlap between a TE and predicted CDS was a TE considered to be overlapping with a coding region. Orthologous groups containing genes whose coding regions have TE-derived sequences were extracted, along with their overall *dN/dS* values (see Molecular evolution analysis on gene functional categories section) to check their *dN/dS* ratios to determine if they are under selective constraint. Bowtie2 (Langmead and Salzberg 2012) version 2.2.6 was employed to map RNA-seq reads produced in the study (Supplementary tables Table S33) to the coding DNA sequences (CDS) of genes containing TE-derived sequences. Then, SAMtools (<http://samtools.sourceforge.net/>) was used to calculate the depth of coverage for RNA-seq reads mapped to the target region. In *B. terrestris*, the coordinates of TEs, excluding those found in coding regions, were also compared with the coordinates of open chromatin regions detected by ATAC-seq ([Zhao et al. 2019](#_ENREF_100)) to identify TEs that may serve as regulatory sequences.

# Orthology delineation across *Apis* and *Bombus*

The locally installed OrthoDB pipeline (Kriventseva et al., 2015) was employed to define orthologous groups for proteins coming from 19 bumblebees and 4 honeybees. In addition to the 17 newly sequenced bumblebees from this study, the following previously annotated gene sets were downloaded: *B. terrestris* (GenBank assembly: Bter_1.0), *B. impatiens* (GenBank assembly: BIMP_2.0), *Apis mellifera* (GenBank assembly: Amel_4.5), *Apis cerana* (GenBank assembly: ACSNU-2.0), *Apis florea* (GenBank assembly: Aflo_1.0), and *Apis dorsata* (GenBank assembly: *Apis dorsata* 1.3). Only the longest isoform of each gene was used in orthology delineation. The orthoMCL program ([Li et al. 2003](#_ENREF_48)) was applied to the same protein dataset to confirm the results of the OrthoDB pipeline on lineage- and species-specific genes, and only genes determined as lineage- or species-specific by both programs were used for downstream analysis. In order to characterize the function of *Bombus*-specific genes, genes from *B. terrestris* that are *Bombus*-specific were selected. The GO annotations of *Bombus*-specific genes were assigned by InterproScan-5 ([Jones et al. 2014](#_ENREF_36)) and visualized on the WEGO website (<http://wego.genomics.org.cn/>; gene level 4) ([Ye et al. 2006](#_ENREF_96)).

# Phylogeny construction

To construct the phylogeny for these 23 species (19 bumblebees and 4 honeybees), universal single-copy orthologs delineated by the OrthoDB pipeline were isolated, and 3,617 single-copy orthologs were identified. Protein sequences from each of those universal single-copy orthologs were aligned with the software MAFFT ([Katoh et al. 2002](#_ENREF_42)), followed by alignment trimming with BMGE ([Criscuolo and Gribaldo 2010](#_ENREF_14)). Alignments shorter than 100 amino acids or with more than 50% identical sequences were removed, resulting in 2,918 orthologs for phylogeny reconstruction. Trimmed alignments were concatenated for each species, respectively, resulting in 23 long super-sequences. The super-alignment contained 1,801,503 sites with 218,400 distinct site patterns. IQ-TREE version 2.0 ([Minh et al. 2020b](#_ENREF_57)) was used to construct a maximum likelihood concatenated tree with the ultrafast bootstrap method ([Hoang et al. 2018](#_ENREF_32)). The best-fitting amino acid substitution model for each partition was selected by automatically by IQ-TREE’s internal implementation of ModelFinder ([Kalyaanamoorthy et al. 2017](#_ENREF_40)). A time calibrated, ultrametric tree was produced by using a non-parametric rate smoothing approach ([Sanderson 2003](#_ENREF_72)) along with a fossil calibration range of 65 My to 125 My for the divergence of *Apis* and *Bombus* ([Hines 2008](#_ENREF_31)). To assess phylogenetic discordance among loci, gene trees for each single-copy orthologous group were also reconstructed with IQ-TREE (Supplementary Tables Table S5) ([Minh et al. 2020b](#_ENREF_57)). Of the 2,918 gene trees, 4 were removed because the outgroup genus *Apis* was not monophyletic and 257 trees were removed because more than 50% of the branch lengths were 0, likely resulting from lack of variant sites. We also checked for trees with more than 50% of the lineages with bootstrap below 90, but no trees met this criterion. This resulted in a final set of 2,657 trees for analysis of discordance. (Supplementary Tables Table S6). Rooting was performed using Newick Utilities ([Junier and Zdobnov 2010](#_ENREF_39)). Gene and site concordance factors (CF) were then calculated for each node in the species tree as implemented in IQ-TREE ([Minh et al. 2020a](#_ENREF_56)). The quartet-based species tree reconstruction program ASTRAL ([Zhang et al. 2018](#_ENREF_99)), which can account for ILS, was also used for building the species phylogeny. The ggtree R package was used to visualize trees ([Yu et al. 2017](#_ENREF_97)).

## Testing for introgression

Though the rapid diversification of the bumblebees is favorable for ILS driving discordance, we also tested for introgression. For each lineage in the IQ-TREE species tree with a gCF < 95% we calculated the Δ statistic (Huson et al. 2005). This statistic follows the same logic as the ABBA-BABA site patterns used to calculate D-statistics, but uses tree topologies instead of alignment sites. Briefly, a given lineage in an unrooted tree is defined by a quartet of species groupings with two possible discordant topologies, $D_{1}$ and $D_{2}$. Under assumptions of ILS, both discordant topologies should be present in equal proportions. However, if introgression has occurred one discordant topology will appear more frequently than the other. Δ is calculated as follows (Vanderpool et al. 2020):

$$\Delta=\frac{D_{1}-D_{2}}{D_{1}+D_{2}}$$

This normalized Δ calculation ensures all values are between 0 and 1, with larger values indicating a larger skew towards one topology, and a higher chance that introgression has occurred. To test whether the observed Δ values are skewed significantly from 0 to imply introgression, we performed the same analysis 1,000 bootstrap replicates of our calculated gene trees to generate a null distribution. We then calculated Z-scores and p-values and assessed significance at a threshold of 0.01 (Eaton and Ree 2013).

# Estimate of ancestral genome sizes

The genome assemblies produced in this study were highly complete (Supplementary figures Figure S1), and genome assembly sizes do not correlate with assembly contiguity (p = 0.5445; Supplementary figures Figure S28A). Thus, smaller genome size estimates are unlikely to be artifacts of incomplete genome assembly, and quality control during assembly ensured that larger genomes were not due to extrinsic DNA contamination. Therefore, the genome assembly sizes should reflect true differences across bumblebees. Genome assembly sizes of the 19 sequenced bumblebees and four honeybees were obtained from the current study and published genome assemblies: *B. terrestris* (GenBank assembly: Bter_1.0), *B. impatiens* (GenBank assembly: BIMP_2.0), *Apis mellifera* (GenBank assembly: Amel_4.5), *Apis cerana* (GenBank assembly: ACSNU-2.0), *Apis florea* (GenBank assembly: Aflo_1.1), and *Apis dorsata* (GenBank assembly: *Apis dorsata* 1.3). Genome sizes were mapped onto the phylogenetic tree estimated in this study (Figure 1A), and ancestral genome sizes of bumblebees were estimated using parsimony ancestral state reconstruction in Mesquite 3.51 (<http://www.mesquiteproject.org>), with honeybee genome sizes serving as the outgroup.

# Hi-C library construction, sequencing, and assembly

For *B. turneri*, library preparation was performed by Annoroad Gene Technology (http://en.annoroad.com) and mainly followed a protocol described previously ([Belton et al. 2012](#_ENREF_5)). Briefly, thorax muscles of wild-caught males were cross-linked by 2% formaldehyde solution at room temperature for 20 mins, and 2.5 M glycine was added to quench the crosslinking reaction. After grinding with liquid nitrogen, homogenized tissues were resuspended in 25 ml of extraction buffer I (10 mM Tris-HCl [pH 8.0], 5 mM β-mercaptoethanol, 0.4 M sucrose, 10 mM MgCl2, 0.1 mM phenylmethylsulfonyl fluoride [PMSF], and 1x protease inhibitor [Roche]), then filtered through miracloth (Calbiochem). The filtrate was centrifuged at 3,500g at 4°C for 20 min. The pellet was resuspended in 1 ml of extraction II (10 mM Tris-HCl [pH 8], 0.25 M sucrose, 10 mM MgCl2, 1% Triton X-100, 5 mM β-mercaptoethanol, 0.1 mM PMSF, and 1x protease inhibitor) and then centrifuged at 18,400g and 4 °C for 10 min. The pellet was resuspended in 300 μl of extraction buffer III (10 mM Tris-HCl, [pH 8.0], 1.7 M sucrose, 0.15% Triton X-100, 2 mM MgCl2, 5 mM β-mercaptoethanol, 0.1 mM PMSF, and 1 x protease inhibitor) and loaded on top of an equal amount of extraction buffer III, then centrifuged at 18,400g for 10 min. The supernatant was discarded and the pellet was washed twice by resuspending it in 500 μl of ice-cold 1x CutSmart buffer, followed by centrifuging the sample for 5 min at 2,500g. The nuclei were washed by 0.5 ml of 1 x restriction enzyme buffer and transferred to a safe-lock tube. Next, the chromatin was solubilized with dilute SDS and incubated at 65 °C for 10 min. After quenching the SDS with Triton X-100, overnight digestion was applied with a four-cutter restriction enzyme (400 units of MboI) at 37 °C on a rocking platform. The flowing steps include marking the DNA ends with biotin-14-dCTP and performing blunt-end ligation of crosslinked fragments. The proximal chromatin DNA was re-ligated by ligation enzyme. The nuclear complexes were reverse-crosslinked by incubating with proteinase K at 65 °C. DNA was purified by phenol–chloroform extraction. Biotin-C was removed from non-ligated fragment ends using T4 DNA polymerase. Fragments were sheared to a size of 100–500 bp by sonication. The fragment ends were repaired by the mixture of T4 DNA polymerase, T4 polynucleotide kinase, and Klenow DNA polymerase. Biotin-labeled Hi-C samples were specifically enriched using streptavidin magnetic beads. A-tailing of the fragment ends were added by Klenow (exo-) and Illumina paired-end sequencing adapters were added by ligation mix. Finally, Hi-C sequencing libraries were amplified by PCR (12-14 cycles) and sequenced on the Illumina HiSeq X Ten platform, generating paired-end reads (2 × 150 bp). The Juicer tool ([Durand et al. 2016](#_ENREF_18)) was applied to map Hi-C reads against the scaffold sequences of *B. turneri* using the BWA algorithm ([Li and Durbin 2010](#_ENREF_47)) with default parameters. Mapped reads with MAPQ quality scores ≥ 30 were chosen for the next analysis. Then, the 3D-DNA pipeline ([Dudchenko et al. 2017](#_ENREF_17)) was applied to assemble the scaffold sequences to the chromosome level.

For *B. ignitus*, *B. pyrosoma*, *B. breviceps*, and *B. haemorrhoidalis*,

the *in situ* Digestion-ligation-only Hi-C protocol was employed to generate Hi-C reads as described ([Lin et al. 2018](#_ENREF_50)). In brief, for each species, brain tissue of wild-caught workers was ground into homogenate. Treated the samples and filtered the precipitated cells. Cells were double cross-linked with formaldehyde with EGS (Thermo) and 1% formaldehyde (Sigma). After that, the remaining formaldehyde was sequestered with glycine. The cross-linked cells were subsequently lysed in lysis buffer and incubated at 50 °C for 5min, placed on ice immediately. After incubation, the nuclei were digested by MseI (NEB, 100 units/μl). After restriction enzyme digestion, MseI biotin linkers were ligated to the digested chromatin respectively. Made the nuclei fragment-end phosphorylation. Next, added T4 DNA ligase (Thermo) to reaction complexes. Ligation was performed at 20 °C for 2h with rotation at 15 r.p.m. Then, purifying the proximity ligation DNA. The purified products were digested by MmeI at 37 °C for 1 h. The digested DNA sample was subjected to electrophoresis in native PAGE gels and the specific 80-bp DLO Hi-C DNA fragments were excised and purified. Next, Illumina sequencing adaptors were ligated to the 80-bp DLO Hi-C DNA fragments. After biotin incubation, the ligated DNA fragments were used as template and amplified by PCR (fewer than 13 cycle) to construct the Illumina sequencing libraries.

Hi-C sequencing libraries were sequenced on the Illumina HiSeq X Ten platform, generating 150 bp reads. The length of the DNA constructs in the DLO Hi-C library is between 78 and 82 bp. The length of a full linker is 40 bp, and the lengths of the target DNA sequences on each side of the linker are 19-21 bp. A Java program was used to exclude the linker parts from the reads and the target DNA fragments were used for downstream analysis. The Juicer tool ([Durand et al. 2016](#_ENREF_18)) was applied to map obtained target sequences against the scaffold sequences of each species using the BWA algorithm ([Li and Durbin 2010](#_ENREF_47)), selecting the ALN parameter (other parameters as default). Mapped reads with MAPQ quality scores ≥ 30 were chosen for the next analysis. Then, the 3D-DNA pipeline ([Dudchenko et al. 2017](#_ENREF_17)) was applied to assemble the scaffold sequences to the chromosome level.

The coordinates of genes within scaffold sequences were converted into coordinates on chromosome sequences for those five species.

# Macrosynteny search and visualization

First, the longest CDS for each gene, along with their coordinates, were prepared for the bumblebee species with chromosome-level assemblies (*B. ignitus*, *B. pyrosoma*, *B. breviceps*, *B. haemorrhoidalis*, *B. terrestris* and *B. turneri*). Then, pairwise comparisons were performed between *B. turneri* and each of the other species using MCscan in the JCVI tool kit (https://github.com/tanghaibao/jcvi; last accessed Dec 25, 2019) ([Wang et al. 2012](#_ENREF_87)) to identify and visualize macrosynteny.

# Evaluation of chromosomal evolution rates

Orthologous genes and their coordinates on chromosomes were used as anchors to evaluate rates of chromosomal evolution. Two sets of orthologous genes for each pair of species were grouped together to form a standard input for the GRIMM-Synteny program v. 2.02 ([Tesler 2002](#_ENREF_82)). The genome of *B. terrestris* was used as a reference for pairwise comparisons with other species genomes. Chromosomes of different species with similar sets of genes were named chromosomal elements. The GRIMM-Synteny program was run with default settings and the rearrangement distances (the number of conserved synteny blocks and inversions) were summarized.

# Global gene family evolution analysis

In order to identify rapidly evolving gene families within *Bombus*, protein sequences from the following species were used: *B. superbus, B. confusus, B. soroeensis, B. consobrinus, B. difficillimus, B. haemorrhoidalis, B. turneri, B. opulentus, B. picipes, B. ignitus, B. polaris, B. cullumanus, B. sibiricus, B. breviceps, and B. pyrosoma* (one species per subgenus was selected to avoid over-sampling in any subgenus). To ensure that each gene was counted only once, only the longest isoform of each gene in each species was used. An all-vs-all BLAST ([Altschul et al. 1997](#_ENREF_2)) search was then performed on these filtered sequences. The resulting e-values from the search were used as the main clustering criterion for the MCL program to group proteins into gene families ([Enright and J. 2002](#_ENREF_21)). This resulted in 24,137 clusters. All clusters only present in a single species or not present at the root of the tree were then removed, resulting in 13,828 gene families. A time calibrated, ultrametric tree (Supplementary figures Figure S16) was built by taking the inferred *Bombus* phylogeny and using a non-parametric rate smoothing approach ([Sanderson 2003](#_ENREF_72)) along with a fossil calibration range of 65 My to 125 My for the divergence of *Apis* and *Bombus* ([Hines 2008](#_ENREF_31)).

With the gene family data and ultrametric phylogeny as input, gene gain and loss rates (λ) were estimated with CAFE v3.0 ([Han et al. 2013](#_ENREF_27)). This version of CAFE is able to estimate the amount of assembly and annotation error (ε) present in the input data using a distribution across the observed gene family counts and a pseudo-likelihood search. CAFE is then able to correct for this error and obtain a more accurate estimate of λ. The resulting ε value was about 0.05, which implies that 5% of gene families have observed counts that are not equal to their true counts. After correcting for this error rate, λ = 0.0036. Using the estimated λ value, CAFE infers ancestral gene counts and calculates p-values across the tree for each family to assess the significance of any gene family changes along a given branch. Those branches with low p-values are inferred to be rapidly evolving. A Fisher’s exact test was performed on GO terms for genes in families that are rapidly evolving on any lineage vs. all other families, with a false discovery rate of 0.01.

# Protein domain variation across bumblebees

Predicted protein sequences were analyzed by InterproScan-5 ([Jones et al. 2014](#_ENREF_36)) to identify InterPro domains in each bumblebee species. InterPro domain annotations across the 19 bumblebee species were used to identify protein domains exhibiting the highest variation in gene counts across bumblebees. A crude measure that highlights such variation in copy-number was computed as the standard deviation divided by the mean of the bumblebee gene counts matching a particular InterPro domain. Results were filtered to focus on abundant domains, which have more than 200 genes in total and more than five genes in each bumblebee species.

# Molecular evolution analysis on gene functional categories

## Orthology delineation across bumblebees

In addition to the 17 newly sequenced bumblebees from this study, we downloaded the two previously annotated gene sets for *B. terrestris* and *B. impatiens* from Ensembl (http://metazoa.ensembl.org/index.html). Only the longest isoform of each gene was used for downstream analysis. Protein sequences from the 19 bumblebees were used to delineate orthologous groups by locally installed OrthoDB software (OrthoDB_soft_2.4.4) (<http://www.orthodb.org/software>).

## Assignment of functional categories to each orthologous group

GO term(s) and InterPro domain(s) associated with each gene of the orthologous group were identified by InterproScan-5 ([Jones et al. 2014](#_ENREF_36)). A GO term or InterPro domain was assigned to this orthologous group if more than 60% of the genes in it were assigned this GO term or InterPro domain by InterproScan-5.

## Evolutionary rate estimation for each orthologous group

Evolutionary rates (amino acid sequence divergence measured as the mean of normalized inter-species ortholog protein sequence identities) were computed for each orthologous group as the average of inter-species identities normalized to the average identity of all inter-species best reciprocal hits, computed from pairwise Smith-Waterman alignments of protein sequences. The ‘evolrate’ program of the OrthoDB_soft_2.4.4 package was used to obtain these rates (Waterhouse et al. 2011).

## dN/dS ratio estimation for each orthologous group

To avoid biases related to duplication among lineages and out-paralog genes, only universal single-copy orthologous groups (scOGs) were used to estimate *dN/dS* ratios. Protein sequences of scOGs were aligned by MAFFT ([Katoh et al. 2002](#_ENREF_42)) and then used to inform CDS alignments to generate DNA codon alignments with the codon-aware PAL2NAL program ([Suyama et al. 2006](#_ENREF_80)). Next, the aligned CDSs were trimmed by Gblocks ([Talavera and Castresana 2007](#_ENREF_81)), with “-t c” and other parameters as default. After trimming, only orthologs consisting of aligned sequences from all species with a minimum of 150 bp and less than 20% Ns were retained for downstream analysis, which are available on-line (ftp://download.big.ac.cn/bumblebee/bumblebee-single-copy-orthologs.tar.gz). Then, based on trimmed alignments, Maximum Likelihood trees were constructed for each of the orthologous groups using RAxML-NG ([Kozlov et al. 2019](#_ENREF_45)). Finally, PAML ([Yang 2007](#_ENREF_94)) was used to calculate the *dN/dS* ratio for each orthologous group using its respective phylogenetic tree (codeml model=0, NSsites=0, ncatG=1).

## Enrichment analysis of the slowest and fastest evolving genes

Assignment of GO terms and InterPro domains was biased towards slower-evolving, well-conserved genes (Supplementary figures Figure S19), so the fastest evolving genes are less likely to be functionally annotated. Comparing the top enriched functional categories in the slowest and fastest subsets of genes could complement the GO and InterPro analyses described above. Orthologous groups with evolutionary rates and *dN/dS* ratios less than the 20th percentile or greater than the 80th percentile were selected to represent the slowest and fastest gene sets, respectively (Supplementary figures Figure S21). Enrichment tests on GO Biological Processes and Molecular Functions were performed using Bioconductor’s GOstats hypergeometric test ([Falcon and Gentleman 2007](#_ENREF_23)) and with the topGO (http://www.bioconductor.org/packages/release/bioc/html/topGO.html) implementations of the classic Fisher and the weighted Fisher tests. The background gene sets in each case were genes from all 19 bumblebee genomes that were classified into any orthologous group and were annotated with Biological Process or Molecular Function GO-terms. The results were combined using a conservative strategy: terms must appear significant with a p-value <0.05 for all three enrichment tests, and there must be more than five genes in the test set. Complementary enrichment analyses using topGO’s implementation of the Kolmogorov–Smirnov (KS) were performed using evolutionary feature metrics: evolutionary rate (as above); universality (the proportion of species with genes in each orthologous group); and three copy-number metrics (average copy-number, copy-number variation, and proportion of species with duplicates). Only Biological Process terms associated with at least 10 orthologous groups were assessed. The KS test uses the score distributions directly without having to specify any top or bottom cut-off as described above for the classic tests with the 20th and 80th percentiles. Results are presented for terms showing significantly higher or significantly lower score distributions (Supplementary Tables Table S14 and Table S19).

# Intron evolution

Orthologous groups delineated across 19 bumblebees and one honeybee (*A. mellifera*) were examined to select a total of 8,672 with near-universal single-copy orthologue distributions: requiring no more than two species with no orthologues and no more than two species with multi-copy orthologues. These were further filtered to exclude groups with genes for which annotation features did not match the protein sequence and groups where the orthologues from five or more of the 20 species were single-coding-exon genes (i.e. no introns), leaving 7,394 groups for the analysis. The protein sequences of the orthologues for each group were FASTA formatted with header information containing intron/exon data required for analysis with Malin ([Csűros 2008](#_ENREF_15)). Protein sequences for each group were aligned with MAFFT v7.310 ([Kazutaka and Standley 2013](#_ENREF_43)) using the ‘--auto’ option. The resulting alignments were then processed (two rounds of re-alignment) by the IntronAlignment tool from the Malin suite with option ‘-matrix blosum62 -rep 2’. The species tree and alignments were loaded into the Malin analysis tool and reliable intron sites were defined as having at least five non-gap amino acid positions in the alignment before and after the site and unambiguous characters in at least 18 of the 20 species. This resulted in a total of 45,804 sites for the analysis which was performed using the Bootstrap Posterior Probability (BPP) approach of Malin, using rate models computed from the default starting model with default optimization parameters and with one gain and one loss level.

# Stop codon readthrough analysis

## Whole genome alignments

Before multiple whole genome alignments, repetitive regions of the 19 bumblebee and 4 honeybee (*Apis mellifera*, *Apis cerana*, *Apis florea*, and *Apis dorsata*) genome assemblies were first masked to reduce the total number of potential genomic anchors formed by the many matches that occur among regions of repetitive DNA. For whole genome alignments of the 23 bees, Cactus ([Paten et al. 2011](#_ENREF_61)), a reference-free whole genome aligner, was used. The phylogeny of 23-species estimated in this study (Figure 1A), with branch lengths reflecting neutral substitutions per site, was used as the guide tree.

## Stop codon readthrough analysis

Annotation version GCF_000214255.1 for *B. terrestris*, obtained from NCBI, was used. The phylogeny is the 23-species maximum likelihood phylogeny estimated in this study. PhyloCSF ([Lin et al. 2011](#_ENREF_51)) was run on the region between the annotated stop codon (“first stop codon”) and the next in-frame stop codon (“second stop codon”) referred to as the “second open reading frame (ORF)”, excluding both the first and second stop codons, of all annotated transcripts whose coding region ends in a stop codon, grouping together sets of transcripts having the same second ORF. For transcripts lacking an annotated 3’UTR, or for which the 3’UTR does not extend up to the second stop codon, the transcript was extended along the DNA strand without splicing. PhyloCSF was run using the default “mle” strategy and “bls” option, using the 12flies parameters but substituting the 23-bees tree. PhyloCSF computes a log-likelihood of an alignment under coding and non-coding models of evolution. The model assumes independence of codons given that the region is coding or non-coding. However, scores of neighboring codons are not independent. To correct for that, PhyloCSF-Ψ ([Lin et al. 2011](#_ENREF_51)) calculates a log-likelihood of length-dependent normal distributions trained on actual coding and non-coding regions of various lengths. Coefficients for PhyloCSF-Ψ were trained using coding regions at the ends of coding ORFs and non-coding regions at the starts of third ORFs, as described in ([Jungreis et al. 2016](#_ENREF_37)). The coefficients we obtained for *B. terrestris* were:

μ_C_ = 0.678782322375, A_C_ = 8.09766004622, B_C_ = 0.783878652717

μ_N_ = -6.80739917655, A_N_ = 9.51882863955, B_N_ = 0.664609908575

Both raw PhyloCSF scores and PhyloCSF-Ψ scores are reported in units of decibans. The 851 candidate readthrough stop codons in 817 genes were those satisfying all of the following conditions: (i) The second ORF is at least 10 codons long. (ii) PhyloCSF-Ψ > 0. (iii) The phylogenetic branch length of aligned species is more than 60% of the branch length of the full tree (enough to assure at least one *Apis* species is included). (iv) Species comprising at least 90% of the tree have the same first stop codon as *B. terrestris* (the *Drosophila* and *Anopheles* studies had found that readthrough stop codons are generally perfectly conserved). (v) Species comprising at least 60% of the tree have some stop codon aligned to the second stop codon. (vi) For second ORFs that overlap an annotated coding region on the same strand in the same reading frame, or on the opposite strand in the frame having the same third codon position (the “antisense” frame), the non-overlapping portion was required to be at least 10 codons long and have a positive PhyloCSF-Ψ score, as well as satisfying the branch length requirements described above.

To estimate the false discovery rate among our candidates, enrichment of the TGA stop codon with 3’ base C was used, which is known to be the “leakiest” 4-base stop codon context ([Bonetti et al. 1995](#_ENREF_7)) and is highly enriched among readthrough stop codons ([Jungreis et al. 2011](#_ENREF_38)). Of the 851 stop codons in the list, 172 (20.2%) have the TGA-C context, whereas of the 8059 annotated stop codons for which the second ORF has negative PhyloCSF-Ψ score and are thus unlikely to be readthrough, only 280 (3.5%) have the TGA-C context. Among the readthrough stop codons previously reported in *Drosophila* 32.2% had the TGA-C context ([Jungreis et al. 2011](#_ENREF_38)). If a similar fraction holds in *Bombus*, the number of actual readthrough stops codons among the 851 would be approximately (172 - 3.5% × 851) / (.322 - .035) = 496. Even if as many as 50% of readthrough stop codons in *Bombus* use TGA-C, a similar calculation provides a conservative estimate that the list includes 306 readthrough transcripts. Among the 200 of the candidates with highest PhyloCSF-Ψ score, 72 have TGA-C stop context, so a similar calculation conservatively estimates 140 readthrough transcripts among these 200 candidates, for a false discovery rate of no more than 30%.

# Codon usage bias analysis

Codon usage bias, the preferential use of specific synonymous codons, is a pattern maintained by mutation–selection–drift balance. The selection is linked to the efficiency and/or accuracy of translation. The selective effect of codon usage is only slightly advantageous and consequently selection’s efficiency depends on population size ([Vicario et al. 2007](#_ENREF_83); [Subramanian 2008](#_ENREF_79)); species with larger population sizes have more efficient selection for codon usage bias. Within the genomes, strength of selection could vary based on the stage of development when the genes are mainly translated ([Vicario et al. 2007](#_ENREF_83)). To determine the evolutionary forces affecting codon usage bias across bumblebees, a set of universal orthologous protein-coding genes was used (delineated in the **Molecular evolution analysis on gene functional categories** section). A total of 3,521 genes, which are present in all 19 species and have at least 50 unambiguous codons (no N or other ambiguity letters), were used for codon bias analysis. Candidate optimal codons were defined by examining the correlation between overall gene codon usage bias and the preference of use of a single codon as performed previously ([Vicario et al. 2007](#_ENREF_83)). As an estimator of overall codon usage bias, the Effective Number of Codons (ENC) was used, which was estimated by using the exponential of the sum of Shannon entropy of each codon family frequency set. As an estimator of preference for a single codon, the relative synonymous codon usage (RSCU) was used.

# Gene family evolution analysis of chemosensory genes

To detect the putative chemosensory genes of the three major gene families odorant receptors (ORs), gustatory receptors (GRs) and ionotropic receptors (IRs) from the 17 newly sequenced and the *B. impatiens* genomes, TBLASTN searches (with 1e-5 as the e-value cutoff) ([Gertz et al. 2006](#_ENREF_24); [Karpe et al. 2016](#_ENREF_41)) were performed using the protein sequences of *A. mellifera* ([Robertson and Wanner 2006](#_ENREF_67)) and *B. terrestris* ([Sadd et al. 2015](#_ENREF_70)) as queries. Putative chemosensory gene-containing regions were extracted from each genome to predict gene models using the protein2genome module of Exonerate v2.2.0 ([Slater and Birney 2005](#_ENREF_74)). These putative gene-containing regions were separately re-examined if there were no good hits based on Exonerate. Candidate chemosensory genes were further manually refined and checked for the characteristic domains of ORs (IPR004117), GRs (IPR009318 or IPR013604), or IRs (IPR019594 or IPR001320) in their encoded protein sequences using InterProScan v5.27-66.0 ([Zhou et al. 2012](#_ENREF_102); [Jones et al. 2014](#_ENREF_36); [Zhou et al. 2015](#_ENREF_101)). Partial sequences were completed with the nearest START and/or STOP codons wherever possible. Probable amino acid sequences of pseudogenes, which were identified using in-frame STOP codons or frameshifts, were determined from their predicted coding regions, and the letter “X” was used to represent STOP codons and frameshifts. The letter “Z” denotes unknown amino acids. The same procedure was repeated, using newly identified chemosensory genes as queries, until no additional genes were found. Gene names were assigned following the closest homologue of *B. terrestris*. When there were two or more gene copies in one analyzed species but a single-copy in *B. terrestris*, candidate gene names were suffixed with a, b, c, and so on. For ORs and GRs, genes encoding intact proteins with a length >= 350 amino acids were kept for downstream analysis.

Multiple alignments of the available bumblebee chemosensory genes were generated using MAFFT v7.407 (“E-INS-i strategy) ([Kazutaka and Standley 2013](#_ENREF_43)), poorly aligned regions in the alignments were filtered using TrimAl v1.4 (“automated1” option) ([Capellagutierrez et al. 2009](#_ENREF_12)), and maximum-likelihood phylogenetic trees were estimated using RAxML v8.2.11 (with the “PROTCATJTTF” model and 100 bootstrap replicates) ([Stamatakis 2014](#_ENREF_76)). To estimate the numbers of gains and losses of chemosensory genes, we used maximum-likelihood-based and parsimony-based approaches, respectively; all genes of each chemoreceptor family were used as input for CAFE v4.2 ([De Bie et al. 2006](#_ENREF_16)) with default settings, and gene trees were reconciled with species tree using Notung v2.9.1 ([Chen et al. 2000](#_ENREF_13)).

# Identification of genes involved in detoxification

Glutathione-S-transferases (GSTs), carboxyl/cholinesterases (CCEs), and cytochrome P450 monooxygenases (P450s) are involved in the detoxification of xenobiotics. To identify detoxication genes in the newly sequenced bumblebees, annotated P450, GST, and CCE protein sequences of *B. terrestris*, *A. mellifera*, and *D. melanogaster* were used as queries to search against the predicted protein sequences from each genome using BLASTp ([Altschul et al. 1990](#_ENREF_1)). If certain genes appeared to be missing, TBLASTn was used as in annotating chemosensory genes. All of the identified detoxication genes were further checked for the presence of their characteristic domains to confirm their identity (GST [IPR004045 and IPR010987], P450 [IPR001128], and CCE [IPR002018]).

# Identification and characterization of immune genes

To identify immune-related genes in the newly sequenced bumblebees, annotated immune genes of *B. terrestris* and *A. mellifera* were used as queries to search against the predicted protein sequences from each genome using BLASTp ([Altschul et al. 1990](#_ENREF_1)). If certain genes appeared to be missing, TBLASTn was used as in annotating chemosensory genes.

Immune genes were classified into three broad functional categories — “recognition,” “signaling,” and “effector” — based on previous reports ([Evans et al. 2006](#_ENREF_22); [Sackton et al. 2007](#_ENREF_69); [Barribeau et al. 2015](#_ENREF_4); [Neafsey et al. 2015](#_ENREF_59); [Waterhouse et al. 2020](#_ENREF_88)). Specifically, the recognition class includes SCR (scavenger receptors), GNBP (gram-negative binding proteins), PGRP (peptidoglycan recognition proteins), and GALE (galectins). The signaling class includes TOLL (toll-like receptors), JAKSTAT (Jak/Stat pathway members), IMDPATH (Imd pathway members), CLIP (CLIP-domain serine proteases), SRPN (serine protease inhibitors), CASP (caspases), and IAP (inhibitors of apoptosis). The effector class includes SOD (superoxide dismutases), TEP (thioester-containing proteins), LYS (lysozymes), PPO (prophenoloxidases), PRDX (peroxidases), AMP (anti-microbial peptides), ML (MD2-like proteins), NIMROD (nimrod-related proteins), FREP (fibrinogen-related proteins), and CTL (C-type lectins).

# Detection of positive selection signatures using aBSREL

For gene families of special interest (i.e. chemosensory genes, detoxification genes, and immune genes) and for genes identified in the initial PAML scans as potentially showing signatures of adaptation to high elevation (see below), we uploaded data to Datamonkey web-server (<https://www.datamonkey.org>) to detect positive selection. The brief procedures are as follow:

(1) Single-copy orthologous groups search: orthologous groups containing focal genes, along with their *dN/dS* values, were extracted from the **Molecular evolution analysis on gene functional categories** section. To avoid biases related to duplication among lineages and out-paralog genes, only universal single-copy orthologous groups were kept for downstream analysis.

(2) Multiple sequence alignment and *de novo* gene tree construction: The multiple alignment and Maximum Likelihood tree of each ortholog were taken from the **Molecular evolution analysis on gene functional categories** section.

(3) Detect recombination within the dataset using the algorithms implemented in the GARD software (https://www.datamonkey.org/gard).

(4) aBSREL analysis: For each ortholog, signatures of positive diversifying selection were searched using the aBSREL algorithm (<https://www.datamonkey.org>), with the respective multiple sequence alignment and tree topology. Branches with test p-values < 0.05 were considered to be under selection.

# Evolutionary analysis of sex-determination genes

Protein sequences of *B. terrestris* genes including *feminizer* (*fem*), *feminizer* 1 (*fem* 1), and *transformer 2*, which are involved in the sex determination pathway, were used as queries to search against the newly sequenced genomes by locally installed BLAST ([Gertz et al. 2006](#_ENREF_24)) to identify their orthologs/paralogs in bumblebees. Before phylogenetic analysis, sequences were multiply aligned using MUSCLE ([Edgar 2004](#_ENREF_19)). The evolutionary history of sex-determining genes in *Bombus* and related species was inferred using Maximum Likelihood with the JTT matrix-based model implemented in MEGA X ([Jones et al. 1992](#_ENREF_35); [Kumar et al. 2018](#_ENREF_46)). The tree with the highest log likelihood (-6161.36) is shown. A discrete Gamma distribution was used to model evolutionary rate differences among sites (5 categories (+G, parameter = 2.2)) with branch lengths measured in the number of amino acid substitutions per site. RELAX ([Wertheim et al. 2015](#_ENREF_90)) was employed to test whether the strength of natural selection was relaxed or intensified along a specified set of test branches. The spurious action of diversifying selection in a subset of branches was detected by aBSREL ([Smith et al. 2015](#_ENREF_75)). To further identify individual sites that were subject to episodic diversifying selection, the mixed effect model of evolution (MEME) was applied ([Murrell et al. 2012](#_ENREF_58)). GARD analysis to test for potential recombination suggested one breakpoint (at position 637) with the maximum relative breakpoint support of 1. This splits the data set in two fragments (fragment 1: 636bp and fragment 2: 177bp), located at the border between the predicted Arginine/Serin (R/S)-rich and the Proline (P)-rich-domain. As observed in the full-length dataset (*fem*_Bombus_ *dN/dS* = 0.24; *fem 1*_Bombus_ *dN/dS* = 0.77; Figure 4C), *dN/dS* ratios are in a similar range when calculated separately within each fragment (fragment 1: *fem*_Bombus_ *dN/dS* = 0.31, *fem 1*_Bombus_ *dN/dS* = 0.76; fragment 2: *fem*_Bombus_ *dN/dS* = 0.27, *fem 1*_Bombus_ *dN/dS* = 0.95). In addition, using the hypothesis branch-site testing framework (RELAX), the observation of relaxation of selection in *fem 1*_Bombus_ compared to *fem*_Bombus_ holds in the separate calculation for both, fragment 1 (p < 0.001, LR = 20.69) and fragment 2 (P<0.001, LR = 36.34). It remains to be further investigated if and to what extent the observed potential breakpoint has contributed to the evolutionary history of *fem1* and *fem* in all or only some *Bombus* species.

# Identification of genes involved in the adaptation of bumblebees to high elevation

To identify genes involved in high-elevation adaptation, searches were conducted for genes undergoing positive selection in *B. superbus*, *B. waltoni*, *B. difficillimus*, and *B. skorikovi*, which were all collected at elevations > 4,000 m (Figure 1). First, universal single-copy orthologous groups were obtained, along with their respective multiple sequence alignments and Maximum Likelihood trees (described in the **Molecular evolution analysis on gene functional categories** section). Then, the improved branch-site model in the Codeml program of the PAML package was used to identify genes showing signatures of positive selection ([Zhang et al. 2005](#_ENREF_98)). In brief, *B. superbus*, *B. waltoni*, *B. difficillimus*, and *B. skorikovi* (all collected at elevations > 4,000 m) were assigned as the foreground branches and all the other bumblebee species (all collected at elevations < 2,000 m) as the background branches. A positive selection model that allowed a class of codons on the foreground branches to have *dN/dS* > 1 (model = 2, NSsites = 2, omega = 0.5|1.5, fix_omega = 0) was compared with a null model that constrained this class of sites to have *dN/dS* = 1 (model = 2, NSsites = 2, omega = 1, fix_omega = 1) using a likelihood ratio test and calculated a p-value for each comparison. Multiple comparisons were corrected for by using the Benjamini and Hochberg method and selected genes with an adjusted p-value < 0.05 as candidate positively selected genes (PSGs). Then, the Bayes Empirical Bayes (BEB) method ([Yang et al. 2005](#_ENREF_95)) was used to calculate posterior probabilities for site classes to identify codon positions that experienced positive selections (*dN/dS* > 1). Candidate PSGs that also contained codon positions showing significant BEB values (posterior probability >95%) were further analyzed using the software aBSREL ([Smith et al. 2015](#_ENREF_75)) to identify genes that show positive selection in at least two subgenera of high-elevation species but not in any of the low-elevation species. Such genes were believed to be PSGs involved in high-elevation adaptation. Finally, Codeml was used to estimate *dN*, *dS*, and *dN/dS* of these PSGs with the free ratio model (model = 1, NSsites = 0). PSGs with *dS* >1, suggesting considerable saturation at the synonymous sites, were removed from downstream analysis to avoid false positives. Functional clues about the identified PSGs were obtained by BLAST searching against the UniProt database (<https://www.uniprot.org>) and by literature review.

# Evolution of piRNA genes

Protein sequences for *Ago1*, *Armitage*, *Eggless*, *Gasz*, *Hen1*, *Maelstrom*, *Minotaur*, *Papi*, *Piwi/Aub*, *Qin*, *Shutdown*, *Spindle-E*, *Squash*, and *Trimmer* in *Apis mellifera* were downloaded from GenBank based on the dataset used by ([Wang et al. 2017](#_ENREF_86)). A BLAST protein database was built from the transcriptomes of each *Bombus* species and selected the top BLASTp hits for each species. We restricted our analyses to proteins that were present and had a single copy for all of the species.

Protein sequences were aligned using PSY-Coffee and automatically trimmed using G-Blocks while allowing for smaller final blocks and gap positions within the final blocks ([Notredame et al. 2000](#_ENREF_60); [Talavera and Castresana 2007](#_ENREF_81)). Phylogenies were estimated in MrBayes 3.2 with *Apis mellifera* set as the outgroup ([Ronquist et al. 2012](#_ENREF_68)). A mixed model for amino acid evolution was used. Each analysis ran for 10 million generations with the sampling frequency set to 1,000 with 3 heated chains, and 25% of the trees discarded as burnin.

The trimmed multiple alignments of single-copy orthologous groups containing piRNA genes, along with their phylogenies, were extracted from **Molecular evolution analysis on gene functional categories** section. Positive selection was detected by aBSREL ([Smith et al. 2015](#_ENREF_75)). However, analysis of branch lengths and positive selection for 14 piRNA pathway genes across bumblebees found neither to be associated with genome size.

# List of supplementary tables S1-S33

Table S1. Collection site information of the 17 newly sequenced bumblebees.

Table S2. Genome sequencing results of the 17 newly sequenced bumblebees.

Table S3. Genome annotation results.

Table S4. TE landscape in each sequenced bumblebee.

Table S5. Unrooted concatenated gene trees constructed by IQ-TREE.

Table S6. The final set of concatenated gene trees after filtering.

Table S7. Gene topology count.

Table S8. Rates of chromosomal evolution for 5 species in pairwise comparison with B. terrestris.

Table S9. Synteny analysis result for each bumblebee species relative to B. terrestris.

Table S10. TE loci reside within or close to protein-coding genes.

Table S11. dN/dS ratios of protein-coding genes whose coding regions contain TE-derived sequences.

Table S12. TEs located within open chromatin regions detected by ATAC-seq in B. terrestris.

Table S13. Bombus-specific genes.

Table S14. The universality of gene functional categories revealed by Kolmogorov–Smirnov (KS) analysis.

Table S15. Species-specific genes.

Table S16. Overall rate of gene gain/loss in Bombus genomes.

Table S17. Summary of gene gain and loss events across 15 bumblebee species.

Table S18. Enrichment analysis of top changing gene families in bumblebee revealed by CAFE.

Table S19. KS analysis on gene copy number variation across bumblebees.

Table S20. The top 40 InterPro domains with the most variable gene counts across bumblebees.

Table S21. List of B. terrestris stop-codon readthrough candidates.

Table S22. Intron presence, gain, and loss estimates from Malin analysis.

Table S23. Functional enrichment analysis of genes with the highest and lowest evolutionary rates.

Table S24. Functional enrichments of genes with the highest and lowest dN/dS ratios.

Table S25. The identification results of chemosensory genes in the 17 newly sequenced bumblebees.

Table S26. Positive selection analysis of chemosensory genes in bumblebee.

Table S27. The identification results of detoxification genes in bumblebees and comparisons with that of selected insects.

Table S28. Positive selection analysis of detoxification genes in bumblebees.

Table S29. The identification results of immune genes in bumblebees and comparisons with that of selected insects.

Table S30. Positive selection analysis of immune genes in bumblebees.

Table S31. Genes involved in high-elevation adaptation.

Table S32. Protein sequences used for MAFFT alignment to confirm the domestication of TEs in bumblebee.

Table S33. The summary of RNA-seq and transcriptome assembly.

# References

Altschul SF, Gish W, Miller W, Myers EW, Lipman DJ. 1990. Basic Local Alignment Search Tool. *J Mol Biol*. 215:403-410.

Altschul SF, Madden TL, Schaffer AA, Zhang J, Zhang Z, Miller W, Lipman DJ. 1997. Gapped BLAST and PSI-BLAST: a new generation of protein database search programs. *Nucleic acids research* 25:3389-3402.

Arbetman MP, Gleiser G, Morales CL, Williams P, Aizen MA. 2017. Global decline of bumblebees is phylogenetically structured and inversely related to species range size and pathogen incidence. *Proc Biol Sci*. 284:20170204.

Barribeau SM, Sadd BM, du Plessis L, Brown MJ, Buechel SD, Cappelle K, Carolan JC, Christiaens O, Colgan TJ, Erler S, et al. 2015. A depauperate immune repertoire precedes evolution of sociality in bees. *Genome Biol*. 16:83. doi: 10.1186/s13059-015-0628-y

Belton J-M, McCord RP, Gibcus JH, Naumova N, Zhan Y, Dekker J. 2012. Hi–C: a comprehensive technique to capture the conformation of genomes. *Methods* 58:268-276.

Benson G. 1999. Tandem repeats finder: a program to analyze DNA sequences. *Nucleic acids research*. 27:573-580.

Bonetti B, Fu L, Moon J, Bedwell DM. 1995. The Efficiency of Translation Termination is Determined by a Synergistic Interplay Between Upstream and Downstream Sequences inSaccharomyces cerevisiae. *J Mol Biol*. 251:334-345.

Camacho C, Coulouris G, Avagyan V, Ma N, Papadopoulos J, Bealer K, Madden TL. 2009. BLAST+: architecture and applications. *BMC bioinformatics* 10:421.

Cameron SA, Hines HM, Williams PH. 2007. A comprehensive phylogeny of the bumble bees ( Bombus ). *Biol J Linn Soc*. 91:161-188.

Cameron SA, Sadd BM. 2020. Global Trends in Bumble Bee Health. *Annu Rev Entomol*. 65:209-232.

Cantarel BL, Korf I, Robb SM, Parra G, Ross E, Moore B, Holt C, Alvarado AS, Yandell M. 2008. MAKER: an easy-to-use annotation pipeline designed for emerging model organism genomes. *Genome Res*. 18:188-196.

Capellagutierrez S, Sillamartinez JM, Gabaldon T. 2009. trimAl: a tool for automated alignment trimming in large-scale phylogenetic analyses. *Bioinformatics* 25:1972-1973.

Chen KC, Durand D, Farachcolton M. 2000. NOTUNG: A Program for Dating Gene Duplications and Optimizing Gene Family Trees. *J Comput Biol*. 7:429-447.

Criscuolo A, Gribaldo S. 2010. BMGE (Block Mapping and Gathering with Entropy): a new software for selection of phylogenetic informative regions from multiple sequence alignments. *BMC Evol Biol*. 10:210.

Csűros M. 2008. Malin: maximum likelihood analysis of intron evolution in eukaryotes. *Bioinformatics* 24:1538-1539.

De Bie T, Cristianini N, Demuth JP, Hahn MW. 2006. CAFE: a computational tool for the study of gene family evolution. *Bioinformatics* 22:1269-1271.

Dudchenko O, Batra SS, Omer AD, Nyquist SK, Hoeger M, Durand NC, Shamim MS, Machol I, Lander ES, Aiden AP. 2017. De novo assembly of the Aedes aegypti genome using Hi-C yields chromosome-length scaffolds. *Science* 356:92-95.

Durand NC, Shamim MS, Machol I, Rao SSP, Huntley MH, Lander ES, Aiden EL. 2016. Juicer Provides a One-Click System for Analyzing Loop-Resolution Hi-C Experiments. *Cell systems* 3:95-98.

Eaton DA, Ree RH. 2013. Inferring phylogeny and introgression using radseq data: An example from flowering plants (pedicularis: Orobanchaceae). *Syst Biol*. 62:689-706.

Edgar RC. 2004. MUSCLE: multiple sequence alignment with high accuracy and high throughput. *Nucleic acids research* 32:1792-1797.

Ellinghaus D, Kurtz S, Willhoeft U. 2008. LTRharvest, an efficient and flexible software for de novo detection of LTR retrotransposons. *BMC bioinformatics* 9:18.

Enright, J. A. 2002. An efficient algorithm for large-scale detection of protein families. *Nucleic acids research* 30:1575-1584.

Evans JD, Aronstein KA, Chen Y, Hetru C, Imler J, Jiang H, Kanost MR, Thompson GJ, Zou Z, Hultmark D. 2006. Immune pathways and defence mechanisms in honey bees Apis mellifera. *Insect Mol Biol*. 15:645-656.

Falcon S, Gentleman R. 2007. Using GOstats to test gene lists for GO term association. *Bioinformatics* 23:257-258.

Gertz EM, Yu Y-K, Agarwala R, Schäffer AA, Altschul SF. 2006. Composition-based statistics and translated nucleotide searches: Improving the TBLASTN module of BLAST. *BMC Biology* 4:41.

Haas BJ, Delcher AL, Mount MSMS, Wortman JR, Smith RKW, Hannick LI, Maiti R, Ronning CM, Rusch DB, Town CD. 2003. Improving the Arabidopsis genome annotation using maximal transcript alignment assemblies. *Nucleic acids research* 31:5654-5666.

Haas BJ, Papanicolaou A, Yassour M, Grabherr M, Blood PD, Bowden J, Couger MB, Eccles D, Li B, Lieber M, et al. 2013. *De novo* transcript sequence reconstruction from RNA-seq using the Trinity platform for reference generation and analysis. *Nat Protoc*. 8:1494-1512.

Han MV, Thomas GW, Lugo-Martinez J, Hahn MW. 2013. Estimating gene gain and loss rates in the presence of error in genome assembly and annotation using CAFE 3. *Mol Biol Evol*. 30:1987-1997. doi: 10.1093/molbev/mst100

Harris RS. 2007. Improved Pairwise Alignment of Genomic DNA. *PhD thesis*. Pennsylvania State Univ.

Heavens D, Accinelli GG, Clavijo B, Clark MD. 2015. A method to simultaneously construct up to 12 differently sized Illumina Nextera long mate pair libraries with reduced DNA input, time, and cost. *Biotechniques* 59:42-45. doi: 10.2144/000114310

Hebert PD, Penton EH, Burns JM, Janzen DH, Hallwachs W. 2004. Ten species in one: DNA barcoding reveals cryptic species in the neotropical skipper butterfly Astraptes fulgerator. *Proc Natl Acad Sci USA*. 101:14812-14817.

Hines HM. 2008. Historical biogeography, divergence times, and diversification patterns of bumble bees (Hymenoptera: Apidae: Bombus). *Syst Biol*. 57:58-75. doi: 10.1080/10635150801898912

Hoang DT, Chernomor O, Von Haeseler A, Minh BQ, Vinh LS. 2018. UFBoot2: Improving the Ultrafast Bootstrap Approximation. *Mol Biol Evol*. 35:518-522.

Hoede C, Arnoux S, Moisset M, Chaumier T, Inizan O, Jamilloux V, Quesneville H. 2014. PASTEC: An Automatic Transposable Element Classification Tool. *PLoS One* 9:e91929.

Hofacker IL. 2009. RNA Secondary Structure Analysis Using the Vienna RNA Package. *Curr Protoc Bioinformatics* 26:12.12. 11–12.12. 16.

Huson DH, Klöpper T, Lockhart PJ, Steel MA editors.; 2005 Berlin, Heidelberg.

Jones DT, Taylor WR, Thornton JM. 1992. The rapid generation of mutation data matrices from protein sequences. *Bioinformatics* 8:275-282.

Jones PH, Binns D, Chang H, Fraser M, Li W, Mcanulla C, Mcwilliam H, Maslen J, Mitchell AL, Nuka G. 2014. InterProScan 5: genome-scale protein function classification. *Bioinformatics* 30:1236-1240.

Jungreis I, Chan CS, Waterhouse RM, Fields G, Lin MF, Kellis M. 2016. Evolutionary dynamics of abundant stop codon readthrough. *Mol Biol Evol*. 33:3108-3132.

Jungreis I, Lin MF, Spokony R, Chan CS, Negre N, Victorsen A, White KP, Kellis M. 2011. Evidence of abundant stop codon readthrough in Drosophila and other metazoa. *Genome Res*. 21:2096-2113.

Junier T, Zdobnov EM. 2010. The Newick utilities: high-throughput phylogenetic tree processing in the UNIX shell. *Bioinformatics* 26:1669-1670. doi: 10.1093/bioinformatics/btq243

Kalyaanamoorthy S, Minh BQ, Wong TKF, Von Haeseler A, Jermiin LS. 2017. ModelFinder: fast model selection for accurate phylogenetic estimates. *Nat Methods*. 14:587-589.

Karpe SD, Jain R, Brockmann A, Sowdhamini R. 2016. Identification of Complete Repertoire of Apis florea Odorant Receptors Reveals Complex Orthologous Relationships with Apis mellifera. *Genome Biol Evol*. 8:2879-2895.

Katoh K, Misawa K, Kuma K, Miyata T. 2002. MAFFT: a novel method for rapid multiple sequence alignment based on fast Fourier transform. *Nucleic acids research* 30:3059-3066.

Kazutaka K, Standley DM. 2013. MAFFT Multiple Sequence Alignment Software Version 7: Improvements in Performance and Usability. *Mol Biol Evol*. 30:772-780.

Korf IF. 2004. Gene finding in novel genomes. *BMC bioinformatics* 5:59.

Kozlov AM, Darriba D, Flouri T, Morel B, Stamatakis A. 2019. RAxML-NG: a fast, scalable and user-friendly tool for maximum likelihood phylogenetic inference. *Bioinformatics* 35:4453-4455.

Kumar S, Stecher G, Li M, Knyaz C, Tamura K. 2018. MEGA X: Molecular Evolutionary Genetics Analysis across Computing Platforms. *Mol Biol Evol*. 35:1547-1549.

Langmead B, Salzberg S. Fast gapped-read alignment with Bowtie 2. 2012. *Nat Methods*. 9:357-359.

Li H, Durbin R. 2010. Fast and accurate long-read alignment with Burrows–Wheeler transform. *Bioinformatics* 26:589-595.

Li L, Stoeckert CJ, Roos DS. 2003. OrthoMCL: Identification of Ortholog Groups for Eukaryotic Genomes. *Genome Res*. 13:2178-2189.

Li W, Godzik A. 2006. Cd-hit: a fast program for clustering and comparing large sets of protein or nucleotide sequences. *Bioinformatics* 22:1658-1659.

Lin D, Hong P, Zhang S, Xu W, Jamal M, Yan K, Lei Y, Li L, Ruan Y, Fu ZF, et al. 2018. Digestion-ligation-only Hi-C is an efficient and cost-effective method for chromosome conformation capture. *Nat Genet*. 50:754-763. doi: 10.1038/s41588-018-0111-2

Lin MF, Jungreis I, Kellis M. 2011. PhyloCSF: a comparative genomics method to distinguish protein coding and non-coding regions. *Bioinformatics* 27:i275-i282.

Liu H, Jia Y, Sun X, Tian D, Hurst LD, Yang S. 2017. Direct Determination of the Mutation Rate in the Bumblebee Reveals Evidence for Weak Recombination-Associated Mutation and an Approximate Rate Constancy in Insects. *Mol Biol Evol*. 34:119-130. doi: 10.1093/molbev/msw226

Lomsadze A, Terhovhannisyan V, Chernoff YO, Borodovsky M. 2005. Gene identification in novel eukaryotic genomes by self-training algorithm. *Nucleic acids research* 33:6494-6506.

Love RR, Weisenfeld NI, Jaffe DB, Besansky NJ, Neafsey DE. 2016. Evaluation of DISCOVAR de novo using a mosquito sample for cost-effective short-read genome assembly. *BMC Genomics* 17:187. doi: 10.1186/s12864-016-2531-7

Lowe TM, Eddy SR. 1997. tRNAscan-SE: a program for improved detection of transfer RNA genes in genomic sequence. *Nucleic acids research* 25:955-964.

Minh BQ, Hahn MW, Lanfear R. 2020a. New methods to calculate concordance factors for phylogenomic datasets. *Mol Biol Evol*. msaa106. doi: 10.1093/molbev/msaa106

Minh BQ, Schmidt HA, Chernomor O, Schrempf D, Woodhams MD, Von Haeseler A, Lanfear R. 2020b. IQ-TREE 2: New models and efficient methods for phylogenetic inference in the genomic era. *Mol Biol Evol*. 37:1530-1534.

Murrell B, Wertheim JO, Moola S, Weighill T, Scheffler K, Pond SLK. 2012. Detecting Individual Sites Subject to Episodic Diversifying Selection. *PLoS Genet*. 8:e1002764.

Neafsey DE, Waterhouse RM, Abai MR, Aganezov SS, Alekseyev MA, Allen JE, Amon J, Arca B, Arensburger P, Artemov G, et al. 2015. Mosquito genomics. Highly evolvable malaria vectors: the genomes of 16 Anopheles mosquitoes. *Science* 347:1258522. doi: 10.1126/science.1258522

Notredame C, Higgins DG, Heringa J. 2000. T-Coffee: A novel method for fast and accurate multiple sequence alignment. *J Mol Biol*. 302:205-217.

Paten B, Earl D, Nguyen N, Diekhans M, Zerbino DR, Haussler D. 2011. Cactus: Algorithms for genome multiple sequence alignment. *Genome Res*. 21:1512-1528.

Persson AS, Rundlöf M, Clough Y, Smith HG. 2015. Bumble bees show trait-dependent vulnerability to landscape simplification. *Biodivers Conserv*. 24:3469-3489. doi: 10.1007/s10531-015-1008-3

Pertea M, Kim D, Pertea G, Leek JT, Salzberg SL. 2016. Transcript-level expression analysis of RNA-seq experiments with HISAT, StringTie and Ballgown. *Nat Protoc*. 11:1650-1667.

Price AL, Jones NC, Pevzner PA. 2005. De novo identification of repeat families in large genomes. *Bioinformatics* 21:351-358.

Prufer K, Stenzel U, Dannemann M, Green RE, Lachmann M, Kelso J. 2008. PatMaN: Rapid alignment of short sequences to large databases. *Bioinformatics* 24:1530-1531.

Rho M, Tang H. 2009. MGEScan-non-LTR: computational identification and classification of autonomous non-LTR retrotransposons in eukaryotic genomes. *Nucleic acids research* 37:e143.

Robertson HM, Wanner KW. 2006. The chemoreceptor superfamily in the honey bee, Apis mellifera: Expansion of the odorant, but not gustatory, receptor family. *Genome Res*. 16:1395-1403.

Ronquist F, Teslenko M, Mark Pvd, Ayres DL, Darling A, Höhna S, Larget B, Liu L, Suchard MA, Huelsenbeck JP. 2012. MrBayes 3.2: Efficient Bayesian Phylogenetic Inference and Model Choice Across a Large Model Space. *Syst Biol*. 61:539-542.

Sackton TB, Lazzaro BP, Schlenke TA, Evans JD, Hultmark D, Clark AG. 2007. Dynamic evolution of the innate immune system in Drosophila. *Nat Genet*. 39:1461-1468. doi: 10.1038/ng.2007.60

Sadd BM, Barribeau SM, Bloch G, Graaf DCd, Dearden P, Elsik CG, Gadau J, Grimmelikhuijzen CJ, Hasselmann M, Lozier JD, et al. 2015. The genomes of two key bumblebee species with primitive eusocial organization. *Genome Biol*. 16:76.

Sahlin K, Vezzi F, Nystedt B, Lundeberg J, Arvestad L. 2014. BESST-efficient scaffolding of large fragmented assemblies. *BMC bioinformatics*. 15:281.

Sanderson MJ. 2003. r8s: inferring absolute rates of molecular evolution and divergence times in the absence of a molecular clock. *Bioinformatics* 19:301-302.

Sikora A, Kelm M. 2012. Flower Preferences of the Wrocław Botanical Garden Bumblebees (Bombus spp.). *Journal of Apicultural Science* 56:27-36. doi: 10.2478/v10289-012-0021-y

Slater G, Birney E. 2005. Automated generation of heuristics for biological sequence comparison. *BMC bioinformatics* 6:31.

Smith MD, Wertheim JO, Weaver S, Murrell B, Scheffler K, Pond SLK. 2015. Less Is More: An Adaptive Branch-Site Random Effects Model for Efficient Detection of Episodic Diversifying Selection. *Mol Biol Evol*. 32:1342-1353.

Stamatakis A. 2014. RAxML version 8: a tool for phylogenetic analysis and post-analysis of large phylogenies. *Bioinformatics* 30:1312-1313.

Stanke M, Schoffmann O, Morgenstern B, Waack S. 2006. Gene prediction in eukaryotes with a generalized hidden Markov model that uses hints from external sources. *BMC bioinformatics* 7:62.

Steinbiss S, Willhoeft U, Gremme G, Kurtz S. 2009. Fine-grained annotation and classification of de novo predicted LTR retrotransposons. *Nucleic acids research* 37:7002-7013.

Subramanian S. 2008. Nearly Neutrality and the Evolution of Codon Usage Bias in Eukaryotic Genomes. *Genetics* 178:2429-2432.

Suyama M, Torrents D, Bork P. 2006. PAL2NAL: robust conversion of protein sequence alignments into the corresponding codon alignments. *Nucleic acids research* 34:609-612.

Talavera G, Castresana J. 2007. Improvement of Phylogenies after Removing Divergent and Ambiguously Aligned Blocks from Protein Sequence Alignments. *Syst Biol*. 56:564-577.

Tesler G. 2002. Efficient algorithms for multichromosomal genome rearrangements. *J Comp Sys Sci*. 65:587-609.

Vanderpool D, Minh BQ, Lanfear R, Hughes D, Murali S, Harris RA, Raveendran M, Muzny DM, Gibbs RA, Worley KC, et al. 2020. Primate phylogenomics uncovers multiple rapid radiations and ancient interspecific introgression. *bioRxiv* 2020.2004.2015.043786.

Vicario S, Moriyama EN, Powell JR. 2007. Codon usage in twelve species of Drosophila. *BMC Evol Biol*. 7:226. doi: 10.1186/1471-2148-7-226

Wang G, Yin H, Li B, Yu C, Wang F, Xu X, Cao J, Bao Y, Wang L, Abbasi AA. 2019. Characterization and identification of long non-coding RNAs based on feature relationship. *Bioinformatics* 35:2949-2956.

Wang L, Park HJ, Dasari S, Wang S, Kocher JA, Li W. 2013. CPAT: Coding-Potential Assessment Tool using an alignment-free logistic regression model. *Nucleic acids research* 41:e74.

Wang W, Ashby R, Ying H, Maleszka R, Foret S. 2017. Contrasting sex-and caste-dependent piRNA profiles in the transposon depleted haplodiploid honeybee Apis mellifera. *Genome Biol Evol*. 9:1341-1356.

Wang Y, Tang H, D DJ, Xu T, Li J, Wang X, Tae-ho L, Jin H, Barry M, Hui G. 2012. MCScanX: a toolkit for detection and evolutionary analysis of gene synteny and collinearity. *Nucleic acids research* 40:e49.

Waterhouse RM, Lazzaro BP, Sackton TB. 2020. Characterization of Insect Immune Systems from Genomic Data. *Immunity in Insects* 3-34.

Waterhouse RM, Seppey M, Simão FA, Manni M, Ioannidis P, Klioutchnikov G, Kriventseva EV, Zdobnov EM. 2018. BUSCO applications from quality assessments to gene prediction and phylogenomics. *Mol Biol Evol*. 35:543-548.

Waterhouse RM, Zdobnov EM, Tegenfeldt F, Li J, Kriventseva EV. 2011. OrthoDB: the hierarchical catalog of eukaryotic orthologs in 2011. *Nucleic Acids Res*. 39(Database issue): D283-D288.

Wertheim JO, Murrell B, Smith MD, Pond SLK, Scheffler K. 2015. RELAX: Detecting Relaxed Selection in a Phylogenetic Framework. *Mol Biol Evol*. 32:820-832.

Williams, Paul H. 1985. A preliminary cladistic investigation of relationships among the bumble bees (Hymenoptera, Apidae). *Systematic Entomology* 10:239-255.

Williams PH. 1994. Phylogenetic relationships among bumble bees (Bombus Latr.): a reappraisal of morphological evidence. *Systematic Entomology* 19:327-344.

Williams PH, Cameron SA, Hines HM, Cederberg B, Rasmont P. 2008. A simplified subgeneric classification of the bumblebees (genusBombus). *Apidologie* 39:46-74. doi: 10.1051/apido:2007052

Yang Z. 2007. PAML 4: Phylogenetic Analysis by Maximum Likelihood. *Mol Biol Evol*. 24:1586-1591.

Yang Z, Wong WSW, Nielsen R. 2005. Bayes Empirical Bayes Inference of Amino Acid Sites Under Positive Selection. *Mol Biol Evol*. 22:1107-1118.

Ye J, Fang L, Zheng H, Zhang Y, Chen J, Zhang Z, Wang J, Li S, Li R, Bolund L. 2006. WEGO: a web tool for plotting GO annotations. *Nucleic acids research* 34:293-297.

Yu G, Smith DK, Zhu H, Guan Y, Lam TT. 2017. ggtree: an R package for visualization and annotation of phylogenetic trees with their covariates and other associated data. *Methods in Ecology and Evolution* 8:28-36.

Zhang J, Nielsen R, Yang Z. 2005. Evaluation of an Improved Branch-Site Likelihood Method for Detecting Positive Selection at the Molecular Level. *Mol Biol Evol*. 22:2472-2479.

Zhang C, Rabiee M, Sayyari E, Mirarab S. 2018. ASTRAL-III: polynomial time species tree reconstruction from partially resolved gene trees. *BMC bioinformatics* 19:15-30.

Zhao X, Xu W, Schaack S, Sun C. 2019. Genome-wide identification of accessible chromatin regions in bumblebee (Bombus terrestris) by ATAC-seq. *bioRXiv* 818211.

Zhou X, Rokas A, Berger SL, Liebig J, Ray A, Zwiebel LJ. 2015. Chemoreceptor Evolution in Hymenoptera and Its Implications for the Evolution of Eusociality. *Genome Biol Evol*. 7:2407-2416.

Zhou X, Slone J, Rokas A, Berger SL, Liebig J, Ray A, Reinberg D, Zwiebel LJ. 2012. Phylogenetic and Transcriptomic Analysis of Chemosensory Receptors in a Pair of Divergent Ant Species Reveals Sex-Specific Signatures of Odor Coding. *PLoS Genet*. 8:e1002930.
